# Roles of glutamic pyruvate transaminase 2 in reprogramming of airway epithelial lipidomic and metabolomic profiles after smoking

**DOI:** 10.1002/ctm2.1679

**Published:** 2024-05-05

**Authors:** Furong Yan, Linlin Zhang, Lian Duan, Liyang Li, Xuanqi Liu, Yifei Liu, Tiankui Qiao, Yiming Zeng, Hao Fang, Duojiao Wu, Xiangdong Wang

**Affiliations:** ^1^ Center for Tumor Diagnosis & Therapy Jinshan Hospital Fudan University Shanghai China; ^2^ Department of Pulmonary and Critical Care Medicine Zhongshan Hospital Fudan University Shanghai China; ^3^ Center of Molecular Diagnosis and Therapy The Second Affiliated Hospital of Fujian Medical University Quanzhou Fujian China; ^4^ Department of Pediatric Surgery Faculty of Pediatrics the Seventh Medical Center of PLA General Hospital Beijing China; ^5^ Department of Anesthesiology Shanghai Geriatic Medical Center Shanghai China; ^6^ Department of Anesthesiology Zhongshan Hospital, Fudan University Shanghai China

**Keywords:** chronic obstructive pulmonary disease, cigarette smoking, glutamic pyruvate transaminase 2, lipidomics

## Abstract

Metabolic abnormalities represent one of the pathological features of chronic obstructive pulmonary disease (COPD). Glutamic pyruvate transaminase 2 (GPT2) is involved in glutamate metabolism and lipid synthesis pathways, whilst the exact roles of GPT2 in the occurrence and development of COPD remains uncertain. This study aims at investigating how GPT2 and the associated genes modulate smoking‐induced airway epithelial metabolism and damage by reprogramming lipid synthesis. The circulating or human airway epithelial metabolomic and lipidomic profiles of COPD patients or cell‐lines explored with smoking were assessed to elucidate the pivotal roles of GPT2 in reprogramming processes. We found that GPT2 regulate the reprogramming of lipid metabolisms caused by smoking, especially phosphatidylcholine (PC) and triacylglycerol (TAG), along with changes in the expression of lipid metabolism‐associated genes. GPT2 modulated cell sensitivities and survival in response to smoking by enhancing mitochondrial functions and maintaining lipid and energy homeostasis. Our findings provide evidence for the involvement of GPT2 in the reprogramming of airway epithelial lipids following smoking, as well as the molecular mechanisms underlying GPT2‐mediated regulation, which may offer an alternative of therapeutic strategies for chronic lung diseases.

## Graphical abstract

1. GPT2 regulated smoking‐induced metabolism and damage in airway epithelial cells by affecting lipid synthesis, especially PCs and TAGs.
2. GPT2‐dominated lipid metabolism enhanced mitochondrial function and maintained lipid and energy homeostasis.
3. Interactions between lipid metabolism genes, PI3K subset genes, and metabolites contributed to the development of GPT2‐regulated lipid reprogramming during smoking.

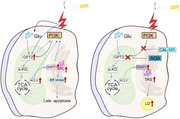

## 1 INTRODUCTION

Chronic obstructive pulmonary disease (COPD) is characterised by airway and lung inflammation, remodelling, and declined lung function, making it the fifth leading cause of death worldwide.^1^ Smoking is one of the common and critical factors for the induction and development of COPD and activates the interactions amongst multiple lung cells.^1^, ^2^ The airway epithelial cells serve as the initial defense separating the tissue microenvironment from the external environment, the primary receptors to face and bind the pathogens, and as the secondary inducer to result in localised and systemic inflammation, pulmonary dysfunction, and tissue impairment in COPD, as a prominent characteristic of the disease.^3^, ^4^, ^5^ In addition, lung epithelial cells play crucial roles in preserving lung function and alveolar structures and are the main source to generate potential targets for therapeutic interventions to reduce inflammation and remodelling in COPD.

The metabolic homeostasis between systemic and lung microenvironments balances cell functions and interactions, where lipids can be converted from carbohydrates or amino acid metabolites. The comprehensive abnormalities in local and systemic metabolisms detected by metabolomics and lipidomics are important factors in the pathogenesis of COPD.^6^, ^7^ Of metabolites, L‐glutamic acid (glutamate) has multiple functions and contributes to the generation of energy, processes of protein folding, de novo synthesis of nucleic acids and lipid synthesis.^8^, ^9^ Glutamate metabolism pathways play important roles in the maintenance of pulmonary functions and the pathogenesis of chronic lung diseases.^10^, ^11^, ^12^, ^13^ Glutamic pyruvate transaminase (GPT, also named alanine aminotransferase) is responsible for the formation of α‐ketoglutaric acid (α‐KG) from glutamate, of which GPT1 is mostly located in the cytoplasm, whilst GPT2 mainly within the mitochondria.^14^, ^15^ The α‐KG is an important raw material for lipid synthesis. Cigarette smoke causes abnormal lipid metabolism in airway epithelial cells.^16^ The present studies combined database data with our experimental data and found significant differences in intracellular metabolic pathways for alanine, aspartate, and glutamate in airway epithelial cells after cigarette stimulation. Of which, glutamate showed the most significant change in the disease. To investigate whether altered glutamate metabolism is an important part of cigarette‐induced abnormalities in cellular lipid metabolism, we screened key lipid‐associated genes on the pathway for the synthesis of lipids from glutamate and found that GPT2 plays the most significant role.

This study aims at exploring the potential mechanisms by which the GPT2‐mediated glutamate pathway impacts smoking‐induced airway epithelial cell damage by reprogramming lipid synthesis. We defined the abnormality of circulating metabolomic and lipidomic profiles in COPD patients and the glutamate‐dominated metabolism dysfunction in airway epithelial cells challenged with cigarette smoking extract (CSE) by monitoring intra‐ and extra‐epithelial profiles. We assessed the regulatory roles of GPT2 in the expression of CSE‐altered lipid metabolism‐associated genes, glutamate‐to‐lipid synthesis and apoptosis of airway epithelial cells. Our data indicate that GPT2 controls the airway epithelial cell function by regulating endoplasmic reticulum (ER) stress, mitochondrial function, and lipid metabolism. To further understand regulatory mechanisms amongst glutamate metabolism‐associated genes, we measured the inter‐regulations of those genes with changes of the corresponding metabolisms and with phosphoinositide‐3‐kinase (PI3K) subunit genes in airway epithelial cells during CSE. Our data evidence the important roles of GPT2 and corresponding metabolism in airway epithelial cell dysfunction and chronic lung injury and indicate an alternative potential therapy for COPD by reprogramming glutamate‐dominated metabolisms.

## 2 METHODS AND MATERIALS

### 2.1 Patient population

This study was approved the Ethical Evaluation Committee of Zhongshan Hospital [B2019‐197(2)]. Twelve COPD patients and healthy volunteers were admitted to the Zhongshan Hospital between October 2019 and March 2020, respectively, with the informed consent for the study. The plasma from healthy volunteers and COPD patients was collected on the first day of their admission. The information on patient populations was detailed in Table S1.

### 2.2 Cell culture, transfection, and RNA interference

Human bronchial epithelial cell lines (16‐HBE cells) were purchased from ATCC, USA, and cultured in the RPMI‐1640 medium supplemented with 1% (v/v) cell growth factor, 1% (v/v) penicillin, 1% (v/v) streptomycin (Shanghai Zhongqiao Xinzhou Biotechnology Co., Shanghai, China), and 10% fetal bovine serum (#10099141, Gibco, ThermoFisher, MA, USA). Aminooxyacetate (AOA, C13408; an inhibitor of aminobutyrate aminotransferase) and 4‐phenylbutyric acid (4‐PBA, P21005; a reducer of ER stress) were obtained from Sigma (Sigma–Aldrich, MO, USA). CAL‐101 (S2226, p110δ inhibitor), CAY10505 (S2682, PI3Kγ inhibitor), LY294002 (S1105, PI3Kα/δ/β inhibitor), GDC0941 (S1065, PI3Kα/δ inhibitor), HS173 (S7356, PI3Kα inhibitor) and SB216763 (S1075, GSK‐3α inhibitor) from Shelleck (Shelleck Chemicals, TX, USA). SHBM1009 is a new p110α/γ/δ/β inhibitor and synthesised by Fudan University.^17^, ^18^

Lipofectamine 3000 (#L3000015) was purchased from Thermo Fisher Scientific, MA, USA, for transfection of RNA interference and overexpression of target genes [e.g., GPT2, adenosine triphosphate (ATP) citrate lyase (ACLY), choline phosphotransferase 1 (CHPT1), diacylglycerol O‐acyltransferase 1 (DGAT1), and phosphatidylinositol‐4,5‐bisphosphate 3‐kinase catalytic subunit delta (PIK3CD)]. The efficacies of knockdown and overexpression were verified 24 h after the cell culture. The HBE cells were treated with solute carrier family 7 member 11 (SLC7A11), GPT2, ACLY, CHPT1, DGAT1, and PIK3CD siRNA and presented as cell*^siSLC7A11^*, cell*^siGPT2^*, cell*^siACLY^*, cell*^siCHPT1^*, cell*^siDGAT1^*, and cell*^siPIK3CD^*, respectively, whilst cells with non‐specific siRNA as cell*^NC^*. The sequences of interfered genes were listed in Table S2.

### 2.3 Preparation of cigarette smoking extract

Cigarettes were combusted using a modified 50 mL syringe apparatus, in the presence of 5 mL of RPMI‐1640 medium devoid of serum. The quality of CSE was assessed by quantifying the absorbance value at a wavelength of 320λ (1.158), as reported previously.^2^, ^3^, ^19^

### 2.4 Establishment of animal cigarette smoking extract ‐induced chronic obstructive pulmonary disease model

Wild‐type C57BL/6J mice (6−8 weeks old) were purchased from Sibeifu Beijing Biotechnology Co., Beijing, China, and housed in a temperature‐ and humidity‐controlled room of Shanghai Public Health Clinical Center Animal Experiment Center at Shanghai, China. To meet the experimental requirements, a definite CSE preparation method was employed for the induction of animal modelling. The cigarettes were inserted into the end of the rubber tube and placed in a well‐vented fume hood, after removing the filter tips. A 50 mL syringe was connected to the other end of the tube, the syringe was aspirated repeatedly, and the cigarette smoking was poured into a 50 mL tube containing 4 mL phosphate‐buffered saline (PBS). The cigarette smoking was completely dissolved in PBS, particles and bacteria were filtered with a.2 μM pore size filter, and the pH of CSE was adjusted to 7.2–7.4. The CSE was freshly prepared half an hour before each airway installation. The cigarettes used in the experiment contained 10 mg tar, 1.1 mg nicotine and 12 mg carbon monoxide per cigarette.

The mice were intraperitoneally injected with 200 mg 2.5% avertin (T48402, Sigma, MO, USA) for anaesthesia and intratracheally instilled with CSE. The CSE‐induced COPD mice were randomly divided into three groups (*n *= 5 mice per group), including animals treated with PBS and challenged with vehicle, treated with vehicle and challenged with 100%CSE at 30 μL/day for 5 days per week for 6 weeks, or treated with AOA at 10 mg/kg/day for 5 days per week for 6 weeks and challenged with 100% CSE at 30 μL/day for 5 days per week for 6 weeks. Mice were sacrificed and lung tissues were collected according to ethical guidelines. The right lung lobe was placed in a 4% paraformaldehyde tube for H&E staining, pathological scoring and TUNEL analysis. The left lung lobe was lavaged with PBS under the pressure of 20 cm H_2_O and the lavage fluid was collected into cryopreservation tubes and snap‐frozen in liquid nitrogen for lipidomics, metabolomics and RNA measurements.^20^, ^21^, ^22^, ^23^, ^24^

### 2.5 Assessment of cell apoptosis

The lung tissue sections were deparaffinised, rehydrated and permeabilised by proteinase K to assess apoptosis (In Situ Apoptosis Kit, Abcam, Cambridge, UK). In accordance with the manufacturer's instructions, the sections were labelled with TUNEL reagents after washing. Diaminobenzidine was used for the development, and apoptotic cells were examined and analysed using Image Pro Plus v.6.0 Software (Media Cybernetics, MD, USA). A TUNEL‐positivity index was calculated by dividing the total number of airway epithelia by the number of TUNEL‐positive cells.

### 2.6 Assessment of lung injury

The mice lung tissues were immersed in a 4% paraformaldehyde solution, embedded in paraffin, sectioned and deparaffinised with xylene. The slices were stained with hematoxylin solution, dehydrated, and stained with alcohol eosin solution. The pathological assessment of lung injury included pulmonary interstitial oedema, alveolar oedema, atelectasis, or alveolar haemorrhage (0: normal, 1: changes less than 25% visual field, 2: between 25%−50%, or 3: >50%), inflammatory cell infiltration (0: normal, 1: a small amount of infiltration, 2: moderate degree of infiltration, or 3: severe infiltration in the interstitial or alveolar spaces), or hyaline membrane formation (0: normal, 1: hyaline membrane formation less than 5% visual field, 2: between 5%−25%, or 3: >25%).

### 2.7 Measurement of metabolomic profiles

Plasma, lung tissue suspension and cell culture medium at 100 μL were prepared and transferred into a 1.5 mL tube. Cultured cells were sampled, prepared, collected, normalised to 2 × 10^6^ cells/sample, and then stored at −80°C for use. The cell membrane was permeabilised by repeatedly freeze‐thawed processes. Equal amounts of samples were pooled and prepared for the quality control (QC). The solution with the mixing of methanol:water (4:1) at 400 μL and tridecanoic acid at 5 μg/mL was added to samples as the internal standard. The solution was vigorously mixed at 37°C for 30 s and centrifugated at 1200 rpm for 30 min. The supernatant was lyophilised and collected after samples were centrifuged at 14000 rpm for 15 min at 4°C. According to the protocol of two‐step derivatisation, samples were resuspended in methoxopyridine hydrochloride at 50 μL of 20 mg/mL, vortexed, sonicated for 1 min, and incubated at 30°C for 90 min, to form methoxamine derivatives. *N*‐methyl‐*N* ‐(trimethylsilyl) trifluoroacetamide at 40 μL was added to the sample and incubated at 37°C for 30 min for salinisation. The supernatant was collected and placed in an upper sample tube, and the untargeted metabolomics analysed with the gas chromatography–mass spectrometry (GC–MS) (Agilent 7890B GC and 5977B inert mass selective detector (MSD) system, Agilent Technologies, Santa Clara, CA, USA). The MassHunter Qualitative Analysis software (version 10.0, Agilent) was used to analyse raw GC–MS data. Metabolites were identified using the Agilent Fiehn database.

### 2.8 Measurement of lipidomic profiles

Approximately 20 μL samples of plasma, lung tissue suspension, and cell culture medium were prepared and subsequently transferred into a 1.5 mL tube, followed by an introduction of pre‐cooled isopropanol (350 μL) and the internal standard 10 μL were introduced. After mixing, the samples were kept at room temperature for 10 min, stayed at −20°C overnight, and centrifuged for 15 min at 13000 rpm. The supernatant at 200 μL was collected, from which 10 μL was mixed into a QC sample tube for detection.^25^ Cultured cells were sampled, prepared, collected and normalised to 2 × 10^6^ cells/sample. After cell membrane permeabilisation, equal amounts of samples were pooled and prepared for QC. Water at 900 μL was added to the sample, transferred to a glass vial, and vortexed for 30 s. The mixture solution of chloroform: methanol at 1:2 (v: v) was added, vortexed for 1 min, and added with chloroform at 1 mL and 10 μL internal standard during vortexing. The solution was added with water at 1 mL, vortexed for 1 min, and centrifuged at 2000 rpm for 15 min. Of stratifications appeared after the centrifugation of samples, the lower layer of liquid was removed into a new tube, lyophilised, and re‐dissolved in a 200 μL mixture solution of chloroform: methanol with 10 mM ammonium acetate. Samples were centrifuged at 13000 rpm for 15 min, and the supernatant was removed and transferred in a tube for liquid chromatography–mass spectrometry (LC–MS) analysis.^26^ The internal standards were subscribed from the Internal Standards Kit for Lipidyzer Platform (SCIEX, Darmstadt, Germany).

We analysed lipidomic profiles using an AB SCIEX QTRAP 5500 LC–MS/MS system (Foster City, CA, USA). UPLC BEH HILIC column (100 mm × 2.1 mm, 1.7 m), and Waters Acquity UPLC BEH HILIC VanGuard Pre column (2.1 mm × 5 mm, 1.7 m) were used for the analysis of the extracted samples. The A phase was 95% acetonitrile (acetonitrile:water, v:v: 95:5) containing 10 mmol/L ammonium acetate, and the B phase was 50% acetonitrile (acetonitrile:water, v:v: 50:50). We added ammonium hydroxide to the B phase until the pH equalled to that of the A phase. The flow rate of the LC–MS system was.5 mL/min. B phase started with.1% and gradually increased to 20% during 10 min and 98% during 10–11 min. About 98% of the B phase was held for 2 min and returned to the initial condition at.1% in about 13 min. The.1% of the B phase was held for 16 min. The positive and negative electrospray ionisation (ESI+ and ESI−) mode injection volumes were 2 and 5 μL, respectively. The N_2_ was used as a dissolvent. Parameter settings included curtain gas at 35 psi, GS1 at 50 psi, GS2 at 60 psi, ion spray voltage at 5500 V, decluttering potential at 80 V, entrance energy at 10 V and collision energy at 50 V. Data were acquired using the Analyst software (version 1.7, SCIEX, MA, USA).

### 2.9 Flow fluorescence detection

After culturing cells in 12‐well plates, gene interventions, CSE stimulations and reagent additions were performed at different time points and groups. The Mito‐Tracker Red CMXRos (C1049B, Beyotime Biotechnology, Shanghai, China), DCFH‐DA from reactive oxygen species (ROS) Assay Kit (S0033S, Beyotime Biotechnology, Shanghai, China), and BODIPY 493/503 (GC42959, GLPBIO, CA, USA) were added and incubated for 20 min at 37°C. For detection of apoptosis and necrosis, cells were re‐suspended in a buffer containing 5 μL Annexin V‐fluorescein isothiocyanate (FITC) and 5 μL PI (#556,547, BD Bioscience, CA, USA) and stained for 15 min at room temperature in the dark. The cells were transferred to a flow tube and measured using the flow cytometer (BD FACS Aria II, USA). Data were analysed using the Flow Jo v10 software (Ashland, OR, USA).

### 2.10 Cell proliferation

Incubation of cells for 1 h at 37°C with 5% CO_2_ was followed by the treatment with CCK8 (KGA317, KeyGEN, Jiangsu, China) at 10 μL/well, measurement at 450 nm with a microplate absorbance reader, and recording of the results were performed after the cell culture.

### 2.11 ATP detection

Cells were cultured in 6‐well plates for 24 h, digested after gene intervention and CSE stimulation for 24 h, and transferred into 96‐well culture plates at 10^4^ cells/well. After cells were fully adherent using the ATPlite step (#6016736, PerkinElmer, MA, USA), 100 μL of recombinant reagent was added to each well. Four gradients of ATP standard solutions were established and the 96‐well microplate was shaken for 2 min for luminescence assays.

### 2.12 RNA sequencing

Cell integrity was assessed using the RNA Nano 6000 assay kit of the Bioanalyzer 2100 system (Agilent Technologies, CA, USA). Cells were purified with an AMPure XP system (Beckman Coulter, Beverly, USA), followed by sequencing on the Illumina Novaseq platform. To calculate the FPKM of each gene, read numbers from each mapped gene were counted using FeatureCounts v1.5.0‐p3. A reference genome was aligned using Hisat2 v2.0.5. DESeq2 R package (1.20.0) was used to analyse DEGs. Gene set enrichment analysis (GSEA) using gene sets such as Hallmark, Gene Ontology (GO), kyoto encyclopedia of genes and genomes (KEGG) and Reactome pathway enrichment were performed using the OmicShare cloud platform (www.omicshare.com/).

### 2.13 Quantitative real‐time PCR

Total RNA was extracted using TRIzol (#15596026, Invitrogen, CA, USA) and then reversely transcribed from mRNA to cDNA using PrimeScript RT Master Mix (#RR036A, Takara, Japan). TB Green Premix Ex Taq (RR420A, Takara, Japan) was used for real‐time PCR. The primer sequences used are listed in Table S2.

### 2.14 Western blot

The cells were lysed with ice‐cold lysis buffer containing protease inhibitors and phosphatase inhibitors. Supernatants were quantified using the BCA kit (P0010, Beyotime, Shanghai, China), loaded per lane in 10% sodium dodecyl sulfate polyacrylamide gel electrophoresis (SDS‐PAGE), and electrophoresed on a polyvinylidene fluoride (PVDF) membrane. Primary antibodies are as follows: GAPDH (KC‐5G4, 1:1000, KangChen Bio‐Tech, Shanghai, China), GPT2 (TA368451, 1:1000, OriGene, Beijing, China), ACLY (ab40793, 1:1000, Abcam, Cambridge, UK), DGAT1 (A6857, 1:1000, Abclonal, Wuhan, China), and anti‐mouse/rabbit HRP‐linked IgG antibodies (1:2000, #7076/#7074, Molecular Devices, MA, USA), as secondary antibodies. The PageRuler Prestained Protein Ladders were purchased and the bound antibodies were detected (#26616 and #32106, ThermoFisher). Image J (NIH, Bethesda, MD, USA) was used to calculate gray values.

### 2.15 Target validation in airway epithelial subsets

To furthermore evaluate the expression of target genes in human airway epithelia and the specificity of COPD, we downloaded lung tissue single‐cell RNA sequencing (scRNA‐*seq*) data from three datasets (GSE128169, GSE131907 and GSE136831; http://www.ncbi.nlm.nih.gov/geo/), including 15 cases of idiopathic fibrosis, 15 COPD, 8 systemic sclerosis‐associated interstitial lung disease, 15 lung adenocarcinoma, 20 healthy control or 11 para‐cancerous lung tissues. The raw datasets were transformed into gene expression matrices through Cellrangre3.0.0 (10× Genomics). Each sample information was converted into the Seurat objects, using the Seurat R package (Version 3.0) for performing cell annotation and obtaining 57 cell types. Of those, target gene expression of various lung epithelial single cells (e.g., alveolar epithelial types 1 and 2, basal, ciliated, club, goblet, and mucous epithelia) were sorted and compared amongst lung diseases by deep‐mining of scRNA‐*seq*. The signalling pathways and DEGs related to lipid metabolism were enriched, analysed and visualised using a free online platform (http://www.bioinformatics.com.cn).

### 2.16 Statistical analysis

To ensure the dependability and precision of our mass spectrometry data, we employed supplementary measures alongside the QC overlay plots. These measures encompassed the incorporation of internal standards, regular calibration assessments of the instruments, and the adoption of the relative standard deviation (RSD) as a metric for evaluating the accuracy and precision of the outcomes. To quantify data concerning internal standard data after lipid normalisation, Analyst software was used. SCIEX OS software was used to quantify target lipid concentrations according to the area and concentration of the internal standard/target lipid concentration (target lipid detection peak area/internal standard detection peak area × internal standard concentration). The metabolite concentrations were calculated by the formula: standard sample peak area/standard sample concentration = sample peak area/sample concentration. A usable dataset at an RSD of 30% (RSD = standard deviation/average value) was used to detect lipid and metabolite levels. Data were presented as mean ± standard errors. We used the Student's *t*‐test with two tails were applied for the significance of differences between groups after a one‐way analysis of variance. The pie charts, bubble plots, bar plots and heat maps were analysed and visualised by http://www.bioinformatics.com.cn. The gene networks were constructed using the package graph in Rstudio. Signalling pathways were enriched by an OMICSHARE (https://www.omicshare.com/tools/). We employed KEGG, GO, Hallmark and Reactome databases/resources for pathway enrichment analysis of differentially expressed genes or differentially regulated metabolites. Correlation analysis was adopted using Pearson's correlation. The *p*‐values less than .05 stand for the statistical significance.

## 3 RESULTS

### 3.1 Lipidomic and metabolomic profiles between healthy and chronic obstructive pulmonary disease patients

**FIGURE 1:**
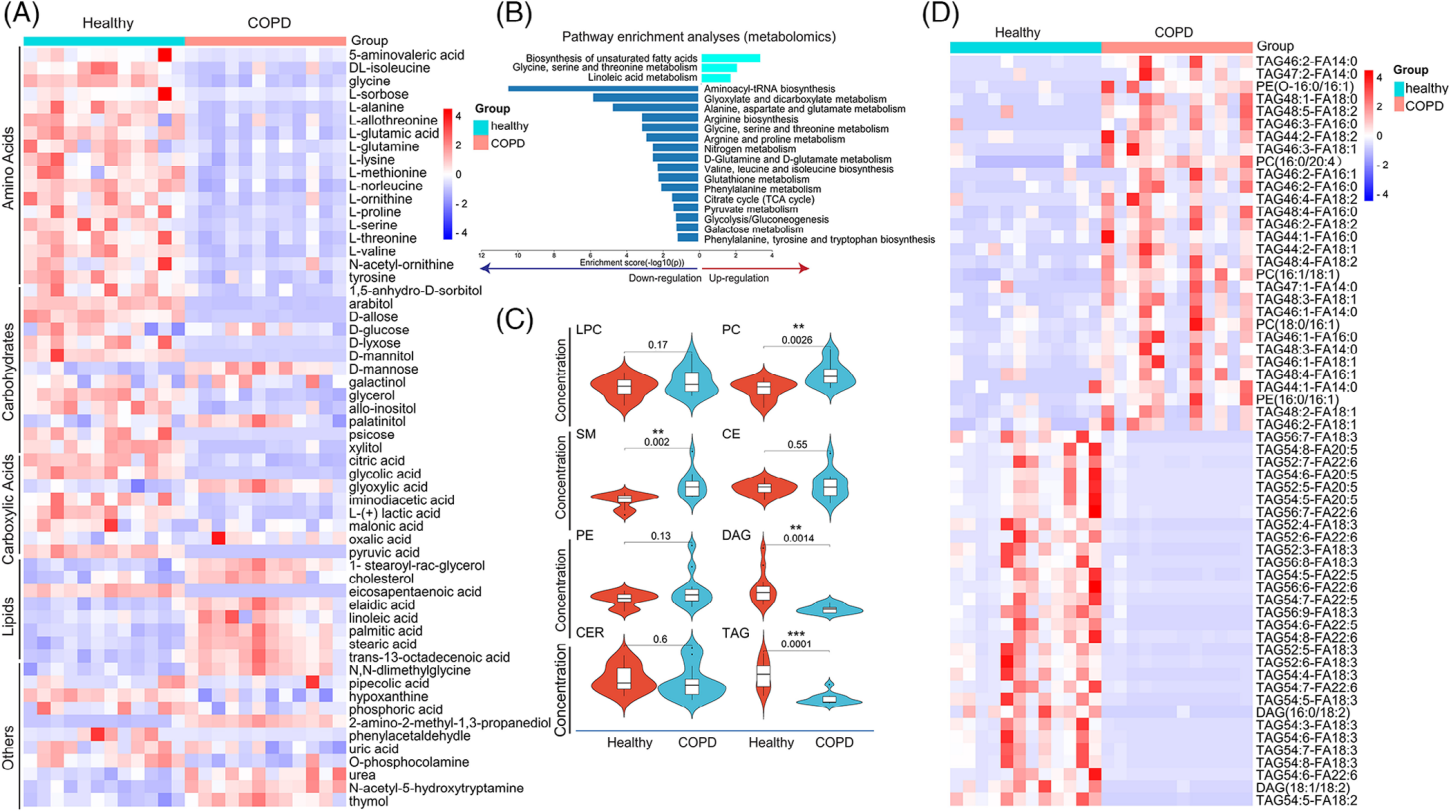
Metabolomic and lipidomic changes in plasma of chronic obstructive pulmonary disease (COPD) patients. Plasma samples were collected from healthy (*n* = 12) and COPD patients (*n* = 12) for measuring metabolite and lipid profiles. The metabolomic heat map (*n* = 12/group) demonstrated that COPD patients had higher levels of lipids in the circulation (A). KEGG pathway enrichment of differential metabolites showed that the metabolism of lipids and amino acids was the main enriched pathway (B). Plasma lipidomic profiles (*n* = 12/group) illustrate that COPD patients had higher levels of lysophosphatidyl cholines (LPC), phosphatidyl cholines (PC), phosphatidyl ethanolamine (PE), diacylglycerol (DAG), sphingomyelin (SM), cholesterol ester (CE), ceramide (CER) and triacylglyceride (TAG) (C). The lipidomic heatmap showed the top 30 of the most obvious up‐and down‐regulated lipids between the healthy and COPD (D).

We collected plasma samples from 12 healthy and 12 COPD patients and measured the metabolite and lipid profiles of the samples. Plasma levels of 46 metabolites in COPD patients significantly differed from healthy, including 17 amino acids, 10 carbohydrates, 7 carboxylic acids, 7 lipids, or 5 other small classes of metabolites, as detailed in Table S3. The heat map represents metabolite changes between COPD patients and healthy (Figure 1A). The enriched KEGG pathways were mainly related to lipids and amino acids metabolism, especially dysfunctional metabolisms of amino acids and lipids in COPD patients (Figure 1B). Levels of eight major lipid classes were compared between healthy and COPD, including lysophosphatidyl cholines (LPC), phosphatidyl cholines (PC), phosphatidyl ethanolamine (PE), diacylglycerol (DAG), sphingomyelin (SM), cholesterol ester (CE), ceramide (CER), and triacylglyceride (TAG) (Figure 1C). Levels of PC and SM significantly increased, whilst DAG and TAG decreased in COPD patients, as compared with the healthy. The distribution of differential lipids is presented in Figure 1D. Detailed differences for lipids are shown in Table S4.

### 3.2 Cigarette smoking extract ‐induced metabolomic changes in airway epithelial cells

**FIGURE 2:**
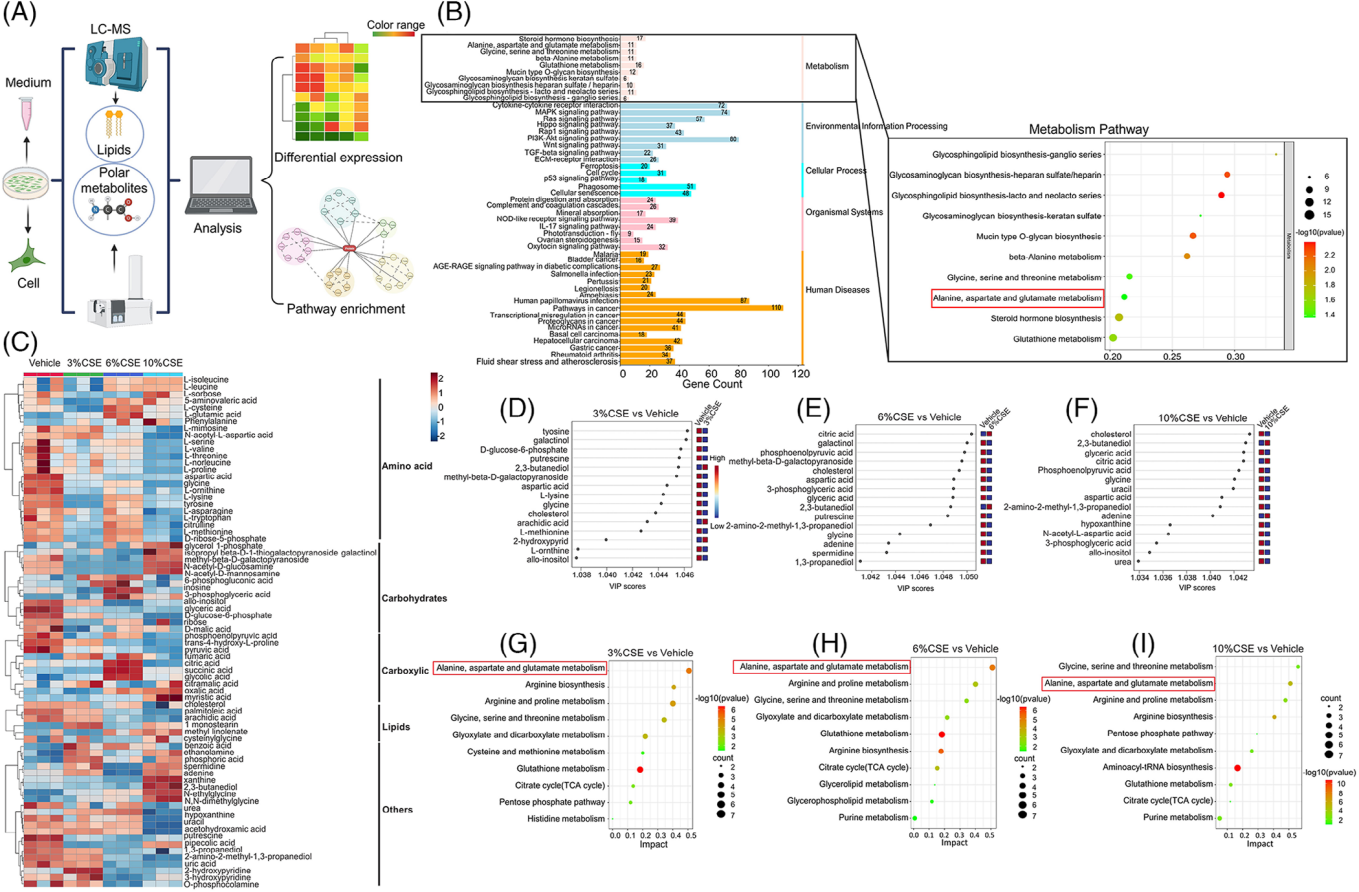
Cigarette smoking altered glutamate metabolism of airway epithelial cells. The workflow of metabolomic and lipidomic studies illustrates the measurement of intra‐ or extra‐epithelial lipidomics and metabolomics and the enrichment pathways (A). The pathway enrichment analysis employed KEGG analyses. We especially focused on pathways related to metabolism (B) by bulk RNA‐*seq* of human bronchial epithelial cells (HBE) after challenge with 10%CSE (*n* = 3/group). The metabolomic heatmap showed the changes of detected metabolites which were categorised by metabolite classes (C). Cells were stimulated with different concentrations of CSE for 24 h, and intracellular metabolites were measured (*n* = 3/group). The results of different concentrations of CSE (3%, 6%, and 10%) were compared with those of the 0% control group. The 15 highest variable importance projection (VIP) score metabolites were compared between 3% CSE and vehicle (D), 6% CSE and vehicle (E), and 10% CSE and vehicle (F). The 15 highest changed KEGG pathways were compared between 3% CSE and vehicle (G), 6% CSE and vehicle (H), and 10% CSE and vehicle (I). CSE, cigarette smoking extract.

To monitor the roles of CSE in reprogramming of metabolomic changes in airway epithelial cells, profiles of lipids and metabolites were analysed in HBE cells challenged with vehicle or CSE at 3%, 6%, and 10% for 24 h, respectively (Figure 2A). About 76 metabolites were identified, including 24 amino acids, 14 carbohydrates, 10 carboxylic acids, 6 lipids and 22 others. Of KEGG pathways, alanine, aspartate and glutamate metabolism pathways were highly enriched (Figure 2B). We furthermore investigated the corresponding gene expression profile of HBE after CSE (10%) stimulation using cell bulk RNA sequencing. After the gene enrichment, we selected the top 10 signalling pathways with significant differences and confirmed that alanine, aspartate and glutamate metabolism pathways were significantly enriched (Figure 2B).

The heat map in Figure 2C reveals that CSE induced changes in all metabolite classes, of which changes in amino acids were especially apparent. CSE at various concentrations significantly increased levels of 2,3‐butanediol, arachidic acid, and l‐glutamic acid, whilst reduced 1,3‐propanediol, 2‐amino‐2‐methyl‐1,3‐propanediol, 3‐phosphoglyceric acid, acetohydroxamic acid, allo‐inositol, aspartic acid, cholesterol, glyceric acid, glycine, l‐lysine, l‐ornithine, l‐valine, palmitoleic acid, phosphoenolpyruvic acid, putrescine, trans‐4‐hydroxy‐l‐proline, and tyrosine, as compared with vehicle at 24 h. To estimate the importance of each variable, we used the variable importance projection (VIP) score and found 15 top‐scoring metabolite VIPs more than 1 after CSE challenge at 3%, 6%, and 10% (*p *< .05; Figure 2D–2F, respectively), with the potential of biomarkers. The pathway enrichment analysis demonstrated that CSE altered metabolism pathways of alanine, aspartate and glutamate in HBE cells after CSE challenge at 3%, 6% and 10% (Figure 2G–I, respectively), different from those after vehicle. Detailed differences in intracellular and extracellular metabolites are shown in Tables S5–S10.

### 3.3 Regulatory roles of glutamic pyruvate transaminase 2 in metabolism reprogramming

**FIGURE 3:**
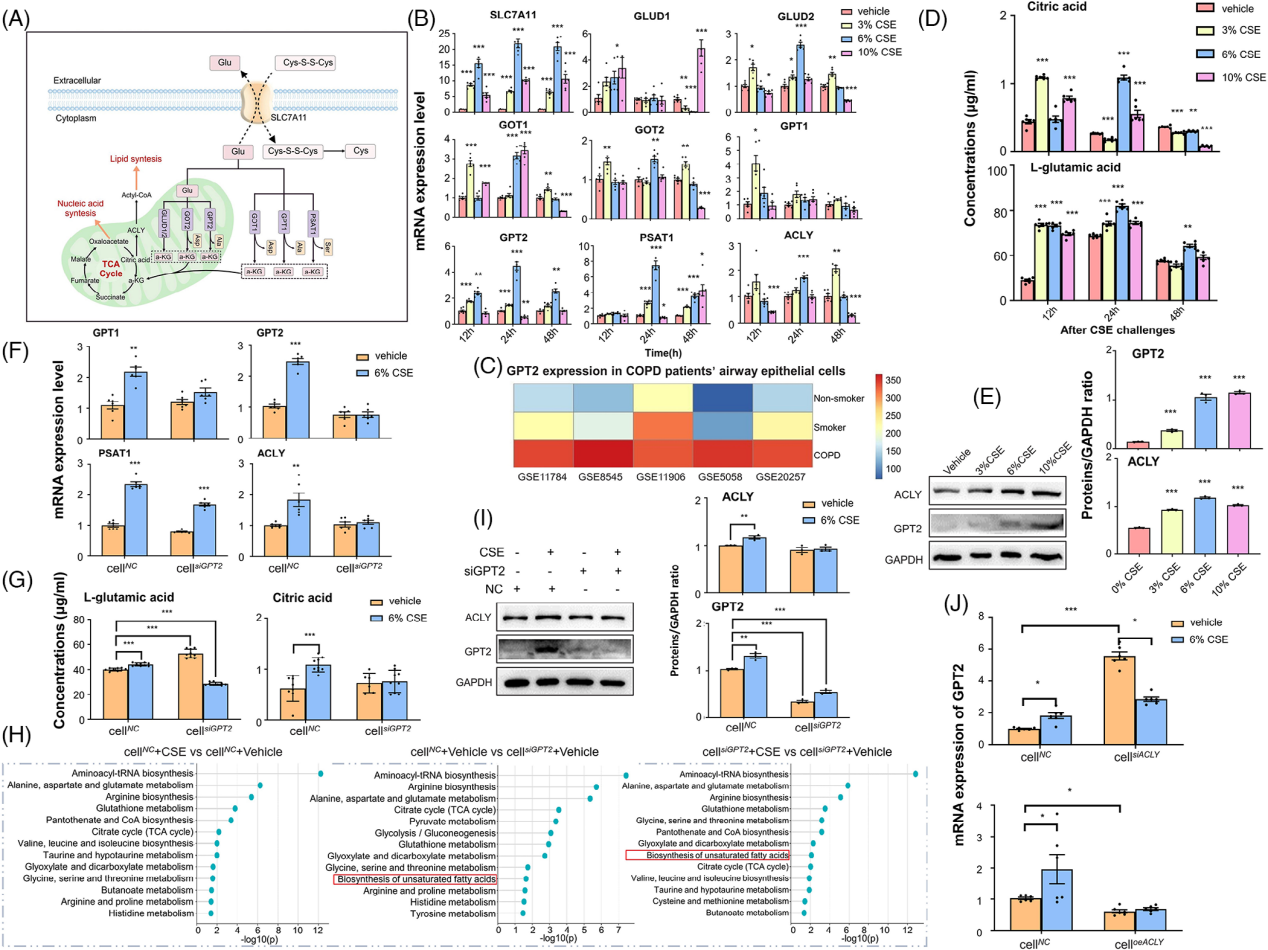
Regulatory roles of GPT2 in CSE‐altered glutamate metabolism genes. On the basis of glutamate metabolic pathway‐associated key metabolites, genes, proteins, and enzymes (A), CSE‐induced alterations of solute carrier family 7 member 11 (SLC7A11), glutamate dehydrogenase 1/2 (GLUD1/2), glutamic‐oxaloacetic transaminase 1/2 (GOT1/2), glutamic‐pyruvic transaminase 1/2 (GPT1/2), phosphoserine aminotransferase 1 (PSAT1), and ATP citrate lyase (ACLY) (*n* = 6/group) (B). Bioinformatics analyses demonstrated that GPT2 was significantly up‐regulated in airway epithelial cells of COPD smokers (C). CSE increased levels of citric acids and l‐glutamic acids HBE produced (*n* = 6/group) (D). CSE increased the protein expression of GPT2 and ACLY (*n* = 3/group) (E). Knockdown of GPT2 (siGPT2) inhibited CSE‐increased expression of GPT1, GPT2, and ACLY mRNA (*n* = 6/group) (F) and levels of intracellular l‐glutamic acids and citric acids (*n* = 6/group) (G). Metabolomics analyses demonstrated alterations of metabolite enrichment pathways between cells with non‐specific siRNA as cell*^NC^* challenged with CSE and cell*^NC^* with the vehicle, between cells with GPT2 siRNA (cell*^siGPT2^*) and cell*^NC^* with the vehicle, and between cell*^siGPT2^* with CSE and cell*^siGPT2^* with vehicle (*n* = 6/group) (H). The pathway enrichment analysis employed KEGG analyses. The cell*^siGPT2^* inhibited CSE‐increased protein levels of GPT2 and ACLY (*n* = 3/group) (I). The down‐ (cell*^sACLY2^*) or up‐regulation (cell*^oeACLY^*) of ACLY affected the expression of GPT2 (*n* = 6/group) (J). * and ** stand for the *p*‐values less than .05 and .01, as compared with the controls. CSE, cigarette smoking extract.

We mapped the metabolism pathways of alanine, aspartate, and glutamate and labelled key metabolites, genes, proteins, and enzymes involved in the potential mechanism of metabolite pathways. Citrate (citric acid) plays a crucial role in the synthesis of fatty acids by supplying acetyl‐CoA as the initial substrate. Glutamate enters the tricarboxylic acid (TCA) cycle, leading to the production of citric acid, actively participating in the process of fatty acid synthesis. ACLY catalyses the conversion of citric acid into fatty acids (Figure 3A). We examined the expression of genes encoding key enzymes in the pathway and found that the expression of involved genes varied amongst CSE concentrations and challenge times, for example, SLC7A11 up‐expressed from 12 h and on after CSE challenge, as compared with vehicle, of which the highest expression was noted after 6%CSE (Figure 3B). Of those, the expression of glutamate dehydrogenase 2 (GLUD2), glutamic oxaloacetic transaminase 1 (GOT1), glutamic oxaloacetic transaminase 2 (GOT2), GPT1, and GPT2 increased after 3% CSE at 12 h, whilst GLUD2, GOT1, GOT2, GPT2, phosphoserine aminotransferase 1 (PSAT1), and ACLY increased after 6% CSE at 24 h. The expression of those genes in airway epithelial cells demonstrated that GPT2 mRNA over‐expressed in epithelial cells of smokers with or without COPD (Figure 3C). Levels of citric acids increased 12 or 24 h after 3% and 10% CSE or after 6% and 10% CSE, whilst decreased 24 or 48 h after 3% CSE or after 6% and 10% CSE (Figure 3D). CSE elevated levels of l‐glutamic acids at 12 and 24 h (Figure 3D) and increased the protein expression of ACLY and GPT2 in a concentration‐dependent pattern (Figure 3E).

To further evaluate regulatory roles of GPT2 or ACLY in metabolism and expressions, we down‐regulated epithelial GPT2 and found that CSE failed to increase expression of GPT1, GPT2, and ACLY mRNA (Figure 3F) and intracellular levels of citric acids and l‐glutamic acids in cell*^siGPT2^* (Figure 3G). Of metabolomic profiles, the knockdown regulation of GPT2 mainly influenced the biosynthesis of unsaturated fatty acids and modulated the CSE‐activated pathway from glutamate metabolism to lipid synthesis, as shown in Figure 3H and Table S11. Of altered metabolic pathways, we selected glutamate metabolism as the target pathway on the basis of the glutamate connection between glutamate metabolism and lipid synthesis through ACLY‐catalysed citric acid. CSE increased the expression of GPT2 and ACLY proteins in cell*^NC^*, whilst did not in cell*^siGPT2^* (Figure 3I). The expression of GPT2 in cell*^siACLY^* was significantly higher than that in cell*^NC^* after vehicle treatment, whilst lower after the CSE challenge (Figure 3J). The up‐regulation of ACLY gene expression in cell*^oeACLY^* prevented CSE‐increased expression of GPT2 (Figure 3J), indicating the regulatory roles of the ACLY gene in epithelial GPT2 expression. The down‐regulation of GPT2 expression failed to affect the expression of other key enzymes in the glutamate metabolism pathway (Figure S1A–D).

### 3.4 Roles of glutamic pyruvate transaminase in lipidomic changes in airway epithelial cells

**FIGURE 4:**
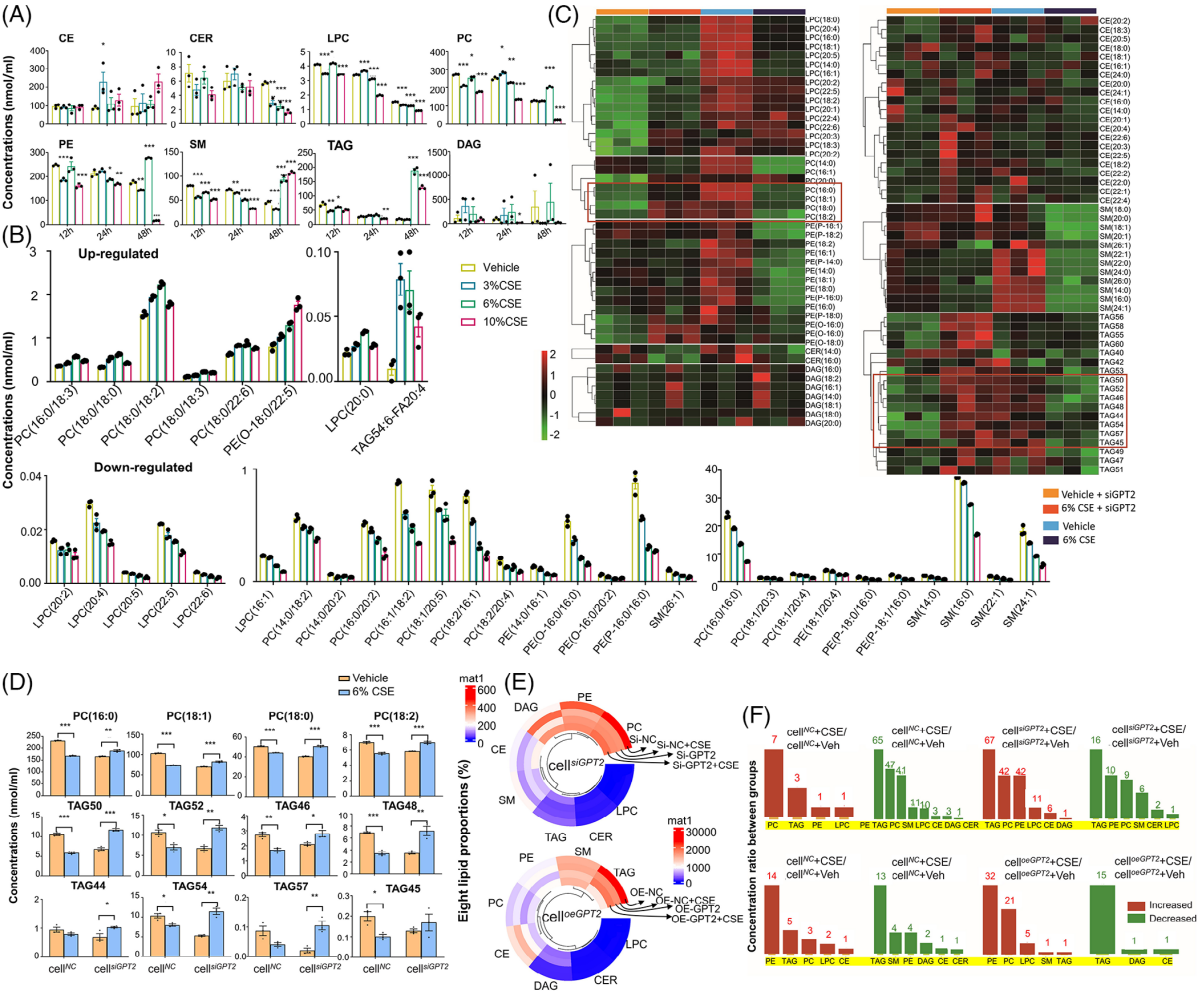
Regular roles of GPT2 in CSE‐induced lipidomic alterations. Levels of eight major lipid classes, for example, cholesterol ester (CE), ceramide (CER), lysophosphatidyl cholines (LPC), phosphatidyl cholines (PC), phosphatidyl ethanolamine (PE), sphingomyelin (SM), triacylglyceride (TAG), and diacylglycerol (DAG), were altered in HBEs challenged with vehicle or CSE at 3%, 6%, or 10% for 24 h (A). CSE upregulated or downregulated lipid elements based on the number of carbon atoms and unsaturated bonds, as compared with HBE challenged with vehicle (B). Lipidomic heat maps showed the difference of lipid elements between cell*^NC^* or cell*^siGPT2^* with vehicle or CSE (C). We shall found that cell*^NC^* or cell*^siGPT2^* showed different sensitivities of some PC or TAG elements in responses to CSE (D). Pie charts were used to outline the proportion (%) of the eight classes of lipids (E). The increased or decreased number of different lipid classes was summarised by assessing the concentration ratio between cell*^NC^* or cell*^siGPT2^* with vehicle or CSE (F). Each group contains three samples. * and ** stand for the *p*‐values less than .05 and .01, as compared with the controls. CSE, cigarette smoking extract.

We examined eight major lipid classes in intra‐ and extra‐epithelia cells, including LPC, PC, PE, DAG, SM, CE, CER, and TAG, 12, 24 and 48 h after challenges with vehicle or CSE at 3%, 6% and 10%, as were detailed in Tables S12–S17. Levels of LPC, PC, PE and SM in HBE cells were significantly lower from 12 h and on after CSE at 3% and 10%, whilst CE was higher at 24 h after 3% CSE, LPC at 12 and 24 h, PC at 24 and 48 h, and PE, SM, TAG at 48 h after 6% CSE, as compared with those after vehicle (Figure 4A). Levels of LPC (20:0), PC (16:0/18:3, 18:0/18:0, 18:0/18:2, 18:0/18:3, 18:0/22:5), PE (O‐18:0/22:5) and TAG (54:6/FA20:4) elevated, whilst LPC (16:1), PC (14:0/18:2, 16:0/16:0, 16:0/20:2, 16:1/18:2, 18:2/16:1, 18:2/20:4), PE (14:0/16:1, 16:0/16:0) and SM (16:0, 24:1) reduced 24 h after CSE challenges (Figure 4B). CSE‐induced lipidomic alterations appeared in a concentration‐dependent pattern.

To uncover regulatory roles of GPT2 in lipidomic changes, we evaluated the effects of CSE on lipids metabolism in cell*^NC^* or in cell*^siGPT2^* and profiled changes in lipid elements based on the number of carbon atoms and unsaturated bonds. Levels of PC (16:0, 18:0, 18:1, 18:2) and TAGs (44, 45, 46, 48, 50, 52, 54, 57) in cell*^siGPT2^* challenged with CSE were similar with cell*^NC^* with vehicle (Figure 4C). It indicates that GPT2 and associated metabolites play critical roles in the regulation of PC and TAG metabolisms. Levels of those lipid elements significantly increased in cell*^siGPT2^* after the CSE challenge, different from those in cell*^NC^* (*p* < .05 or less, respectively, Figure 4D). To investigate the roles of GPT2 in lipid metabolisms, lipidomic profiles of cell*^siGPT2^* and cell*^oeGPT2^* were measured after challenges with CSE or vehicle and compared between cells with CSE and vehicle and between cell*^siGPT2^* and cell*^oeGPT2^*. There was an obvious difference in proportions (%) of lipids in eight classes and contribution ratios between cell*^siGPT2^* and cell*^oeGPT2^* (Figure 4E,F). Of ratios between CSE and vehicle, PC and TAG or PE and PC increased in cell*^siGPT2^* or cell*^oeGPT2^*, respectively, whilst TAG and PE or TAG reduced in cell*^siGPT2^* and cell*^oeGPT2^*. Details of comparisons and values are listed in Tables S18 and S19. It implies that dynamic altercations and reprogramming of lipid metabolism and metabolites are highly dependent upon the existence of GPT2, whilst GPT2 expression was associated with the severity and duration of CSE challenges.

### 3.5 Glutamic pyruvate transaminase 2‐oriented transcriptomic profiles and regulations

**FIGURE 5:**
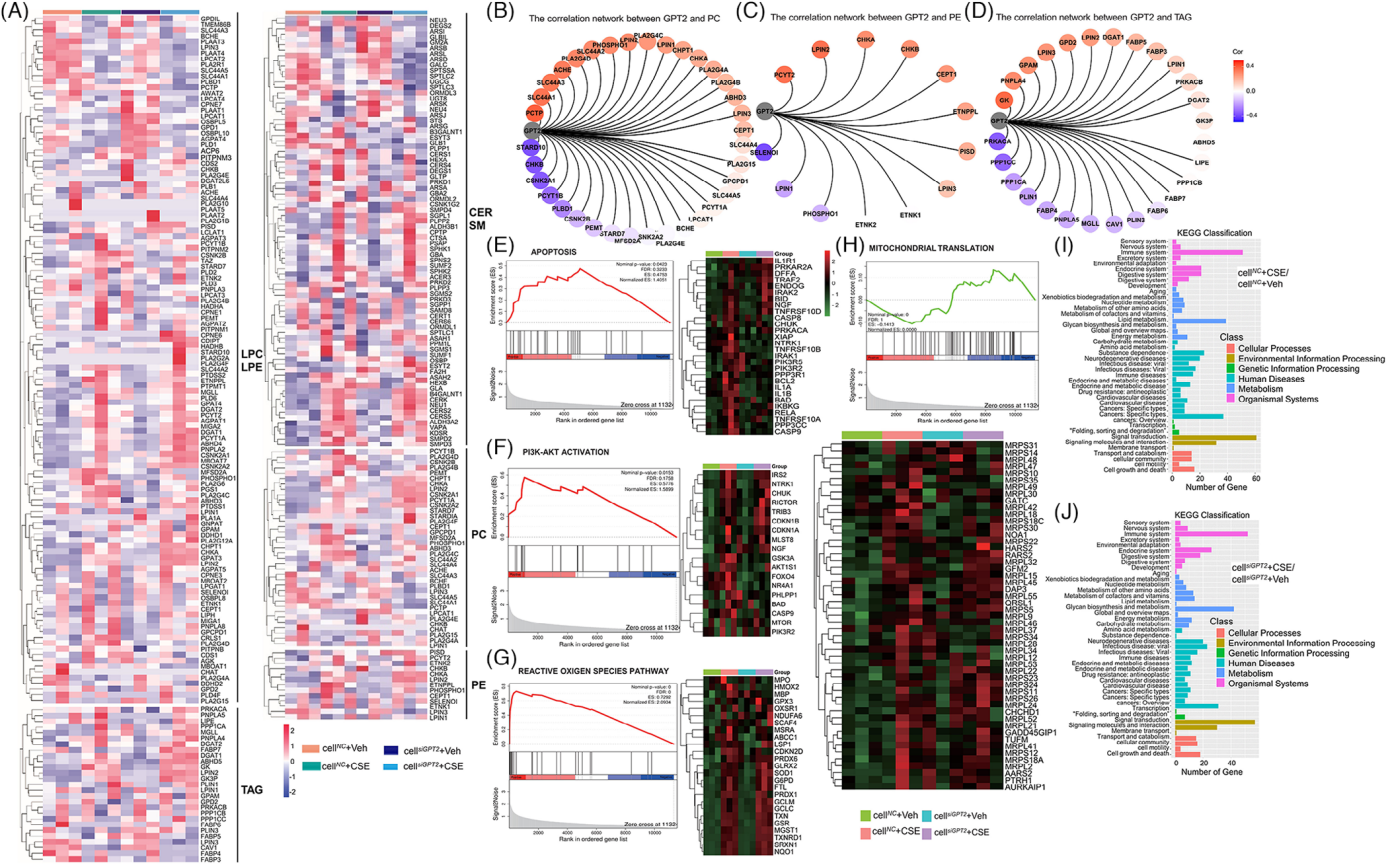
Regulatory roles of GPT2 in CSE‐induced alterations of lipid metabolism‐associated transcriptomic profiles. Lipid metabolism‐associated transcriptomic profiles of cells with non‐specific siRNA (cell*^NC^*) or cells with GPT2 siRNA (cell*^siGPT2^*) challenged with 6% CSE or vehicle were measured by bulk RNA sequencing (*n* = 3/group) and presented according to different classes of lipids (A). We defined the correlation networks of GPT2 with PC metabolism‐associated genes using Pearson's correlation method (B), PE metabolism‐associated genes (C), or TAG metabolism‐associated genes (D). We employed the KEGG, Gene Ontology (GO), Hallmark, and Reactome databases/resources for pathway enrichment analysis of differentially expressed genes. The transcriptomic profiles associated with apoptosis (E), PI3K‐AKT activation (F), reactive oxygen species pathway (G), or mitochondrial translation pathway (H) were further assessed. Metabolomics demonstrates KEGG classification between cell*^NC^* with the vehicle and CSE (I) and between cell*^siGPT2^* with the vehicle and CSE (J). Each group contains three samples. CSE, cigarette smoking extract.

To elucidate potential mechanisms by which GPT2 alters cell metabolism and function, we assessed transcriptomic expressions and regulations in cell*^NC^* and cell*^siGPT2^* 24 h after 6% CSE challenge. The details of DEGs between cell*^NC^* with vehicle and CSE, between cell*^siGPT2^* with vehicle and CSE, as well as between cell*^siGPT2^* and cell*^NC^* with vehicle or CSE, respectively, are listed in Table S20. We focused on lipid metabolisms‐associated gene transcriptomic profiles and found a clear correlation between GPT2 and genes related to TAG, PC, and PE metabolisms (Figure 5A). GPT2 had highly positive correlations with phosphatidylcholine transfer protein (PCTP), solute carrier family 44 member 1 (SLC44A1), solute carrier family 44 member 3 (SLC44A3), acetylcholinesterase (ACHE), and phospholipase A2 group IVD (PLA2G4D), and negative correlations with StAR related lipid transfer domain containing 10 (STARD10), choline kinase beta (CHKB), and casein kinase 2 alpha 1 (CSNK2A1) in PC metabolism (Figure 5B). GPT2 had highly positive correlations with phosphatidylcholine CTP‐transferase 2 (PCYT2), lipin 2 (LPIN2), choline kinase alpha (CHKA), and choline kinase beta (CHKB), and negative correlations with selenoprotein I (SELENOI) in PE metabolism, respectively (Figure 5C). GPT2 had highly positive correlations with glycerol kinase (GK), patatin like phospholipase domain containing 4 (PNPLA4), glycerol‐3‐phosphate acyltransferase, mitochondrial (GPAM), and lipin 3 (LPIN3), and negative correlations with protein kinase cAMP‐activated catalytic subunit alpha (PRKACA) and protein phosphatase 1 catalytic subunit gamma (PPP1CC) in TAG metabolism (Figure 5D). Enriched pathways in apoptosis (Figure 5E), PI3K‐AKT activation (Figure 5F), ROS reaction (Figure 5G), and mitochondrial translation (Figure 5H) were significantly different between cell*^siGPT2^* with CSE and vehicles. The higher radio of related gene expressions between cell*^NC^* with CSE and vehicle (Figure 5I) than that between cell*^siGPT2^* with CSE and vehicle (Figure 5J) indicates altered pathways representing molecular interaction, reaction, and relation networks within cell functions, as detailed in Figure S2A–D.

### 3.6 Roles of glutamic pyruvate transaminase 2 in cigarette smoking extract ‐altered mitochondrial function and cell apoptosis

**FIGURE 6:**
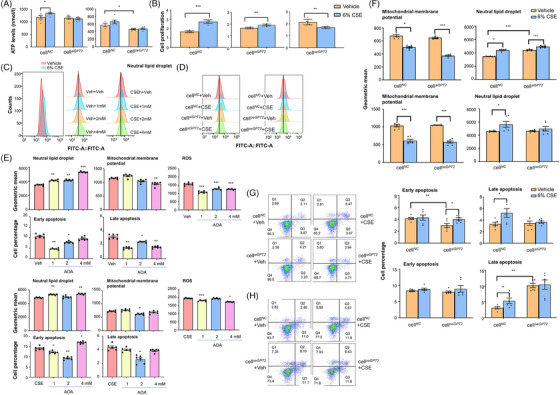
Regulatory roles of GPT2 in cell lipid and energy homeostasis, mitochondrial function, and survival. Roles of GPT2 in airway epithelial lipid and energy homeostasis and survival were investigated by detecting ATP levels (A), proliferation (B), or lipid droplets (LDs) of cell*^NC^*, cell*^siGPT2^*, or cell*^oeGPT2^* 24 h after challenges with vehicle or 6% CSE. CSE induced an increase of the intracellular LD contents increased cell*^NC^* pretreated with GPT2 inhibitor aminooxyacetate (AOA) (C) or cell*^siGPT2^* (D), but not in cell*^oeGPT2^* (D). The dose‐dependent effects of AOA on mitochondrial membrane potentials, lipid droplets, reactive oxygen species (ROS), or early or late apoptosis were further assessed in HBE treated vehicle or CSE (E). Levels of mitochondrial membrane potentials and lipid droplets (F) or early and late cell apoptosis (G,H) were assayed in cell*^NC^*, cell*^siGPT2^*, or cell*^oeGPT2^* challenged with vehicle or CSE. Each group contains six samples. * and ** stand for the *p*‐values less than .05 and .01, as compared with the controls. CSE, cigarette smoking extract.

Levels of the intracellular ATP production significantly increased in cell*^NC^* 24 h after CSE challenge, rather than in cell*^siGPT2^* (Figure 6A). Levels of ATP in cell*^oeGPT2^* were lower than those in cell*^NC^* or cell*^siGPT2^*, whilst there was no significant difference of cell*^oeGPT2^* or cell*^siGPT2^* between vehicle and CSE challenges. It indicates that GPT2 gene modulations may reduce the sensitivity of the cellular ATP production against CSE challenge. CSE at 6% significantly increased the proliferation of cell*^NC^* or cell*^siGPT2^*, and reduced cell*^oeGPT2^* proliferation (Figure 6B). Levels of mitochondrial membrane potentials (MMPs) and neutral lipid droplets (LDs) were measured with mito‐tracker red and BODIPY493/503 in cell*^NC^*, cell*^siGPT2^*, or cell*^oeGPT2^* 24 h after challenges with vehicle or 6% CSE, respectively (Figures 6C‐6F). AOA at 4 mM significantly reduced levels of MMPs and early and late apoptosis or ROS from 1 mM and on, whilst LDs increased in cell*^NC^*, as compared with those treated with vehicle (Figure 6E). In HBE cells challenged with CSE, levels of LDs increased at 1 and 4 mM of AOA, whilst early and late apoptosis decreased at 2 mM or ROS at 1 and 4 mM, as compared with CSE‐cells treated with vehicle (Figure 6E). CSE significantly reduced levels of MMPs in cell*^siGPT2^* and cell*^oeGPT2^* and elevated levels of LDs in cell*^siGPT2^*, as compared with the corresponding cells challenged with vehicle (Figure 6F). Levels of early apoptosis in cell*^siGPT2^* were lower than those in cell*^NC^* and increased significantly after the CSE challenge (Figure 6G), whilst levels of late apoptosis in cell*^oeGPT2^* were significantly higher than those in cell*^NC^* (Figure 6H). Our data indicate that GPT2 gene modification may change epithelial cell sensitivity in response to CSE.

### 3.7 Cigarette smoking extract roles in interactions amongst lipid metabolism‐associated genes

**FIGURE 7:**
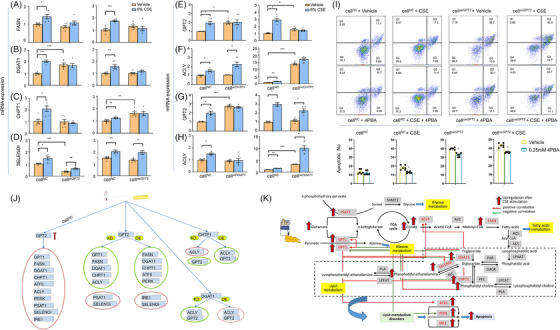
Interactions between GPT2 and associated genes as well as metabolism reprogramming. Regulatory roles of GPT2 in mRNA expression of fatty acid synthase (FASN) (A), diacylglycerol O‐acyltransferase 1(DGAT1) (B), choline phosphotransferase 1(CHPT1) (C), or selenoprotein I (SELENOI) (D) were assayed in cell*^NC^*, cell*^siGPT2^*, or cell*^oeGPT2^* challenged with vehicle or CSE. Regulatory roles of CHPT1 (E,F) or DGAT1 (G and H) in mRNA expression of GPT2 or ACYL were assessed in cell*^NC^*, cell*^siCHTP1^*, or cell*^oeCHTP1^* or in cell*^NC^*, cell*^siDGAT1^*, or cell*^oeDGAT1^* 24 h after challenges with vehicle or 6%CSE. Roles of endoplasmic reticulum (ER) stress in late apoptosis were evaluated in cell*^NC^* or cell*^oeGPT2^* pretreated with the ER stress inhibitor 4‐phenylbutyric acid (4‐PBA) or vehicle and challenged with CSE or vehicle (I). The interactions and inter‐gene regulations between glutamate metabolism‐associated genes in responses to CSE are summarised (J), to headline the up‐regulated genes in red circle or down‐regulated genes in blue circle under the conditions of target gene knockdown (KD) or over‐expressed (OE) in the yellow. Based on alternations and up/down‐regulations of those metabolism target genes, the reprogramming maps of corresponding metabolites and metabolic pathways are summarised for the further understanding (K). Each group contains six samples. * and ** stand for the *p*‐values less than .05 and.01, as compared with the controls. CSE, cigarette smoking extract.

We further investigated whether GPT2 influences the expression of other lipid metabolism‐associated genes and the sensitivity of epithelial cells in response to CSE. We selected fatty acid synthase gene (FASN, Figure 7A) for converting carbohydrates to fatty acids, DGAT1 (Figure 7B) for DAG and fatty acyl CoA to TAG, CHPT1 (Figure 7C) for phosphatidylcholine biosynthesis, or SELENOI (Figure 7D) for CDP‐ethanolamine to DAG. The expression of DGAT1 significantly increased and SELENOI decreased in cell*^siGPT2^* or CHPT1 increased in cell*^oeGPT2^*, as compared with cell*^NC^*. CSE failed to increase the expression of FASN, DGAT1, CHPT1, glutamate dehydrogenase 1 (GLUD1), GLUD2, GPT1, protein kinase R (PKR)‐like endoplasmic reticulum kinase (PERK), or activating transcription factor 6 (ATF6) (Figure 7A–D, Figure S1C,D, Figure S3F, and Figure S3A–F) in cell*^siGPT2^* or cell*^oeGPT2^*, different from changes in cell*^NC^*, whilst CSE still increased the expression of SELENOI (Figure 7D), PSAT1 (Figure 3F), or inositol‐requiring enzyme 1 (IRE1) (Figure S3B–E).

We also assessed potential influences of CHTP1 and DGAT1 in GPT2 expression and found that levels of GPT2 (Figure 7E–G) or ACLY expression (Figure 7F–H) were significantly higher in cell*^siCHTP1^* and cell*^oeCHTP1^* than in cell*^NC^*, respectively, as compared with cell*^NC^* with vehicle or CSE, or with cell*^siCHTP1^* or cell*^oeDGAT1^* with vehicle, respectively (*p *< .05 or less). CSE failed to increase the expression of GPT2 in cell*^siCHTP1^* and cell*^oeCHTP1^* (Figure 7E–G) or in cell*^siDGAT1^* (Figure 7G), and ACLY in cell*^oeCHTP1^* (Figure 7F) or cell*^siDGAT1^* (Figure 7H), respectively, as compared with the corresponding cells with vehicle. To verify roles of ER stress in GPT2‐associated cell late apoptosis and response to CSE, we pretreated cell*^NC^* or cell*^oeGPT2^* with the ER stress inhibitor 4‐phenylbutyric acid (4‐PBA) and found that 4‐PBA deceased the cell apoptotic number in cell*^NC^* after CSE challenge, whilst in cell*^oeGPT2^* after vehicle or CSE (Figure 7I). It indicates that GPT2 overexpression increased the sensitivity of airway epithelia to inhibition of ER stress. CSE‐induced alterations of GPT2‐ and associated genes‐, and CHTP1‐oriented expression profiles, corresponding metabolites, and pathways are summarised in Figure 7J,K, respectively.

### 3.8 Interactions between target genes and PI3K signalling pathway

**FIGURE 8:**
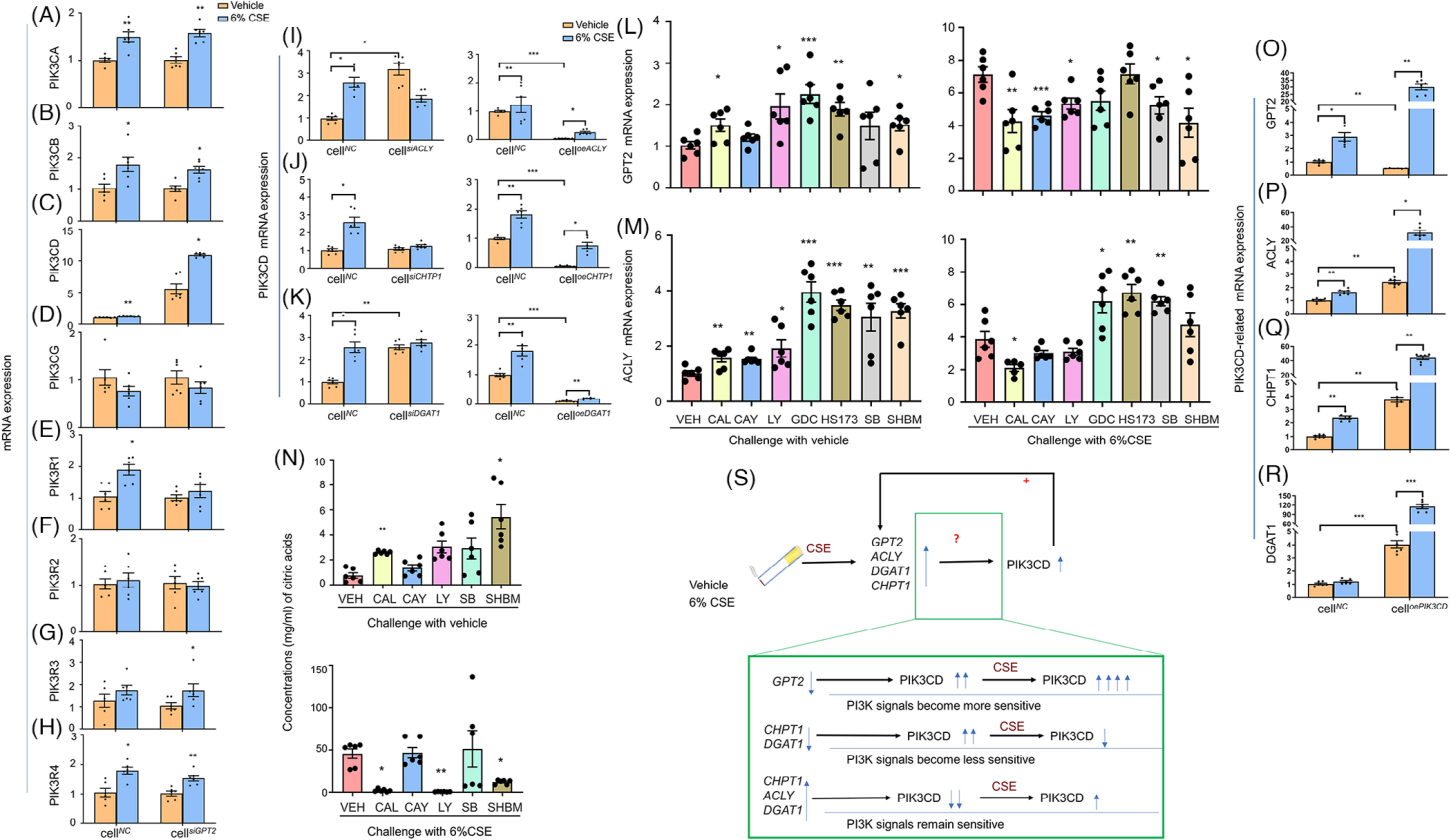
CSE affected the interactions and regulations between GPT2 and PI3K signalling pathways. Regulatory roles of GOT2 in mRNA expression of PI3K subset genes were assessed in cell*^NC^* or cell*^oeGPT2^* after CSE or vehicle challenges, including PIK3CA (A), PIK3CB (B), PIK3CD (C), PIK3CG (D), PIK3R1 (E), PIK3R2 (F), PIK3R3 (G), and PI3KR4 (H). Regulatory roles of ACLY (I), CHPT1 (J), or DGAT1 (K) in mRNA expression of PI3KCD were evaluated in cell*^NC^*, cell*^siACLY^*, or cell*^oeACLY^*, inn cell*^NC^*, cell*^siCHTP1^*, or cell*^oeCHTP1^*, or in cell*^NC^*, cell*^siDGAT1^*, or cell*^oeDGAT1^* 24 h after vehicle or 6% CSE challenges. Regulatory roles of KI3K subsets in mRNA expression of GPT2 (L) or ACLY (M) were explored using selected inhibitors of PI3K subsets, including CAL‐101 (targeting p110δ), CAY10505 (targeting PI3Kγ), LY294002 (targeting PI3Kα/δ/β), GDC0941 (targeting PI3Kα/δ), HS173 (targeting PI3Kα), SB216763 (targeting GSK‐3α), or SHBM1009 (targeting p110α/γ/δ/β). The inhibition of PI3K subsets (e.g., CAL, CAY, LY, SB, or SHBM) reduced CSE‐increased intracellular levels of citric acids in HBEs (N). Regulatory roles of PI3KCD in mRNA expression of GPT2 (O), ACLY (P), CHPT1 (Q), or DGAT1 (R) were assessed in cell*^NC^* or cell*^oePI3KCD^* after vehicle or CSE challenges. The interactions and regulations between metabolism‐associated genes and PI3K subsets were summarised (S), where we can see that the PI3KCD became less sensitive to CSE when CHPT1 or DGAT1 were down‐regulated, whilst more sensitive when GPT2 gene was down‐regulated or CHPT1, ACLY, or DGAT1 were up‐regulated. Each group contains six samples. * and ** stand for the *p*‐values less than .05 and .01, as compared with the controls. CSE, cigarette smoking extract.

To define roles of GPT2, ACLY, CHPT1, or DGAT1 in the PI3K signal pathway, we measured the expression of PI3K subset genes in conditions of the target metabolism gene up/down‐regulations (Figure 8A–H). CSE (6%) for 24 h increased the expression of phosphatidylinositol‐4,5‐bisphosphate 3‐kinase catalytic subunit alpha, beta, and delta (PIK3CA, Figure 8A; PIK3CB, Figure 8B; and PIK3CD, Figure 8C), or phosphoinositide‐3‐kinase regulatory subunit 1 and 4 (PIK3R1, Figure 8E; PIK3R4, Figure 8H) in cell*^NC^*, whilst PIK3CD expression significantly elevated in cell*^siGPT2^* as compared with in cell*^NC^* and even more increased in cell*^siGPT2^* after CSE challenge as compared with after vehicle challenge (Figure 8C). There was no significant difference of phosphatidylinositol‐4,5‐bisphosphate 3‐kinase catalytic subunit gamma (PIK3CG, Figure 8D) and phosphoinositide‐3‐kinase regulatory subunit 2 (PIK3R2, Figure 8F). The expression of PIK3CD was significantly higher in cell*^siACLY^* (Figure 8I) and cell*^oeDGAT1^* (Figure 8K) and lower in cell*^oeACLY^* (Figure 8I), cell*^oeCHTP1^* (Figure 8J), and cell*^oeDGAT1^* (Figure 8K), as compared with cell*^NC^*, whilst decreased in cell*^siACLY^* and increased in cell*^oeACLY^*, cell*^oeCHTP1^*, and cell*^oeDGAT1^* after CSE challenge, as compared with those cells challenged with vehicle, respectively (*p *< .05 or less).

We investigated effects of various PI3K subsets in metabolism‐associated genes and metabolites, using selective inhibitors of PI3K. The expression of GPT2 (Figure 8L) increased about one‐fold after HBE cells were treated with LY (LY294002), GDC (GDC0942) or HS173, and ACLY mRNA (Figure 8M) about 3−4 folds after cells were treated with GDC, HS173, SB (SB216723), or SHBM (SHBM1009), as compared with HBE cells treated with vehicle. The expression of GPT2 or ACLY in PI3K inhibitors‐treated cells after CSE was significantly higher than those after vehicle. When compared amongst cells challenged with CSE, GPT2 expression was significantly lower in cells treated with CAL (CAL‐101), CAY (CAY10505), LY, SB, or SHBM (Figure 8L), whilst ACLY expression was lower in cells with CAL, and higher in cells with GDC, HS173, or SB (Figure 8M). The concentrations of citric acids in cells with vehicle were significantly higher than those with CAL, LY and SHBM after CSE challenge (Figure 8N). GPT2 expression in cell*^oePI3KCD^* was not different from that in cell*^NC^*, whilst increased significantly after CSE (Figure 8O). The expression of ACLY (Figure 8P), CHTP1 (Figure 8Q) or DGAT1 mRNA (Figure 8R) in cell*^oePI3KCD^* was significantly higher than that in cell*^NC^* and became even higher after CSE challenge. Although CSE induced the over‐expression of metabolism‐associated genes and PI3K subset genes, the interactions between those two group genes varied amongst functional changes of metabolism genes. The PIK3CD became less sensitive to CSE when CHPT1 and DGAT1 were down‐regulated, and more sensitive when GPT2 gene was down‐regulated or CHPT1, ACLY, and DGAT1 were up‐regulated (Figure 8S). Those metabolism‐associated genes became more sensitive to CSE when PIK3CD was up‐regulated.

### 3.9 Glutamate metabolism as an alternative therapeutic target in chronic obstructive pulmonary disease

**FIGURE 9:**
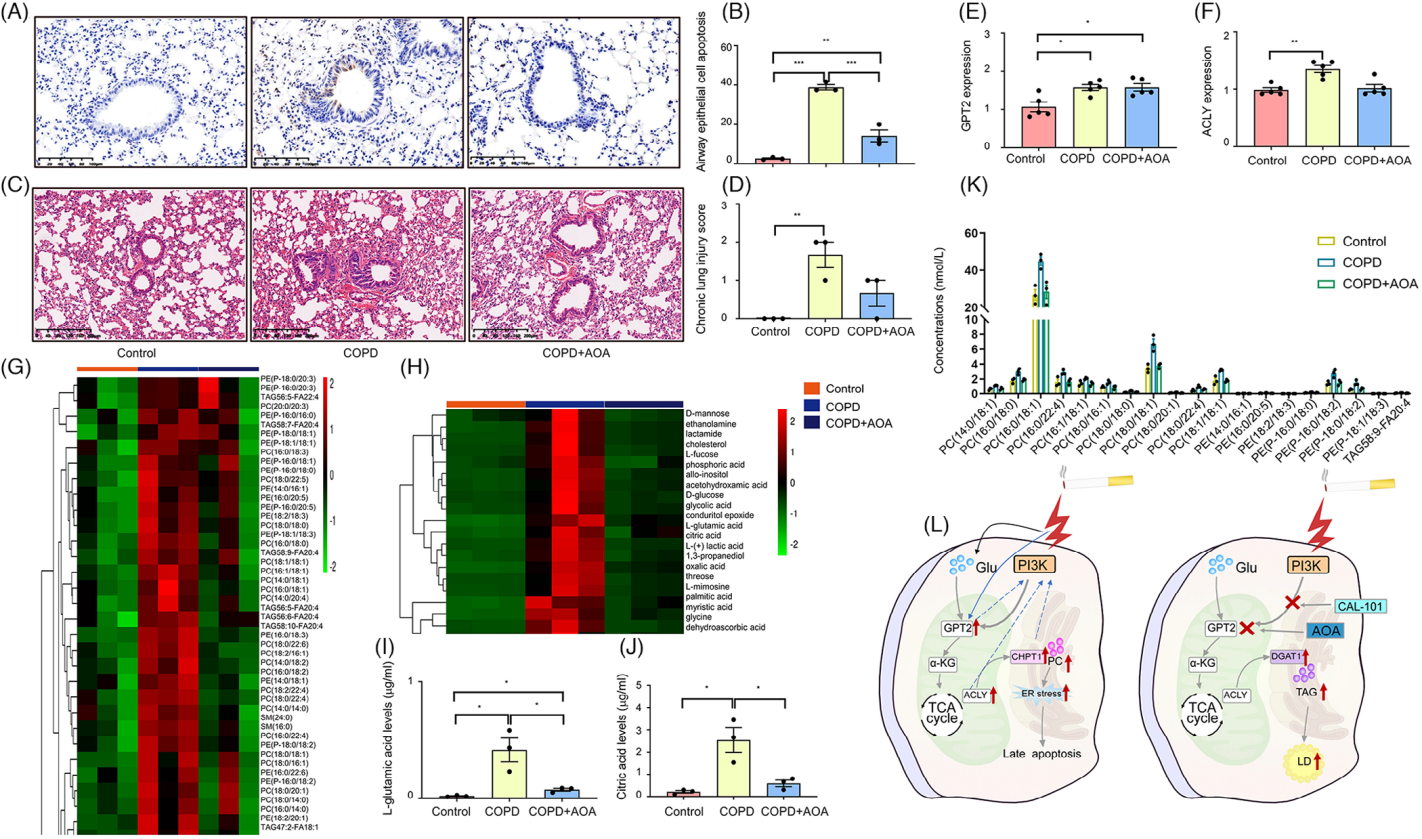
GPT2 inhibitor alleviates cigarette smoking‐induced lung injury and metabolic abnormality. The 15 wild‐type C57BL/6J mice were randomly divided into three groups, including animals treated with PBS or vehicle or AOA at 10 mg/kg/day for 5 days per week for 6 weeks and challenged with CSE at 30 μL/day for 5 days per week for 6 weeks. (A–D) demonstrated airway epithelial hyperplasia, hyperproduction of mucus, apoptosis, or lung tissue inflammation and injury (*n* = 3/group). The expression of lung tissue GPT2 (E) or ACLY (F) mRNA (*n* = 5/group) and the lung tissue profiles of lipidomics (*n* = 3/group) (G) or metabolomics (*n* = 3/group) (H) were measured 6 weeks after smoking provocations and treated with vehicle or AOA. The treatment with AOA significantly reduced lung tissue levels of l‐glutamic acids (I) and citric acids (J) in mice with chronic smoking. Lipidomic analyses illustrated that AOA treatment improved chronic smoking‐increased lipid elements based on the number of carbon atoms and unsaturated bonds (K). Our data present an alternative that the efficient inhibitions of glutamate metabolism and involved signalling pathways can improve airway epithelial origin metabolisms and lung injury (L).

To evaluate the impact of GPT2‐dominated glutamate metabolism in therapy for chronic lung inflammation, we selected a transaminase inhibitor AOA to target glutamate metabolism and reduce glutamate conversion to α‐KG and assessed the therapeutic effects of AOA in the preclinical model of COPD. AOA is a pan‐amino acid transferase inhibitor and directly target the GPT2, evidenced by that AOA inhibited tumour growth though GPT2 knockdown, rather than tumour per se.^27^, ^28^ Chronic exposure to CSE significantly increased apoptotic numbers of airway epithelial cells in mice treated with vehicle, which was reduced by AOA (Figure 9A,B). CSE induced the appearance of epithelial apoptosis, epithelial remodelling, and inflammation mainly in small airways (Figure 9C). The AOA treatment attenuated CSE‐induced airway and alveolar inflammation, tissue damage, leukocyte infiltration, and chronic lung injury score (Figure 9D). The expression of GPT2 was elevated in the lung tissue of animals with COPD treated with vehicle or AOA (Figure 9E), whilst there was no statistical difference in ACLY expression between controls and COPD animals with AOA (Figure 9F). Details of lung lipidomic changes and comparisons amongst groups are listed in Table S21, of which 23 PC, 17 PE, 2 SM and 6 TAG presented significant differences between controls and COPD with vehicle and between COPD with vehicle or AOA (Figure 9G). Our data demonstrated that inhibition of the GPT2 function reduced CSE‐induced PC synthesis. Details of lung metabolomic profiles and comparisons are listed in Table S22, of which some metabolites in lung tissues from COPD animals were most significantly altered and improved by the AOA treatment (Figure 9H). Of those metabolites, levels of glutamate (Figure 9I) and citric acid (Figure 9J) were significantly higher in COPD animals with vehicle, as compared with controls or COPD animals with AOA, respectively (*p *< .05). Levels of those lipid elements, especially PC (14:0/18:0, 16:0/18:0, 16:0/18:1, 16:0/22:4, 16:1/18:1, 18:0/16:1, 18:0/18:0, 18:0/20:1,18:0/22:4, 18:1/18:1), PE (14:016:1, 16:0/20:5, 18:2/18:3, P‐16:0/18:0, P‐16:0/18:2, P‐18:0/18:2, P‐18:1/18:3), and TAG (58:9‐FA20:4) (Figure 9K), significantly increased in COPD animals, whilst alleviated by the AOA treatment. The regulation of glutamate metabolism changed lung epithelial metabolism and survivals (Figure 9L), reshaped lung tissue microenvironmental metabolism, and improved CSE‐induced chronic lung injury.

## 4 DISCUSSION

This study screened and selected COPD‐targeting metabolism pathways and core regulatory molecules in airway epithelial cells. It discovered that GPT2 and its associated genes play a role in regulating the metabolic reprogramming and smoking induced the airway epithelial cell damage, primarily through their impact on lipid synthesis. This study offers the evidence that GPT2 contributes to the lipid metabolic reprogramming of airway epithelium following smoking, presenting a novel therapeutic approach for managing chronic lung diseases.

COPD is a highly heterogeneous disease characterised by systemic and local metabolic alterations compared to other lung diseases.^16^, ^24^, ^29^, ^30^ Metabolomics and lipidomics are increasingly employed to investigate the pathogenesis of COPD, unravel the complexity of chronic lung diseases, and discover disease‐specific biomarkers of prognosis and subtyping COPD patients.^31^, ^32^, ^33^ Metabolic disorders were correlated with the severity of lung dysfunction in acute exacerbation of COPD patients, of which perturbations of alanine, aspartate, and glutamate in airway epithelial cells were modelled as the critical pathway in experimental chronic lung inflammation and injury.^34^ Our data showed smoking per se altered the metabolic pathways of alanine, aspartate, and glutamate in airway epithelial cells. Of various amino acids changed in COPD patients, levels of glutamate were correlated with emphysema in COPD patients.^35^ Regulating glutamate levels effectively improved the severity of experimentally chronic lung inflammation.^36^ Together, this study selected the glutamate metabolism as the target pathway to further investigate molecular regulation of metabolism reprogramming in lung epithelial cells during chronic inflammation.

Altered lipids in plasma, including phospholipids, sphingolipids, glycerides, and cholesteryl esters, were suggested for early diagnosis, monitoring, and prognosis of acute exacerbation of COPD or stable COPD.^29^, ^37^, ^38^ The combination of lipidomics with clinical phenomics enables to determine disease severity, disease course, staging, subtype treatment and prognosis.^23^, ^39^, ^40^, ^41^ This study integrated circulating metabolomics and lipidomics and found that elevated levels of glutamate and glutamate‐associated pathways were accompanied by obvious alterations of lipid element levels in COPD patients. Cigarette smoking is the dominant factor in the pathogenesis of COPD,^42^ associated with cellular lipid metabolism, leading to the accumulation of the cytotoxic lipid CER in lung epithelial cells.^37^, ^43^ We examined changes in metabolic and lipidomic profiles in CSE‐stimulated airway epithelial cells and found activation of glutamate and glutamate‐associated pathways and regulatory roles of GPT2 in smoking‐induced metabolism reprogramming. This suggests that GPT2 plays a decisive regulation of lipid synthesis, to promote late apoptosis and reprogram the glutamate‐oriented metabolism of airway epithelial cells during the inflammation.

Our data demonstrated that CSE induced alternations of a large number of metabolism‐associated/specific gene expressions, especially glutamate metabolism‐associated/specific genes. The detailed information on gene abbreviations, full names, and genetic locations of these genes was listed in Table S23, including SLC7A11 for transport between cysteine and glutamate, GLUD1 and GLUD2 for deamination from glutamate to a‐KG, GOT1, and GOT2 for amino acid metabolism and the urea and tricarboxylic acid cycles, GPT1 (ALT1) and GPT2 (ALT2) for transamination between glutamate/alanine/oxoglutarate and pyruvate/a‐KG, PSAT1 for transamination from pyruvate to serine, and ACLY for synthesis of cytosolic acetyl‐CoA and oxaloacetate from citric acid and CoA. Those selected genes coding proteins play central roles in the regulation of amino acids, glucose, and lipids responsible for intracellular and intramitochondrial energy production (Figure S4). Of those selected metabolism genes, GPT1 and GPT2 regulate reversible transamination between glutamate/alanine and α‐KG, glutamate/alanine and pyruvate, as well as α‐KG and pyruvate, as a metabolism triangle centre to contribute to TCA cycle, citric acid cycle, and lipogenesis, as headlined in Figure S4. Pyruvate is transferred into the mitochondrial matrix through the mitochondrial pyruvate carrier in the inner mitochondrial membrane for mitochondrial pyruvate metabolism.^44^ We confirmed high expression of GPT2 in airway epithelial cells of COPD patients, obviously different from healthy and smokers. The down‐regulation of epithelial GPT2 reduced the sensitivity of GPT1, GPT2, and ACLY genes in response to CSE, whilst down/over‐regulation of ACLY reduced GPT2 sensitivity to CSE. It evidences that those metabolism‐associated genes interact, although the exact mechanism remains unclear. One possibility is that altered target gene expression may lead to a compensative metabolic reprogramming and positive/negative signalling, since the conversation of mitochondrial glutamate and α‐KG can be affected by several enzymes, for example, GLUD1, glutamate dehydrogenase 1 (GDH1), GOT2, and GPT2.^45^ This study first evaluated CSE dose‐ and time‐dependent changes of those genes, roles of GPT2 in that gene expression, potential inter‐gene effects amongst, and influences in metabolomic and lipidomic profile changes. The interaction between GPT2 and ACLY genes was accompanied by altered levels of intracellular glutamic and citric acids. It implies that the maintenance of GPT2 activation may stabilise CSE‐induced hyper‐metabolism, which may be a new alternative to developing precision therapy for chronic diseases. It is also important to clarify whether intra‐mitochondrial or intracytoplasmic GPT2‐oriented metabolism triangles are more sensitive to CSE challenge and whether the cell metabolism‐specific compensatory responses and pathways exist to reshape cell metabolic pathways, balance intraorganellar communication, and promote organelle resilience, like cell lineage‐specific mitochondrial resilience.^46^

The importance of airway epithelial reprogramming was recognised as early changes in epithelial remodelling and COPD development, for example, smoking‐induced reprogramming of airway epithelial basal progenitor cells.^47^ Of many potential mechanisms by which the airway epithelial reprogramming was initiated, inflammatory factors (e.g., transforming growth factor) could induce the epithelial de‐differentiation through the epithelial‐to‐mesenchymal transition, responsible for the tissue remodelling, airflow obstruction, and micro‐airway fibrosis in COPD.^48^ Re‐patterning and reprogramming of cellular metabolism were suggested as the critical driver for airway remodelling in COPD, evidenced by the fact that alterations in glycolysis, glutamine, and fatty acid metabolism remodeled airway smooth muscle cell growth in COPD.^49^ In addition, metabolic reprogramming was also proposed as a driver for the pathogenesis of cigarette smoke‐induced inflammatory lung diseases and a therapeutic target pathway for airway information,^50^ although little has been known about airway epithelial metabolic reprogramming in lung diseases. Our results suggest a central and controlling role for GPT2 in smoking‐induced metabolic reprogramming, metabolism‐related transcriptional balance, and apoptosis in airway epithelial cells. This study initially presented the landscape of smoking‐induced airway epithelial lipidomic reprogramming characterised by alterations of LPCs, PCs, PEs, TAGs, and SMs in a dose‐dependent pattern. GPT2 is a key driver in the occurrence of CSE‐induced lipid metabolism. Our data evidence that GPT2 plays an important role in epithelial sensitivities and responses of lipidomic reprogramming and regulation of lipid‐associated transcriptional expressions. For example, CSE increased levels of PC (16:0, 18:0, 18:1, 18:2) and TAG (50, 52, 54, 57, 44−48) in the down‐regulation of GPT2, the contrary of that in the up‐regulation of GPT2. It seems that GPT2 is one of the important controls in the maintenance of epithelial sensitivity to external stimuli by regulating the balance of the metabolism triangle centre functions and preventing the occurrence of secondary metabolic reprogramming. Our data illustrate that the GPT2 down‐regulation per se alters the expression of many transcriptional factors, including metabolism‐, inflammation‐, and signal pathway‐associated genes, and improves CSE‐induced epithelial apoptosis. It indicates that other metabolisms and signal pathways changed by GPT2 deletion may also play necessary roles in the maintenance of the cell function and have the compensational effects.

Lipid reprogramming, including lipid dynamic changes, turnovers between organelles, and imbalance between storage and consumption, may control airway epithelial capacities of homeostatic maintenance in oxidative stress, inflammation, vitality, carcinogenesis, and remodelling.^51^, ^52^ GPT2 is one of the critical factors in regulating airway epithelial sensitivity and survival in response to smoking, through GPT2‐dominated reprogramming of lipid metabolisms. GPT2 regulates cell survival probably through its metabolites, since glutamate‐induced cell apoptosis at early stages and necrosis at later stages.^53^, ^54^, ^55^, ^56^ Furthermore, this study demonstrated that the reprogramming of lipid metabolism could be one of the important players in GPT2‐regulated cell survival since levels of CSE‐increased ACLY and TAG altered in epithelial cells when GPT2 was down‐regulated, whilst CSE‐increased PC was prevented when GPT2 was up‐regulated. We found that smoking‐altered lipids (e.g., PC and PE) were mainly involved in the glycerophospholipid metabolism pathway in airway epithelial cells. Levels of glycerophospholipid metabolites in COPD patients were negatively correlated with the generation of oxidative stress products and the severity of COPD.^57^ GPT2 regulates the epithelial cell sensitivity in response to external pathogens, since altered contents of LDs, the occurrence of epithelial apoptosis, and the reprogramming of lipid metabolites were highly dependent upon the up/down‐regulation of GPT2. As part of lipid reprogramming, the content and turnover of those lipid metabolites between intra‐ and extra‐epithelial microenvironments altered along with GPT2‐regulated cell sensitivity, although the exact mechanisms by which GPT2 regulates lipid switch and dynamics remain unclear. Of smoking‐induced lipidomic changes, PC and TAG levels were closely correlated with GPT2 expression, similar to the findings from COPD patients. For example, PC (34:3) and TAG (52:3) increased with the severity of oxidative stress and smoking status and were correlated with altered lung function and disease severity.^58^

GPT2 plays control roles in epithelial lipid reprogramming in response to smoking, probably through regulating the interactions amongst metabolism‐associated genes, refreshing mitochondrial function, and switching amongst PI3K isoform signals. Of smoking‐upregulated multiple genes, the regulation of GPT2 reduced epithelial sensitivity in four patterns, for example, both up‐ or down‐regulations had effects on GPT1, FASN, DGAT1, and CHIPT1, down‐regulation on GPT1 and ACLY, up‐regulation on ATF6 and PERK, or none on SELENOI. It indicates the complexity of molecular mechanisms by which GPT2 dominates the metabolic reprogramming as well as the difficulty to monitor the activities of metabolic proteins which are dependent upon both protein contents and activations. It is probably due to the less specificity, overlapping activities, and different subcellular locations of metabolic limiting enzymes. For example, about half of intracellular PC can be synthesised through CHPT1 in the Golgi apparatus and about the half or majority through choline/ethanolamine phosphotransferase 1 in the ER.^59^ We found that GPT2 up/down‐regulated epithelial cells had less sensitivity of CHPT1 expression to smoking, whilst CHPT1 up/down‐regulated cells had less responses of GPT2 to smoking, even though CHPT1 expression was higher in GPT2 up‐regulated cells. Different from CHPT1, DGAT1 up‐ or down‐regulation induced high or low sensitivities of ACLY and GPT2 expression to smoking, respectively. The esterification of DAG by DGAT1 controls TAG synthesis and turnover. It seems that DGAT1 has a clear regulatory effect on ACLY and GPT2. Smoking induced the interactions and expressions amongst metabolism‐associated genes as well as the corresponding metabolic reprogramming, probably through the activation of PI3K signal pathways. PIK3CA was found to regulate GPT2 expression through activation of 3–phosphoinositide‐dependent kinase and mitogen‐activated protein kinase, responsible for cell proliferation.^60^ We noticed that CSE increased the expression of PIK3CA, PIK3CB, PIK3CD, and PIK3R4 mRNA in a GPT2‐independent pattern, whilst ACLY, CHPT1, and DGAT1 contributed to the expression of PIK3CD which encodes the p110δ catalytic subunit of PI3K.^61^ This indicates that some of the metabolism‐associated genes have regulatory roles in PI3K‐dominated signal pathways. The GPT2 expression and levels of citric acids increased in epithelial cells by inhibiting PI3K subsets (α/β/δ, δ, α/δ, γ, or α/γ/δ/β), whilst reduced after smoking.

In conclusion, we measured circulating metabolomic and lipidomic profiles of patients with COPD and human airway epithelial metabolic and lipidomic profiles after smoking and defined the important roles of glutamate metabolisms. Of smoking‐upregulated mechanism genes, the down‐regulation of GPT2 and ACLY altered the epithelial sensitivity of metabolism gene expression and metabolites to smoking. The reprogramming of lipid metabolisms induced by smoking was refreshed by GPT2‐dominated regulation, especially PCs and TAGs, accompanied by altered transcriptomic expressions of lipid metabolism‐associated genes. GPT2 regulated cell sensitivities and survival to smoking through activating mitochondrial functions and stabilising lipid and energy homeostasis. The interactions between lipid metabolism genes, between metabolism genes and PI3K subset genes, and between metabolites and genes may contribute to the development of GPT2‐regulated lipid reprogramming during smoking. Thus, our data evidence important roles of GPT2 in airway epithelial lipid reprogramming after smoking and molecular mechanisms of GPT2‐dominated regulation, which can be an alternative to therapeutic strategies for chronic lung diseases.

## AUTHOR CONTRIBUTIONS

Furong Yan, Linlin Zhang and Lian Duan designed the study and patient specimen collection. Liyang Li, Xuanqi Liu and Yifei Liu completed the experimental process, data collection, literature search and generation of figures. Furong Yan and Xiangdong Wang wrote and edited the manuscript. Tiankui Qiao, Yiming Zeng, Hao Fang and Duojiao Wu provided the experimental technical and laboratory facilities support. All authors reviewed the manuscript. All authors read and approved the final manuscript.

## CONFLICT OF INTEREST STATEMENT

The authors declare no conflicts of interest.

## ETHICS APPROVAL

The study protocol was approved by Zhongshan Hospital's Ethical Evaluation Committee approved the present study [B2019‐197(2)]. Written informed consents were obtained from all participants upon enrollment. Throughout the study, all patient samples were de‐identified.

## Supporting information

Supporting Information

Supporting Information

Supporting Information

Supporting Information

Supporting Information

Supporting Information

Supporting Information

## Data Availability

The datasets used and/or analysed during the current study are available from the corresponding author on reasonable request.
