# Supplementary figures and images for: Roles of glutamic pyruvate transaminase 2 in reprogramming of airway epithelial lipidomic and metabolomic profiles after smoking
Source: Clin Transl Med. 2024 May 5;14(5):e1679. doi: 10.1002/ctm2.1679 (PMC11070440; doi:10.1002/ctm2.1679)

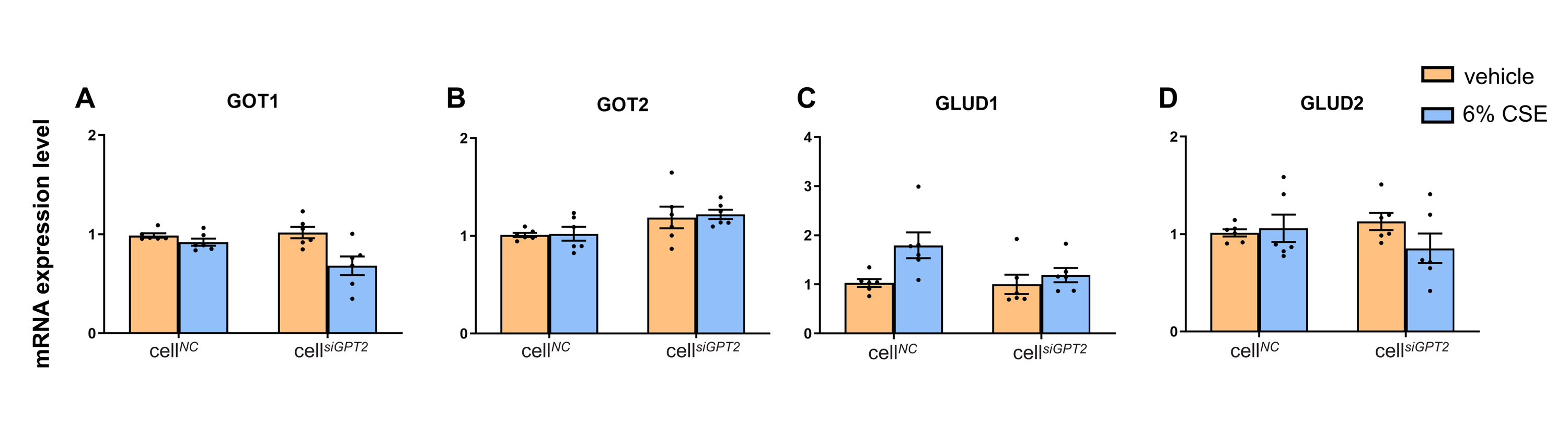

Supplement: Supplementary file 1 — Supporting Information [file CTM2-14-e1679-s007.jpg]

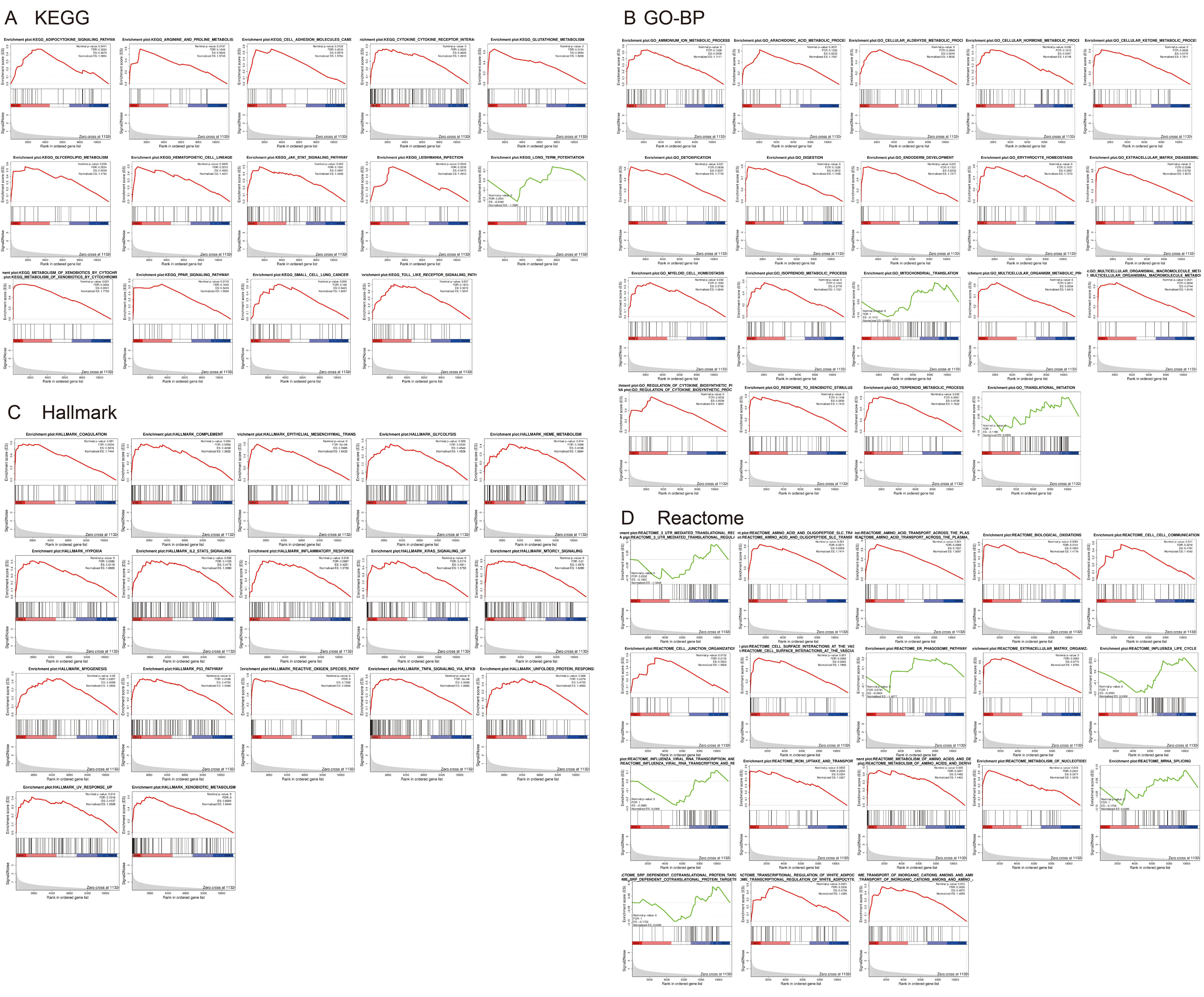

Supplement: Supplementary file 2 — Supporting Information [file CTM2-14-e1679-s006.jpg]

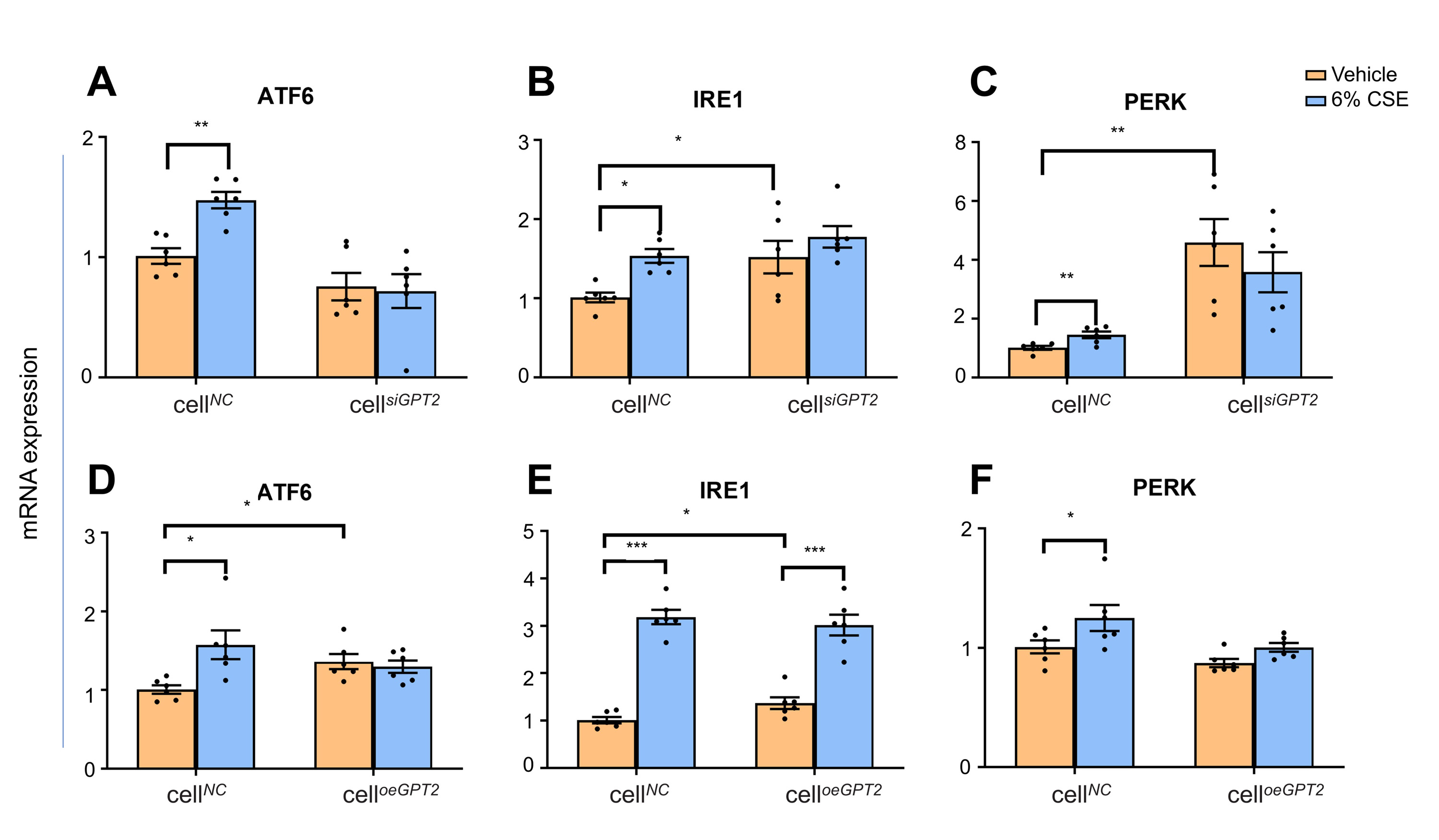

Supplement: Supplementary file 3 — Supporting Information [file CTM2-14-e1679-s005.jpg]

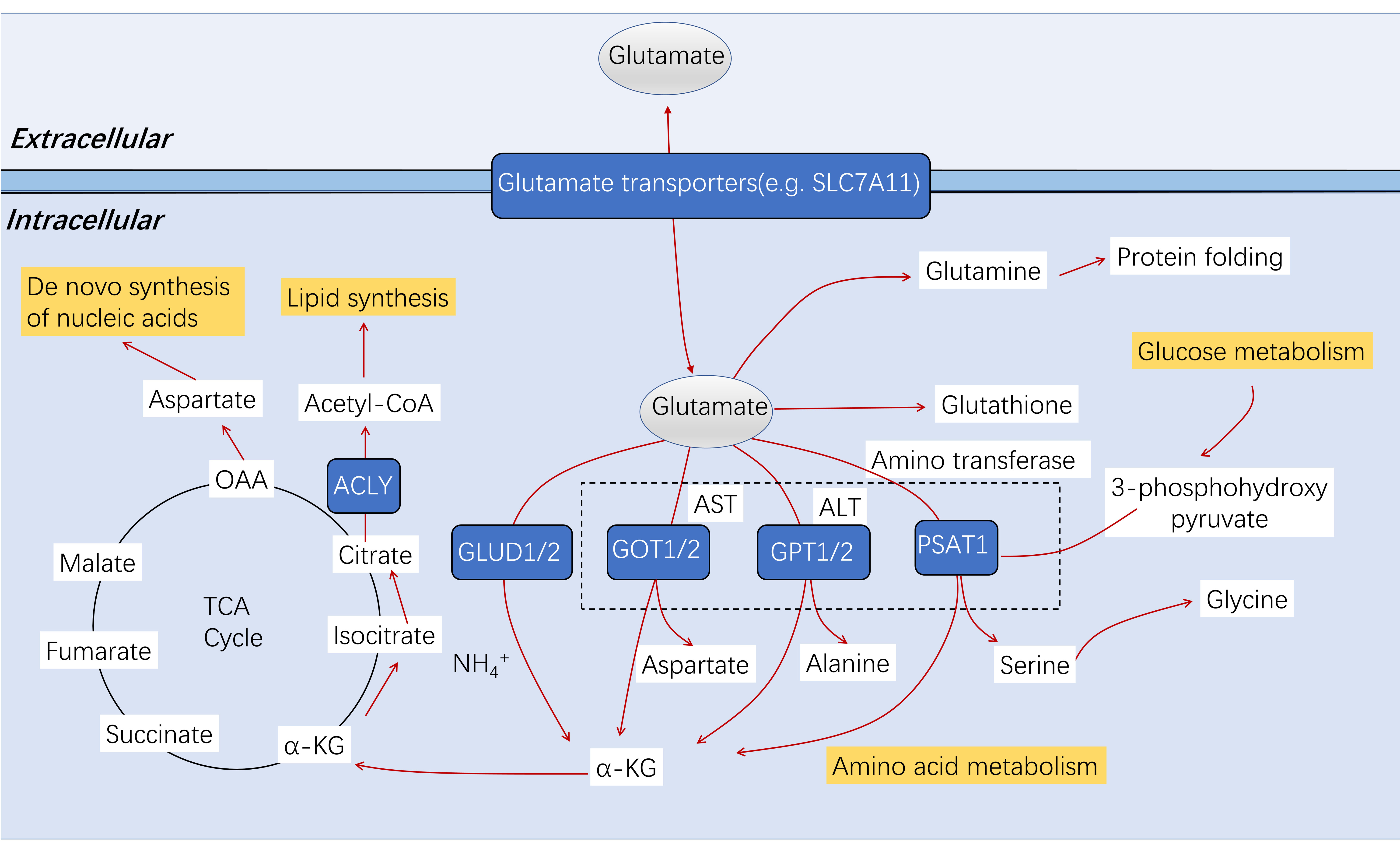

Supplement: Supplementary file 4 — Supporting Information [file CTM2-14-e1679-s004.jpg]
