# Supplementary material for: Roles of glutamic pyruvate transaminase 2 in reprogramming of airway epithelial lipidomic and metabolomic profiles after smoking
Source: Clin Transl Med. 2024 May 5;14(5):e1679. doi: 10.1002/ctm2.1679 (PMC11070440; doi:10.1002/ctm2.1679)
Supplement: Supplementary file 5 — Supporting Information [file CTM2-14-e1679-s001.docx]

|  | Healthy control (N=12) | COPD (N=12) ^*^ |
| --- | --- | --- |
| Gender (Male, N) | 9 | 12 |
| Age (Years) | 38.00±11.60 | 68.58±7.08 |
| BMI | 23.79±3.84 | 24.32±5.61 |

**Table S1.** Demographic data of the clinical participants.

^*^These patients were diagnosed as COPD according to Global Initiative for Chronic Obstructive Lung Disease (GOLD).

**Table S2.** Primers for RT-qPCR.

|  | Forward | Reverse |
| --- | --- | --- |
| **human** |  |  |
| *SLC7A11* | CGGTGGTGTGTTTGCTGTCTCC | CAGAGTGATGACGAAGCCAATCCC |
| *GLUD1* | TGATAATGCCTCGTCAGTGTTCTTGG | CGCTCACCTGTGCTCATGTCTG |
| *GLUD2* | AATCCAACGCACCCAGAGTCAAAG | GAACAGACAGGAGCAAGTGGTAGTTAG |
| *GOT1* | ATGCTCCTGAGTTCTCCATTGTTGTC | CCAAGTAATCCGCACGATCTTCTCC |
| *GOT2* | GGAAACCACACACCCATCTTCAGG | GGCATCCTTATCACCATCACCACTG |
| *GPT1* | GTGTCATCAACCCTGGCAACCC | AGGTGGAGTGGAAGGAGGCAAG |
| *GPT2* | CCTCCTTCCACTCCACCTCCAAG | GACTCCTTCTCTCGGCTGAATTGC |
| *PSAT1* | GGAAGGTGTGCTGACTATGTGGTG | GGCTTGGACAGGAAGTTTGAGGAC |
| *ACLY* | GCGAGCAGCAGACCTATGACTATG | TCTTCCCGACTTCTCCCATCACC |
| *FASN* | GCCUGAAGAAGUCCUUCUATT | UAGAAGGACUUCUUCAGGCTT |
| *DGAT1* | ACTCCGAGTCTGTCACCTACTTCTG | GGCTATTGGCTGTCCGATGATGAG |
| *CHPT1* | ATTGCCGCTCGCTTAGGAACTTATC | TCCATTCTTGCCAACACCACCATG |
| *SELENOI* | TTCTGGCTTTCTGCTGGTCGTATTC | GTGCTTGTGACCTGGTGCTGAG |
| *ATF6* | GCGGAGCCACTGAAGGAAGATAAG | TGTTTGAGTCTTGGGTGCTGCTG |
| *PERK* | ATGGATGATGTGGTCAAGGTTGGAG | TGTCTGGCATAAGCTGGCATTGG |
| *IRE1* | GCAAGAGTATGTGGAGCAGAAGGAC | CTGTGAACGATGTTGAGGGAGTGG |
| *PIK3CA* | CGGTGACTGTGTGGGACTTATTGAG | TGTAGTGTGTGGCTGTTGAACTGC |
| *PIK3CB* | GAGATTGCAAGCAGTGATAGTG | TAATTTTGGCAGTGATTGTGGG |
| *PIK3CD* | GACCCAGAAGTGAACGACTTTC | CCTCAAACTTAACGTTGACCAG |
| *PIK3CG* | CACCCAAAAGCATATCCTAAGC | GTAATGCAGAACATCATCGTCC |
| *PIK3R1* | GCTGCTATGCCTGCTCTGTAGTG | CGCCTCTGCTGTGCATATACTGG |
| *PIK3R2* | CAGGAACACTTGGAAGAGCAGGAG | GTGGCGGTAGTGATTGATGAGGTC |
| *PIK3R3* | TCCGAGATCAACACCTTGTATGGC | ACCACAGAGCAAGCATAGCATCC |
| *PIK3R4* | CCGAATTGGAGGACGAGTCAAGAC | TCACAGTGGCATAGGCAAGAACAG |
| **mouse** |  |  |
| *Acly* | TGTGATGAGCGAGGGCAGGAG | CTGGCAGGAATACTTGGGCAACC |
| *Gpt2* | AGGTGAAGGCGGTGGAGTACG | AATGTTGGCTCGGATTACCTCAGTG |

**Table S3.** Differential metabolites between control and COPD groups.

| COPD group vs Control group | | |
| --- | --- | --- |
| Metabolites name | Fold change | *P*-value |
| 2-amino-2-methyl-1,3-propanediol | 7.186299 | 4.31E-13 |
| N-acetyl-5-hydroxytryptamine | 2.749925 | 2.41E-08 |
| stearic acid | 2.328695 | 6.17E-09 |
| urea | 2.247535 | 6.72E-05 |
| palmitic acid | 2.174988 | 6.91E-07 |
| trans-13-octadecenoic acid | 2.156232 | 5.34E-07 |
| elaidic acid | 2.153155 | 5.11E-07 |
| N, N-dimethylglycine | 2.104298 | 6.03E-07 |
| D-mannose | 2.045472 | 2.06E-06 |
| cholesterol | 2.044336 | 0.000111 |
| glyoxylic acid | 1.841929 | 8.8E-06 |
| thymol | 1.74727 | 8.49E-06 |
| linoleic acid | 1.73288 | 0.007903 |
| oxalic acid | 1.584005 | 0.039948 |
| palatinitol | 1.287377 | 0.018366 |
| tyrosine | 0.518657 | 0.000158 |
| 1,5-anhydro-D-sorbitol | 0.491458 | 0.002968 |
| L-glutamine | 0.456044 | 0.004488 |
| L-threonine | 0.453401 | 1.2E-05 |
| allo-inositol | 0.445356 | 5.07E-05 |
| L-lysine | 0.436819 | 9.86E-05 |
| L-allothreonine | 0.431707 | 0.000375 |
| glycerol | 0.412444 | 1.62E-06 |
| L-glutamic acid | 0.392445 | 7.01E-07 |
| L-norleucine | 0.378159 | 4.53E-08 |
| L- (+) lactic acid | 0.372038 | 8.46E-05 |
| DL-isoleucine | 0.371403 | 0.000788 |
| N-acetyl-ornithine | 0.356129 | 0.008332 |
| glycine | 0.35123 | 6.12E-06 |
| L-ornithine | 0.340308 | 0.000249 |
| citric acid | 0.329825 | 1.16E-07 |
| L-valine | 0.287571 | 8.74E-07 |
| L-proline | 0.280702 | 3.51E-05 |
| L- sorbose | 0.260017 | 0.028018 |
| iminodiacetic acid | 0.246459 | 9.24E-05 |
| L-alanine | 0.200214 | 7.66E-06 |
| 5-aminovaleric acid | 0.190979 | 0.021193 |
| L-serine | 0.177891 | 3.93E-05 |
| glycolic acid | 0.149838 | 7.43E-07 |
| phenylacetaldehyde | 0.114188 | 0.022537 |
| arabitol | 0.083814 | 4.76E-18 |
| D-allose | 0.057344 | 1.34E-07 |
| psicose | 0.027045 | 0.000409 |
| D-lyxose | 0.026769 | 0.000137 |
| D-mannitol | 0.012919 | 5.99E-06 |
| pyruvic acid | 0.011792 | 1.12E-08 |
| eicosapentaenoic acid | 0.006321 | 6.3E-11 |

**Table S4.** The top 30 up-regulated and top 30 down-regulated lipids between control and COPD groups.

| COPD group vs Control group | | |
| --- | --- | --- |
| Lipids name | Fold change | *P*-value |
| TAG46:2-FA14:0 | 16.73101 | 0.015791 |
| TAG47:2-FA14:0 | 12.27504 | 0.012913 |
| PE (O-16:0/16:1) | 11.9638 | 0.004235 |
| TAG48:1-FA18:0 | 9.407179 | 0.003317 |
| TAG48:5-FA18:2 | 8.145628 | 0.001814 |
| TAG46:3-FA16:0 | 7.667316 | 0.004324 |
| TAG44:2-FA18:2 | 7.597009 | 0.023269 |
| TAG46:3-FA18:1 | 7.202334 | 0.02049 |
| PC (16:0/20:4) | 7.15672 | 2.73E-06 |
| TAG46:2-FA16:1 | 6.120283 | 0.027898 |
| TAG46:2-FA16:0 | 5.969792 | 0.007556 |
| TAG46:4-FA18:2 | 5.796092 | 0.045465 |
| TAG48:4-FA16:0 | 5.768138 | 0.003851 |
| TAG46:2-FA18:2 | 5.638377 | 0.006276 |
| TAG44:1-FA16:0 | 5.521481 | 0.036822 |
| TAG44:2-FA18:1 | 5.291897 | 0.015863 |
| TAG48:4-FA18:2 | 5.268501 | 0.007583 |
| PC (16:1/18:1) | 4.778919 | 7.99E-06 |
| TAG47:1-FA14:0 | 4.512975 | 0.025342 |
| TAG48:3-FA18:1 | 4.061629 | 0.004007 |
| TAG46:1-FA14:0 | 3.932069 | 0.037627 |
| PC (18:0/16:1) | 3.849624 | 0.00755 |
| TAG46:1-FA16:0 | 3.721758 | 0.031822 |
| TAG48:3-FA14:0 | 3.613057 | 0.034817 |
| TAG46:1-FA18:1 | 3.586067 | 0.031611 |
| TAG48:4-FA16:1 | 3.385573 | 0.044356 |
| TAG44:1-FA14:0 | 3.316955 | 0.046614 |
| PE (16:0/16:1) | 3.259474 | 0.015601 |
| TAG48:2-FA18:1 | 3.092313 | 0.015158 |
| TAG46:2-FA18:1 | 3.012513 | 0.028032 |
| TAG56:7-FA18:3 | 0.041882 | 0.001451 |
| TAG54:8-FA20:5 | 0.041403 | 0.029227 |
| TAG52:7-FA22:6 | 0.039294 | 0.013547 |
| TAG54:6-FA20:5 | 0.039196 | 0.023402 |
| TAG52:5-FA20:5 | 0.038263 | 0.015441 |
| TAG54:5-FA20:5 | 0.037987 | 0.019077 |
| TAG56:7-FA22:6 | 0.037437 | 0.045295 |
| TAG52:4-FA18:3 | 0.037392 | 0.001134 |
| TAG52:6-FA22:6 | 0.036848 | 0.011968 |
| TAG52:3-FA18:3 | 0.03677 | 0.000883 |
| TAG56:8-FA18:3 | 0.036602 | 0.008505 |
| TAG54:5-FA22:5 | 0.035388 | 0.003234 |
| TAG56:6-FA22:6 | 0.034885 | 0.032959 |
| TAG54:7-FA22:5 | 0.034822 | 0.004054 |
| TAG56:9-FA18:3 | 0.034784 | 0.000537 |
| TAG54:6-FA22:5 | 0.033288 | 0.001854 |
| TAG54:8-FA22:6 | 0.032806 | 0.013611 |
| TAG52:5-FA18:3 | 0.031275 | 0.001356 |
| TAG52:6-FA18:3 | 0.030574 | 0.007457 |
| TAG54:4-FA18:3 | 0.028996 | 0.001774 |
| TAG54:7-FA22:6 | 0.028301 | 0.011412 |
| TAG54:5-FA18:3 | 0.025636 | 0.001386 |
| DAG (16:0/18:2) | 0.023159 | 0.000181 |
| TAG54:3-FA18:3 | 0.023018 | 0.00274 |
| TAG54:6-FA18:3 | 0.020969 | 0.004864 |
| TAG54:7-FA18:3 | 0.018578 | 0.004694 |
| TAG54:8-FA18:3 | 0.018505 | 0.005872 |
| TAG54:6-FA22:6 | 0.017705 | 0.023753 |
| DAG (18:1/18:2) | 0.015435 | 0.000465 |
| TAG54:5-FA18:2 | 0.004489 | 9.36E-06 |
