# Supplementary material for: Roles of glutamic pyruvate transaminase 2 in reprogramming of airway epithelial lipidomic and metabolomic profiles after smoking
Source: Clin Transl Med. 2024 May 5;14(5):e1679. doi: 10.1002/ctm2.1679 (PMC11070440; doi:10.1002/ctm2.1679)
Supplement: Supplementary file 6 — Supporting Information [file CTM2-14-e1679-s003.docx]

**Table S5.** Differences of intracellular metabolites in 16-HBE cells stimulated with different concentrations of CSE for 12 hours.

| 3% CSE vs vehicle | | 6% CSE vs vehicle | | 10% CSE vs vehicle | |
| --- | --- | --- | --- | --- | --- |
| Metabolites | FC | Metabolites | FC | Metabolites | FC |
| isopropyl beta-D-1-thiogalactopyranoside | 9.707527 | isopropyl beta-D-1-thiogalactopyranoside | 14.09223 | isopropyl beta-D-1-thiogalactopyranoside | 10.78147 |
| galactinol | 7.894041 | adenine | 13.74291 | galactinol | 9.524381 |
| methyl-beta-D-galactopyranoside | 5.604089 | galactinol | 11.21184 | methyl-beta-D-galactopyranoside | 6.835931 |
| L- sorbose | 3.838233 | inosine | 9.132946 | adenine | 6.383202 |
| arachidic acid | 2.721164 | L- sorbose | 8.781199 | L- sorbose | 3.246857 |
| citric acid | 2.366865 | methyl-beta-D-galactopyranoside | 8.708808 | xanthine | 1.935283 |
| adenine | 2.143082 | glycolic acid | 4.60968 | citramalic acid | 1.790369 |
| N-acetyl-D-glucosamine | 1.834258 | 1 monostearin | 4.413513 | cysteinylglycine | 1.717421 |
| inosine | 1.804798 | pipecolic acid | 3.092287 | L-cysteine | 1.658084 |
| cysteinylglycine | 1.702806 | urea | 2.489024 | D-malic acid | 1.657783 |
| glycerol 1-phosphate | 1.670327 | L-norleucine | 2.477067 | citric acid | 1.633943 |
| L-cysteine | 1.611044 | D-malic acid | 2.418768 | pipecolic acid | 1.519281 |
| D-glucose-6-phosphate | 1.544332 | succinic acid | 2.161861 | putrescine | 1.449234 |
| pyruvic acid | 1.534585 | 2-hydroxypyridine | 2.132599 | L-glutamic acid | 1.416573 |
| 1 monostearin | 1.534162 | citramalic acid | 2.096042 | 2,3-butanediol | 1.309534 |
| L-glutamic acid | 1.495592 | putrescine | 2.073709 | fumaric acid | 1.250824 |
| spermidine | 1.455022 | L-threonine | 2.070939 | trans-4-hydroxy-L-proline | 1.213093 |
| phosphoric acid | 1.433085 | 2,3-butanediol | 2.060822 | hypoxanthine | 1.145582 |
| L-lysine | 1.433038 | 5-aminovaleric acid | 1.918641 | allo-inositol | 1.1205 |
| citrulline | 1.348786 | L-serine | 1.776762 | phosphoric acid | 1.098208 |
| 5-aminovaleric acid | 1.321032 | pyruvic acid | 1.659978 | ethanolamine | 1.093235 |
| L-mimosine | 1.320287 | ethanolamine | 1.659244 | L-tryptophan | 1.07885 |
| L-valine | 1.317839 | oxalic acid | 1.612245 | tyrosine | 1.074037 |
| citramalic acid | 1.315679 | phosphoric acid | 1.550508 | urea | 0.903322 |
| putrescine | 1.315092 | benzoic acid | 1.538367 | pyruvic acid | 0.833068 |
| pipecolic acid | 1.303797 | spermidine | 1.513139 | arachidic acid | 0.800869 |
| tyrosine | 1.286324 | L-methionine | 1.511281 | N-acetyl-L-aspartic acid | 0.799363 |
| 6-phosphogluconic acid | 1.285753 | L-valine | 1.507269 | aspartic acid | 0.79442 |
| 2,3-butanediol | 1.26629 | 3-hydroxypyridine | 1.499028 | 1 monostearin | 0.787542 |
| L-tryptophan | 1.254508 | trans-4-hydroxy-L-proline | 1.498458 | benzoic acid | 0.782018 |
| uracil | 1.21093 | L-glutamic acid | 1.496759 | spermidine | 0.777576 |
| L-ornithine | 1.207703 | arachidic acid | 1.489634 | oxalic acid | 0.742289 |
| hypoxanthine | 1.201202 | 1,3-propanediol | 1.486726 | 2-amino-2-methyl-1,3-propanediol | 0.740889 |
| L-methionine | 1.199326 | L-lysine | 1.439273 | glyceric acid | 0.724647 |
| ethanolamine | 1.187105 | glycine | 1.428299 | 5-aminovaleric acid | 0.686407 |
| L-asparagine | 1.178027 | N, N-dimethylglycine | 1.417212 | uric acid | 0.674773 |
| glycine | 1.156235 | 2-amino-2-methyl-1,3-propanediol | 1.408011 | 3-phosphoglyceric acid | 0.65411 |
| D-malic acid | 1.106535 | L-alanine | 1.40554 | 2-hydroxypyridine | 0.649279 |
| cholesterol | 1.100564 | L-ornithine | 1.383681 | inosine | 0.637381 |
| acetohydroxamic acid | 1.079445 | L-proline | 1.380228 | O-phosphocolamine | 0.637143 |
| 1,3-propanediol | 1.066269 | tyrosine | 1.35822 | palmitoleic acid | 0.633939 |
| palmitoleic acid | 0.879779 | citrulline | 1.321306 | 6-phosphogluconic acid | 0.61942 |
| 3-phosphoglyceric acid | 0.825281 | acetohydroxamic acid | 1.316442 | 3-hydroxypyridine | 0.591174 |
| xanthine | 0.787876 | ribose | 1.295855 | acetohydroxamic acid | 0.576415 |
| allo-inositol | 0.776497 | aspartic acid | 1.171217 | myristic acid | 0.559645 |
| phosphoenolpyruvic acid | 0.728951 | hypoxanthine | 0.924375 | phosphoenolpyruvic acid | 0.559302 |
| myristic acid | 0.676999 | glyceric acid | 0.867752 |  |  |
| uric acid | 0.434536 | methyl linolenate | 0.730663 |  |  |
|  |  | myristic acid | 0.687008 |  |  |
|  |  | 6-phosphogluconic acid | 0.679924 |  |  |
|  |  | xanthine | 0.650648 |  |  |
|  |  | D-glucose-6-phosphate | 0.622108 |  |  |
|  |  | O-phosphocolamine | 0.621824 |  |  |
|  |  | palmitoleic acid | 0.619076 |  |  |
|  |  | 3-phosphoglyceric acid | 0.566356 |  |  |
|  |  | N-acetyl-L-aspartic acid | 0.524194 |  |  |
|  |  | L-asparagine | 0.50787 |  |  |
|  |  | L-mimosine | 0.478831 |  |  |
|  |  | uric acid | 0.450241 |  |  |
|  |  | L-cysteine | 0.286503 |  |  |
|  |  | cysteinylglycine | 0.105246 |  |  |
|  |  | phosphoenolpyruvic acid | 0.098491 |  |  |

**Table S6.** Differences of intracellular metabolites in 16-HBE cells stimulated with different concentrations of CSE for 24 hours.

| 3% CSE vs vehicle | | 6% CSE vs vehicle | | 10% CSE vs vehicle | |
| --- | --- | --- | --- | --- | --- |
| Metabolites | FC | Metabolites | FC | Metabolites | FC |
| cysteinylglycine | 2.927401 | citric acid | 3.977511 | adenine | 2.759906 |
| arachidic acid | 2.63089 | cysteinylglycine | 2.772552 | D-ribose-5-phosphate | 2.206638 |
| pyruvic acid | 1.43885 | succinic acid | 1.822342 | arachidic acid | 2.033001 |
| 2,3-butanediol | 1.310722 | N-acetyl-D-glucosamine | 1.819508 | pipecolic acid | 1.979403 |
| benzoic acid | 1.295421 | inosine | 1.80904 | citric acid | 1.760986 |
| 2-hydroxypyridine | 1.224176 | 6-phosphogluconic acid | 1.47356 | 2,3-butanediol | 1.724296 |
| spermidine | 1.213659 | 2,3-butanediol | 1.401395 | xanthine | 1.693559 |
| N-acetyl-L-aspartic acid | 1.087684 | pipecolic acid | 1.36728 | inosine | 1.456558 |
| N, N-dimethylglycine | 1.075076 | citramalic acid | 1.364434 | citramalic acid | 1.450139 |
| L-glutamic acid | 1.073077 | arachidic acid | 1.323607 | methyl-beta-D-galactopyranoside | 1.328338 |
| ethanolamine | 1.053438 | L-glutamic acid | 1.199114 | benzoic acid | 1.304358 |
| uracil | 1.052584 | fumaric acid | 1.19012 | spermidine | 1.271998 |
| 2-amino-2-methyl-1,3-propanediol | 0.971814 | L-cysteine | 1.166063 | galactinol | 1.269464 |
| 1,3-propanediol | 0.966979 | N, N-dimethylglycine | 1.121577 | oxalic acid | 1.22965 |
| cholesterol | 0.916269 | ethanolamine | 1.02778 | glycerol 1-phosphate | 1.214479 |
| acetohydroxamic acid | 0.915769 | L-lysine | 0.956081 | phosphoric acid | 1.136713 |
| 3-phosphoglyceric acid | 0.887483 | tyrosine | 0.931788 | L-glutamic acid | 1.086298 |
| phosphoenolpyruvic acid | 0.86044 | 2-hydroxypyridine | 0.904454 | putrescine | 0.940556 |
| palmitoleic acid | 0.858706 | acetohydroxamic acid | 0.892343 | acetohydroxamic acid | 0.914253 |
| D-malic acid | 0.858099 | phosphoric acid | 0.887895 | fumaric acid | 0.87661 |
| L-tryptophan | 0.83263 | 2-amino-2-methyl-1,3-propanediol | 0.880156 | 1,3-propanediol | 0.870389 |
| glyceric acid | 0.785173 | 1,3-propanediol | 0.877987 | N, N-dimethylglycine | 0.863069 |
| N-ethylglycine | 0.781793 | 3-hydroxypyridine | 0.877399 | D-malic acid | 0.847681 |
| glycine | 0.767762 | myristic acid | 0.870378 | 2-amino-2-methyl-1,3-propanediol | 0.826331 |
| L-cysteine | 0.76289 | N-acetyl-L-aspartic acid | 0.866081 | tyrosine | 0.81954 |
| citrulline | 0.760452 | glycine | 0.862108 | 6-phosphogluconic acid | 0.812237 |
| ribose | 0.747675 | glycerol 1-phosphate | 0.850315 | L-tryptophan | 0.768579 |
| L-alanine | 0.744064 | cholesterol | 0.810335 | L-lysine | 0.767439 |
| L-valine | 0.742752 | D-glucose-6-phosphate | 0.808179 | 3-phosphoglyceric acid | 0.761065 |
| tyrosine | 0.738392 | L-valine | 0.806045 | L-mimosine | 0.758424 |
| 5-aminovaleric acid | 0.711328 | urea | 0.746511 | citrulline | 0.743678 |
| trans-4-hydroxy-L-proline | 0.705466 | L-ornithine | 0.745787 | cholesterol | 0.727335 |
| L-lysine | 0.690038 | putrescine | 0.683674 | hypoxanthine | 0.724849 |
| adenine | 0.688601 | O-phosphocolamine | 0.67963 | N-ethylglycine | 0.723229 |
| L-methionine | 0.66612 | trans-4-hydroxy-L-proline | 0.647141 | uracil | 0.703861 |
| putrescine | 0.655733 | glyceric acid | 0.63108 | L-alanine | 0.680113 |
| L-ornithine | 0.650927 | 3-phosphoglyceric acid | 0.604884 | L-methionine | 0.669408 |
| citric acid | 0.645914 | spermidine | 0.580205 | glycine | 0.648118 |
| 6-phosphogluconic acid | 0.620272 | palmitoleic acid | 0.569894 | L-ornithine | 0.647983 |
| aspartic acid | 0.585679 | phosphoenolpyruvic acid | 0.550103 | L-valine | 0.634217 |
| allo-inositol | 0.558895 | allo-inositol | 0.50853 | L-asparagine | 0.5677 |
| isopropyl beta-D-1-thiogalactopyranoside | 0.459372 | aspartic acid | 0.438501 | N-acetyl-L-aspartic acid | 0.548545 |
| methyl-beta-D-galactopyranoside | 0.439343 | methyl-beta-D-galactopyranoside | 0.424134 | L-serine | 0.540536 |
| L- sorbose | 0.425554 | galactinol | 0.407209 | trans-4-hydroxy-L-proline | 0.514387 |
| galactinol | 0.413473 | uric acid | 0.40281 | allo-inositol | 0.511303 |
| D-glucose-6-phosphate | 0.313249 | isopropyl beta-D-1-thiogalactopyranoside | 0.398192 | phosphoenolpyruvic acid | 0.482575 |
|  |  | L- sorbose | 0.37822 | aspartic acid | 0.476993 |
|  |  | adenine | 0.169263 | L-proline | 0.460082 |
|  |  |  |  | glyceric acid | 0.435908 |
|  |  |  |  | palmitoleic acid | 0.407618 |
|  |  |  |  | uric acid | 0.342385 |
|  |  |  |  | L-threonine | 0.325779 |

**Table S7.** Differences of intracellular metabolites in 16-HBE cells stimulated with different concentrations of CSE for 48 hours.

| 3% CSE vs vehicle | | 6% CSE vs vehicle | | 10% CSE vs vehicle | |
| --- | --- | --- | --- | --- | --- |
| Metabolites | FC | Metabolites | FC | Metabolites | FC |
| 5-aminovaleric acid | 6.172094 | arachidic acid | 2.347452 | adenine | 19.0609 |
| pipecolic acid | 3.676686 | xanthine | 2.248306 | N-acetyl-D-mannosamine | 9.289361 |
| arachidic acid | 2.055625 | methyl linolenate | 1.811559 | xanthine | 7.842587 |
| Phenylalanine | 2.002157 | D-ribose-5-phosphate | 1.588512 | pyruvic acid | 3.545496 |
| N-acetyl-D-mannosamine | 1.359447 | spermidine | 1.46472 | methyl-beta-D-galactopyranoside | 3.006047 |
| N-acetyl-L-aspartic acid | 1.351978 | 1 monostearin | 1.418395 | galactinol | 2.833938 |
| 1 monostearin | 1.325068 | ethanolamine | 1.40228 | isopropyl beta-D-1-thiogalactopyranoside | 2.788021 |
| 2,3-butanediol | 0.965987 | isopropyl beta-D-1-thiogalactopyranoside | 1.342448 | glycolic acid | 2.678949 |
| 1,3-propanediol | 0.922981 | pipecolic acid | 1.265055 | D-ribose-5-phosphate | 2.575957 |
| galactinol | 0.9147 | O-phosphocolamine | 1.242357 | spermidine | 1.729365 |
| ribose | 0.901151 | D-malic acid | 1.23154 | pipecolic acid | 1.714008 |
| acetohydroxamic acid | 0.875732 | galactinol | 1.192169 | benzoic acid | 1.362229 |
| cholesterol | 0.872674 | methyl-beta-D-galactopyranoside | 1.180588 | 3-hydroxypyridine | 1.24484 |
| 3-phosphoglyceric acid | 0.844837 | L-glutamic acid | 1.121591 | aspartic acid | 1.240673 |
| 2-amino-2-methyl-1,3-propanediol | 0.840417 | uracil | 1.114885 | phosphoric acid | 1.202333 |
| N, N-dimethylglycine | 0.824215 | phosphoenolpyruvic acid | 1.09249 | 2-hydroxypyridine | 1.099798 |
| inosine | 0.818208 | phosphoric acid | 1.077542 | cholesterol | 0.950825 |
| ethanolamine | 0.803507 | 2,3-butanediol | 1.060049 | acetohydroxamic acid | 0.923858 |
| 6-phosphogluconic acid | 0.802927 | 1,3-propanediol | 0.91326 | 1,3-propanediol | 0.918311 |
| uracil | 0.798164 | cholesterol | 0.911069 | 2-amino-2-methyl-1,3-propanediol | 0.911812 |
| glyceric acid | 0.791485 | acetohydroxamic acid | 0.893084 | N, N-dimethylglycine | 0.787528 |
| citric acid | 0.773259 | 3-hydroxypyridine | 0.87594 | DL-isoleucine | 0.76895 |
| allo-inositol | 0.771075 | L-serine | 0.87295 | allo-inositol | 0.766998 |
| O-phosphocolamine | 0.770643 | tyrosine | 0.866798 | inosine | 0.724608 |
| L-valine | 0.758312 | DL-isoleucine | 0.864798 | 2,3-butanediol | 0.647382 |
| glycine | 0.752735 | 2-amino-2-methyl-1,3-propanediol | 0.8625 | citramalic acid | 0.603365 |
| L-proline | 0.752279 | L-leucine | 0.8497 | succinic acid | 0.596324 |
| L-tryptophan | 0.668632 | N, N-dimethylglycine | 0.848635 | trans-4-hydroxy-L-proline | 0.586478 |
| palmitoleic acid | 0.666927 | L-valine | 0.845167 | N-ethylglycine | 0.580748 |
| xanthine | 0.664123 | citric acid | 0.843493 | L-leucine | 0.44573 |
| tyrosine | 0.654802 | glycine | 0.839099 | Phenylalanine | 0.4177 |
| D-glucose-6-phosphate | 0.636 | citrulline | 0.837995 | uracil | 0.352501 |
| L-alanine | 0.633126 | citramalic acid | 0.80919 | tyrosine | 0.297556 |
| putrescine | 0.61427 | L-methionine | 0.804648 | 3-phosphoglyceric acid | 0.28677 |
| L-mimosine | 0.608373 | myristic acid | 0.795166 | citric acid | 0.262182 |
| spermidine | 0.605054 | putrescine | 0.789059 | urea | 0.260285 |
| oxalic acid | 0.592823 | L-proline | 0.784079 | L-valine | 0.243921 |
| glycerol 1-phosphate | 0.591914 | L-alanine | 0.777154 | L-tryptophan | 0.211832 |
| adenine | 0.511438 | trans-4-hydroxy-L-proline | 0.753845 | ethanolamine | 0.209073 |
| aspartic acid | 0.489181 | L-lysine | 0.746912 | L-proline | 0.206318 |
| L-methionine | 0.481449 | inosine | 0.692486 | palmitoleic acid | 0.199981 |
| L-threonine | 0.459644 | L-ornithine | 0.677305 | L-mimosine | 0.198191 |
| L-lysine | 0.422913 | L-cysteine | 0.671426 | L-norleucine | 0.188801 |
| trans-4-hydroxy-L-proline | 0.298879 | aspartic acid | 0.669877 | myristic acid | 0.184751 |
| citrulline | 0.232907 | allo-inositol | 0.570793 | glyceric acid | 0.182498 |
| L-cysteine | 0.156281 | uric acid | 0.494713 | L-lysine | 0.173694 |
| L-asparagine | 0.116262 | adenine | 0.46776 | glycine | 0.173295 |
| L-ornithine | 0.107878 | 6-phosphogluconic acid | 0.415789 | citrulline | 0.163699 |
|  |  | palmitoleic acid | 0.384404 | L-serine | 0.156302 |
|  |  | glycolic acid | 0.373269 | L-threonine | 0.154532 |
|  |  |  |  | uric acid | 0.148209 |
|  |  |  |  | L-asparagine | 0.141781 |
|  |  |  |  | 6-phosphogluconic acid | 0.140884 |
|  |  |  |  | N-acetyl-L-aspartic acid | 0.138927 |
|  |  |  |  | L-methionine | 0.138514 |
|  |  |  |  | D-malic acid | 0.138297 |
|  |  |  |  | O-phosphocolamine | 0.126339 |
|  |  |  |  | methyl linolenate | 0.124739 |
|  |  |  |  | 5-aminovaleric acid | 0.102983 |
|  |  |  |  | glycerol 1-phosphate | 0.102638 |
|  |  |  |  | L-alanine | 0.078074 |
|  |  |  |  | hypoxanthine | 0.060405 |
|  |  |  |  | L-cysteine | 0.046627 |
|  |  |  |  | fumaric acid | 0.027285 |
|  |  |  |  | phosphoenolpyruvic acid | 0.013349 |

**Table S8.** Differences of extracellular metabolites in 16-HBE cells stimulated with different concentrations of CSE for 12 hours.

| 3% CSE vs vehicle | | 6% CSE vs vehicle | | 10% CSE vs vehicle | |
| --- | --- | --- | --- | --- | --- |
| Metabolites | FC | Metabolites | FC | Metabolites | FC |
| L- (+) lactic acid | 0.684006 | galactosamine | 1.322575 | L-cysteine | 0.606202 |
|  |  | 4-aminobenzoic acid | 1.077937 | L- (+) lactic acid | 0.330828 |
|  |  | L- (+) lactic acid | 0.719885 | pyruvic acid | 0.318794 |
|  |  | pyruvic acid | 0.681692 | 3-methyl-2-oxobutanoic acid | 0.121284 |
|  |  | 3-methyl-2-oxobutanoic acid | 0.373379 |  |  |

**Table S9.** Differences of extracellular metabolites in 16-HBE cells stimulated with different concentrations of CSE for 24 hours.

| 3% CSE vs vehicle | | 6% CSE vs vehicle | | 10% CSE vs vehicle | |
| --- | --- | --- | --- | --- | --- |
| Metabolites | FC | Metabolites | FC | Metabolites | FC |
| L-threonine | 1.837121 | phosphoric acid | 2.001807 | L-cystine | 5.066695 |
| DL-isoleucine | 1.277925 | L-glutamine | 1.971796 | D-mannose | 1.75092 |
| L-serine | 0.443012 | eicosane | 0.762924 | 4-aminobenzoic acid | 1.340182 |
| L- (+) lactic acid | 0.371576 | 1-stearoyl-rac-glycerol | 0.743177 | L-serine | 1.319258 |
|  |  | stearic acid | 0.706537 | stearic acid | 0.810068 |
|  |  | palmitic acid | 0.702805 | L- (+) lactic acid | 0.305367 |

**Table S10.** Differences of extracellular metabolites in 16-HBE cells stimulated with different concentrations of CSE for 48 hours.

| 3% CSE vs vehicle | | 6% CSE vs vehicle | | 10% CSE vs vehicle | |
| --- | --- | --- | --- | --- | --- |
| Metabolites | FC | Metabolites | FC | Metabolites | FC |
| citrulline | 2.032376 | L-cystine | 12.13253 | L-cystine | 12.36094 |
| aspartic acid | 1.715771 | aspartic acid | 1.910998 | indole-3-carbaldehyde | 2.262061 |
| 4-aminobenzoic acid | 1.271879 | 4-aminobenzoic acid | 1.717127 | 4-aminobenzoic acid | 1.907131 |
| L-glutamine | 0.623219 | indole-3-carbaldehyde | 1.483222 | L-glutamic acid | 0.73483 |
| 3- (4-hydroxyphenyl) lactic acid | 0.290919 | pyruvic acid | 0.56299 | L-glutamine | 0.6169 |
|  |  | L-cysteine | 0.470656 | L-cysteine | 0.373849 |
|  |  |  |  | pyruvic acid | 0.164083 |
|  |  |  |  | L- (+) lactic acid | 0.158442 |

**Table S11.** Differences in metabolites after CSE stimulation in 16-HBE cells following GPT2 knockdown.

| cell*^NC^* +CSE vs cell*^NC^* | | cell*^siGPT2^* vs cell*^NC^* | | cell*^siGPT2^*+CSE vs cell*^siGPT2^* | |
| --- | --- | --- | --- | --- | --- |
| Metabolites | FC | Metabolites | FC | Metabolites | FC |
| D-mannose | 6.25758 | D-mannose | 11.31906 | phosphoric acid | 0.797033 |
| D-glucose | 4.861229 | D-glucose | 8.479273 | xanthine | 0.763699 |
| citric acid | 1.967171 | L- sorbose | 2.548629 | N, N-dimethylglycine | 0.756306 |
| cysteinylglycine | 1.357313 | glycerol 1-phosphate | 2.020701 | 1,3-propanediol | 0.752857 |
| succinic acid | 1.207686 | phosphoenolpyruvic acid | 1.87703 | D-malic acid | 0.74257 |
| L-glutamic acid | 1.100897 | 3-phosphoglycerate | 1.85689 | pelargonic acid | 0.721846 |
| 1,3-propanediol | 0.976474 | L-mimosine | 1.746421 | acetohydroxamic acid | 0.721745 |
| oxalic acid | 0.890141 | citramalic acid | 1.717271 | L-threonine | 0.70708 |
| palmitic acid | 0.884851 | N-ethylglycine | 1.572906 | succinic acid | 0.701969 |
| L- (+) lactic acid | 0.879521 | L-ornithine | 1.518705 | oxalic acid | 0.69655 |
| N-ethylglycine | 0.864664 | 1,5-anhydro-D-sorbitol | 1.406748 | 3-phosphoglycerate | 0.691435 |
| citrulline | 0.803526 | L- (+) lactic acid | 1.33616 | L-serine | 0.677742 |
| myristic acid | 0.777877 | L-glutamic acid | 1.319845 | citramalic acid | 0.670418 |
| L-lysine | 0.774658 | allo-inositol | 1.291064 | hexadecene | 0.658897 |
| L-tyrosine | 0.747048 | pelargonic acid | 1.273303 | stearic acid | 0.639006 |
| L-threonine | 0.744942 | hexadecene | 1.255518 | L- sorbose | 0.62831 |
| L-alanine | 0.743027 | L-tyrosine | 1.230871 | palmitic acid | 0.610739 |
| fumaric acid | 0.742129 | 1 monostearin | 1.225747 | citrulline | 0.597357 |
| L-mimosine | 0.727389 | L-lysine | 1.18322 | L-ornithine | 0.590648 |
| L-valine | 0.724107 | citrulline | 1.167233 | arachidic acid | 0.586728 |
| 1-methyl nicotinamide | 0.72168 | arachidic acid | 1.138276 | L-lysine | 0.57973 |
| elaidic acid | 0.70981 | phosphoric acid | 1.138137 | linoleic acid | 0.57394 |
| uracil | 0.700647 | aspartic acid | 1.124647 | fumaric acid | 0.547795 |
| glycerol 1-phosphate | 0.698177 | palmitoleic acid | 1.121863 | 1,5-anhydro-D-sorbitol | 0.543767 |
| L-norleucine | 0.694079 | 1-methyl nicotinamide | 1.120596 | myristic acid | 0.54245 |
| linoleic acid | 0.693892 | DL-isoleucine | 1.113117 | L-glutamic acid | 0.539994 |
| L-methionine | 0.680951 | stearic acid | 1.105099 | L-norleucine | 0.536425 |
| DL-isoleucine | 0.672638 | L-methionine | 1.099351 | L-methionine | 0.534309 |
| L-tryptophan | 0.668018 | glycine | 1.090128 | elaidic acid | 0.519993 |
| glycine | 0.648475 | oxalic acid | 1.088554 | L-valine | 0.518479 |
| O-phosphocolamine | 0.631717 | L-tryptophan | 1.08763 | L- (+) lactic acid | 0.492041 |
| L-ornithine | 0.630209 | palmitic acid | 1.085426 | DL-isoleucine | 0.482769 |
| pyrophosphate | 0.627675 | cholesterol | 1.064974 | N-ethylglycine | 0.48106 |
| L-cysteine | 0.627553 | L-valine | 1.064435 | L-tyrosine | 0.463478 |
| L-proline | 0.627231 | 1,3-propanediol | 0.901465 | L-alanine | 0.453818 |
| cholesterol | 0.579224 | fumaric acid | 0.901281 | inosine | 0.447775 |
| hypoxanthine | 0.576035 | hypoxanthine | 0.847673 | 1 monostearin | 0.44639 |
| L-asparagine | 0.531814 | N, N-dimethylglycine | 0.83832 | glycerol 1-phosphate | 0.418671 |
| hypotaurine | 0.50031 | pyruvic acid | 0.753594 | L-proline | 0.418244 |
| inosine | 0.491588 | L-alanine | 0.640894 | O-phosphocolamine | 0.415599 |
| palmitoleic acid | 0.473054 | inosine | 0.611646 | L-tryptophan | 0.39082 |
| allo-inositol | 0.383718 | cysteinylglycine | 0.474933 | cysteinylglycine | 0.385431 |
| aspartic acid | 0.326905 | ethanolamine | 0.175026 | cholesterol | 0.365361 |
|  |  |  |  | L-mimosine | 0.354773 |
|  |  |  |  | glycine | 0.352815 |
|  |  |  |  | 1-methyl nicotinamide | 0.347927 |
|  |  |  |  | L-cysteine | 0.344539 |
|  |  |  |  | hypotaurine | 0.340656 |
|  |  |  |  | palmitoleic acid | 0.316446 |
|  |  |  |  | uracil | 0.311093 |
|  |  |  |  | hypoxanthine | 0.307048 |
|  |  |  |  | L-asparagine | 0.22187 |
|  |  |  |  | allo-inositol | 0.216852 |
|  |  |  |  | aspartic acid | 0.206743 |
|  |  |  |  | pyrophosphate | 0.155835 |

**Table S12.** The top 30 up-regulated and top 30 down-regulated intracellular lipids in 16-HBE cells stimulated with different concentrations of CSE for 12 hours.

| 3% CSE vs vehicle | | 6% CSE vs vehicle | | 10% CSE vs vehicle | |
| --- | --- | --- | --- | --- | --- |
| Lipids | FC | Lipids | FC | Lipids | FC |
| TAG58:8-FA22:5 | 6.098825 | TAG51:1-FA17:0 | 5.332225 | TAG58:8-FA22:5 | 9.098665 |
| PC (18:0/18:3) | 1.151642 | TAG54:5-FA16:0 | 3.107493 | TAG51:1-FA17:0 | 5.171502 |
| TAG50:2-FA18:1 | 0.493836 | DAG (18:0/18:3) | 3.062571 | TAG52:5-FA22:5 | 3.135877 |
| TAG52:3-FA16:1 | 0.486729 | TAG50:1-FA20:1 | 2.882901 | TAG54:7-FA18:2 | 2.559864 |
| TAG55:7-FA22:6 | 0.484185 | TAG58:7-FA22:6 | 2.059027 | CE (16:0) | 2.079581 |
| TAG48:2-FA16:1 | 0.466606 | PE (O-16:0/18:3) | 1.917706 | PE (O-18:0/18:3) | 2.062375 |
| TAG52:5-FA14:0 | 0.462378 | PC (18:0/18:3) | 1.746326 | PE (O-16:0/18:3) | 2.031017 |
| TAG48:3-FA16:1 | 0.459212 | PE (14:0/20:4) | 1.708424 | PE (O-18:0/20:1) | 1.912689 |
| TAG50:1-FA18:1 | 0.450123 | PE (O-18:0/18:3) | 1.689461 | PE (O-18:0/22:4) | 1.750538 |
| TAG46:2-FA18:1 | 0.398931 | PE (O-18:0/20:1) | 1.673058 | PC (18:0/18:3) | 1.730117 |
| DAG (16:1/22:6) | 0.397545 | PC (16:0/18:3) | 1.636888 | PE (O-18:0/22:5) | 1.721548 |
| TAG50:5-FA20:5 | 0.397485 | PC (18:1/18:3) | 1.527549 | SM (18:0) | 1.569952 |
| TAG46:2-FA16:0 | 0.387653 | LPC (18:3) | 1.520447 | SM (20:0) | 1.563412 |
| TAG50:3-FA16:1 | 0.374946 | PC (14:0/18:3) | 1.502962 | PE (O-16:0/20:1) | 1.547489 |
| TAG46:2-FA14:0 | 0.373573 | TAG50:1-FA16:0 | 1.451182 | PE (O-16:0/22:4) | 1.500515 |
| TAG48:1-FA16:1 | 0.365889 | PE (O-16:0/18:2) | 1.43088 | PE (O-18:0/22:6) | 1.445205 |
| TAG44:1-FA14:0 | 0.3623 | PE (O-16:0/20:1) | 1.392696 | TAG54:6-FA18:2 | 1.44143 |
| PE (14:0/20:2) | 0.340964 | PE (O-16:0/20:4) | 1.343677 | SM (18:1) | 1.406223 |
| TAG48:2-FA14:0 | 0.32754 | PE (O-18:0/22:6) | 1.328836 | LPC (18:3) | 1.395997 |
| TAG50:4-FA18:1 | 0.302602 | PE (O-18:0/20:4) | 1.295706 | PC (16:0/18:3) | 1.326679 |
| TAG54:7-FA20:5 | 0.281718 | PE (O-16:0/18:1) | 1.264161 | PC (18:1/18:3) | 1.324855 |
| TAG44:2-FA16:1 | 0.270697 | PE (16:0/18:2) | 1.20577 | PC (14:0/18:3) | 1.297796 |
| TAG51:2-FA17:0 | 0.257371 | PE (18:0/18:2) | 1.195889 | PE (O-16:0/20:4) | 1.289054 |
| DAG (18:0/18:2) | 0.253529 | LPC (18:2) | 1.161487 | PE (18:0/18:3) | 1.248873 |
| TAG54:5-FA22:4 | 0.235694 | PE (16:0/16:1) | 1.10294 | SM (20:1) | 1.226994 |
| TAG47:1-FA17:0 | 0.223383 | PC (18:0/18:2) | 1.094312 | PE (O-18:0/20:4) | 1.216578 |
| TAG56:7-FA20:5 | 0.221666 | LPC (18:0) | 1.088356 | TAG48:2-FA18:1 | 0.354422 |
| TAG60:11-FA22:5 | 0.214459 | PC (16:0/20:3) | 1.087145 | PC (16:0/16:0) | 0.344513 |
| DAG (18:0/22:6) | 0.206573 | PC (18:0/14:0) | 1.085811 | TAG50:4-FA18:1 | 0.342714 |
| TAG54:7-FA16:1 | 0.183181 | LPC (16:0) | 1.076217 | PE (18:2/20:2) | 0.340812 |
| TAG56:7-FA20:4 | 0.164781 | TAG54:3-FA18:1 | 0.682354 | TAG50:3-FA16:1 | 0.327225 |
| CE (24:0) | 0.05298 | PE (18:0/20:5) | 0.678781 | TAG52:2-FA16:1 | 0.318248 |
|  |  | TAG52:4-FA18:1 | 0.671371 | TAG52:3-FA16:1 | 0.315346 |
|  |  | CER (14:0) | 0.666217 | TAG46:2-FA18:1 | 0.313 |
|  |  | PE (P-18:1/16:0) | 0.660117 | PE (P-16:0/16:0) | 0.312915 |
|  |  | PE (P-16:0/20:5) | 0.658408 | PE (P-18:1/16:0) | 0.310441 |
|  |  | PE (P-18:0/20:5) | 0.653001 | TAG56:7-FA22:5 | 0.307172 |
|  |  | PE (18:2/20:5) | 0.649799 | TAG48:3-FA18:2 | 0.306693 |
|  |  | PE (P-16:0/16:0) | 0.648944 | DAG (18:0/22:6) | 0.297919 |
|  |  | PE (P-18:1/20:5) | 0.646401 | TAG44:1-FA18:1 | 0.259198 |
|  |  | TAG54:6-FA20:5 | 0.576675 | PE (14:0/22:6) | 0.243994 |
|  |  | PC (18:2/20:5) | 0.566076 | TAG50:3-FA18:1 | 0.228699 |
|  |  | TAG52:2-FA16:1 | 0.552319 | TAG54:8-FA18:2 | 0.214487 |
|  |  | SM (26:0) | 0.545232 | TAG54:3-FA20:1 | 0.199261 |
|  |  | TAG44:1-FA14:0 | 0.501789 | TAG52:7-FA20:5 | 0.189543 |
|  |  | TAG50:3-FA16:1 | 0.497435 | TAG46:2-FA14:0 | 0.180399 |
|  |  | TAG46:2-FA18:1 | 0.474763 | TAG52:3-FA20:1 | 0.17653 |
|  |  | TAG56:7-FA20:5 | 0.451026 | TAG48:3-FA16:1 | 0.161207 |
|  |  | PE (14:0/20:5) | 0.44341 | DAG (16:1/22:6) | 0.149042 |
|  |  | TAG44:2-FA16:1 | 0.433968 | TAG56:7-FA20:5 | 0.137069 |
|  |  | DAG (18:0/22:6) | 0.407169 | TAG48:3-FA18:3 | 0.118663 |
|  |  | TAG50:4-FA18:1 | 0.369567 | TAG54:5-FA22:4 | 0.116364 |
|  |  | TAG56:8-FA22:6 | 0.352113 | TAG56:5-FA22:4 | 0.093691 |
|  |  | TAG52:3-FA20:1 | 0.29323 | TAG46:2-FA16:0 | 0.092411 |
|  |  | TAG58:9-FA20:4 | 0.28107 | TAG45:1-FA18:1 | 0.091363 |
|  |  | CE (24:0) | 0.238698 | CE (24:0) | 0.089142 |
|  |  | TAG50:5-FA20:5 | 0.14109 |  |  |
|  |  | TAG54:8-FA18:2 | 0.126527 |  |  |
|  |  | TAG46:3-FA18:1 | 0.082934 |  |  |
|  |  | TAG52:7-FA20:5 | 0.060783 |  |  |

**Table S13.** The top 30 up-regulated and top 30 down-regulated intracellular lipids in 16-HBE cells stimulated with different concentrations of CSE for 24 hours.

| 3% CSE vs vehicle | | 6% CSE vs vehicle | | 10% CSE vs vehicle | |
| --- | --- | --- | --- | --- | --- |
| Lipids | FC | Lipids | FC | Lipids | FC |
| TAG54:6-FA20:4 | 8.245236 | TAG56:2-FA18:0 | 8.75282 | CE (22:6) | 8.306204 |
| TAG56:1-FA16:0 | 7.815921 | TAG53:1-FA18:1 | 7.703452 | PE (14:0/22:4) | 6.544233 |
| TAG46:3-FA16:0 | 5.286315 | TAG54:6-FA20:4 | 7.398111 | TAG48:0-FA18:0 | 5.366717 |
| PC (20:0/20:1) | 4.9656 | PE (14:0/22:4) | 7.039156 | TAG54:6-FA20:4 | 4.44853 |
| TAG54:0-FA18:0 | 3.877536 | PC (20:0/20:1) | 5.57159 | PC (20:0/20:3) | 2.961248 |
| TAG45:0-FA16:0 | 3.732502 | TAG48:0-FA18:0 | 5.536983 | PE (14:0/22:6) | 2.92362 |
| TAG56:4-FA18:0 | 3.036612 | TAG49:2-FA18:1 | 4.817727 | TAG58:7-FA18:0 | 2.697212 |
| TAG56:6-FA16:0 | 2.290523 | TAG55:1-FA16:0 | 4.464095 | TAG42:0-FA14:0 | 2.522528 |
| TAG56:3-FA18:1 | 1.913137 | PC (20:0/20:3) | 4.402572 | PE (O-18:0/22:5) | 2.186895 |
| TAG52:0-FA18:0 | 1.8801 | TAG55:3-FA18:1 | 3.424464 | PE (O-18:0/22:4) | 2.134485 |
| TAG56:5-FA16:0 | 1.816144 | TAG52:1-FA18:0 | 2.862276 | PC (18:0/18:3) | 1.814412 |
| TAG54:2-FA18:1 | 1.779075 | TAG54:3-FA18:2 | 2.569884 | PE (18:0/18:3) | 1.712317 |
| PC (20:0/20:4) | 1.71579 | TAG48:4-FA16:1 | 2.480963 | PC (14:0/18:3) | 1.683552 |
| PC (20:0/18:1) | 1.681981 | TAG50:2-FA18:2 | 2.474269 | SM (20:0) | 1.659601 |
| PC (18:0/20:1) | 1.637056 | CE (20:5) | 2.307147 | PE (O-18:0/22:6) | 1.603498 |
| PC (18:0/18:0) | 1.61296 | TAG54:0-FA18:0 | 2.104322 | PE (O-16:0/18:3) | 1.586368 |
| PC (18:0/18:1) | 1.43695 | PC (18:0/18:3) | 1.984017 | PE (O-18:0/20:4) | 1.421786 |
| PE (P-16:0/20:1) | 1.371136 | TAG56:5-FA20:4 | 1.948243 | PE (16:0/22:4) | 1.407332 |
| PE (16:0/22:4) | 1.368902 | PC (18:0/18:0) | 1.893751 | SM (18:0) | 1.371931 |
| PC (18:1/20:1) | 1.365026 | PC (20:0/18:1) | 1.817149 | PC (16:0/18:3) | 1.336747 |
| PC (18:0/22:5) | 1.332133 | LPC (20:0) | 1.741402 | PE (P-18:0/18:3) | 1.327765 |
| PC (16:0/18:0) | 1.326637 | PC (16:0/18:3) | 1.617192 | PC (18:0/18:0) | 1.278936 |
| PC (18:0/22:6) | 1.314609 | PE (O-18:0/22:5) | 1.608244 | LPC (20:0) | 1.260319 |
| PC (18:0/14:0) | 1.307691 | PE (18:0/18:3) | 1.591934 | PC (18:0/22:6) | 1.212014 |
| PC (16:0/18:1) | 1.302002 | TAG54:2-FA18:1 | 1.563834 | PE (O-16:0/20:4) | 1.201842 |
| PC (18:1/18:3) | 1.293305 | TAG50:0-FA18:0 | 1.553776 | PC (18:0/18:2) | 1.150268 |
| PE (18:0/22:5) | 1.285617 | TAG47:2-FA16:1 | 1.539081 | SM (24:1) | 0.326147 |
| PC (16:0/20:1) | 1.282213 | PC (18:0/22:5) | 1.517752 | TAG50:2-FA14:0 | 0.322884 |
| PE (O-18:0/22:5) | 1.276782 | PC (18:0/20:1) | 1.50053 | DAG (16:0/18:2) | 0.308452 |
| PC (18:0/18:3) | 1.27425 | PC (18:0/18:1) | 1.478114 | PE (P-16:0/16:0) | 0.308177 |
| PC (14:0/20:3) | 0.809115 | PE (P-18:2/20:4) | 0.607074 | PC (18:2/16:1) | 0.305009 |
| PC (16:0/16:0) | 0.803336 | PE (16:0/14:0) | 0.590611 | PE (P-18:1/16:0) | 0.303549 |
| SM (22:1) | 0.793123 | SM (14:0) | 0.569856 | PC (16:0/16:0) | 0.303173 |
| LPC (22:6) | 0.787581 | PC (16:0/16:0) | 0.567091 | TAG51:3-FA16:1 | 0.295964 |
| PC (18:1/20:5) | 0.781983 | PC (16:1/18:1) | 0.566211 | SM (22:1) | 0.294912 |
| LPC (20:2) | 0.77847 | TAG44:0-FA14:0 | 0.564621 | DAG (16:1/18:1) | 0.29076 |
| SM (14:0) | 0.765257 | PE (16:0/16:0) | 0.556877 | PE (O-16:0/16:0) | 0.288595 |
| SM (24:1) | 0.757657 | PC (16:1/18:2) | 0.543954 | PC (16:1/18:1) | 0.284143 |
| LPC (20:4) | 0.756136 | SM (22:1) | 0.538468 | TAG50:3-FA16:1 | 0.267856 |
| SM (18:1) | 0.752123 | PE (O-16:0/16:1) | 0.535935 | DAG (16:0/18:1) | 0.26269 |
| TAG52:2-FA16:0 | 0.737685 | PE (P-18:1/16:1) | 0.532742 | TAG52:3-FA16:1 | 0.258041 |
| PE (P-18:0/16:0) | 0.714018 | TAG56:5-FA16:0 | 0.508508 | TAG54:6-FA18:3 | 0.25578 |
| PC (18:2/16:1) | 0.703496 | PE (14:0/16:1) | 0.505935 | TAG54:3-FA18:1 | 0.247335 |
| PE (P-18:1/16:0) | 0.698279 | SM (24:1) | 0.504225 | TAG58:3-FA18:1 | 0.240954 |
| PE (O-16:0/16:0) | 0.683319 | SM (26:1) | 0.483534 | TAG48:2-FA18:1 | 0.235971 |
| PC (16:1/18:2) | 0.681415 | DAG (16:1/18:1) | 0.480745 | TAG54:2-FA20:1 | 0.225927 |
| PC (18:2/20:4) | 0.673246 | PE (P-18:0/16:0) | 0.473189 | TAG49:2-FA16:0 | 0.192567 |
| PE (O-16:0/20:2) | 0.660223 | TAG49:2-FA16:0 | 0.468521 | DAG (16:1/20:2) | 0.172214 |
| SM (26:1) | 0.656681 | PE (14:0/20:4) | 0.460785 | TAG54:7-FA16:1 | 0.167136 |
| PE (P-16:0/16:0) | 0.637728 | TAG48:2-FA16:1 | 0.455193 | TAG51:2-FA16:0 | 0.158095 |
| PC (14:0/20:2) | 0.5912 | TAG49:3-FA16:0 | 0.451579 | DAG (18:1/20:2) | 0.151621 |
| TAG54:5-FA18:1 | 0.575714 | PE (P-18:1/20:2) | 0.442607 | DAG (18:1/20:4) | 0.14543 |
| DAG (14:0/20:4) | 0.536718 | PC (18:2/16:1) | 0.406162 | DAG (14:0/16:1) | 0.128505 |
| CE (22:1) | 0.489839 | PE (O-16:0/20:2) | 0.398452 | PE (14:0/20:2) | 0.108494 |
| TAG47:2-FA16:1 | 0.481232 | PE (O-16:0/16:0) | 0.392549 | TAG49:3-FA16:0 | 0.086593 |
| TAG51:3-FA16:1 | 0.426348 | PE (P-18:1/16:0) | 0.360754 | DAG (18:2/20:3) | 0.083208 |
| TAG56:7-FA20:5 | 0.247239 | PE (P-16:0/16:0) | 0.34878 |  |  |
| TAG58:7-FA16:0 | 0.245503 | CE (22:1) | 0.288378 |  |  |
| TAG58:7-FA22:6 | 0.17658 | TAG50:4-FA20:3 | 0.222657 |  |  |
| TAG54:6-FA18:3 | 0.140659 | DAG (16:1/20:4) | 0.098487 |  |  |

**Table S14.** The top 30 up-regulated and top 30 down-regulated intracellular lipids in 16-HBE cells stimulated with different concentrations of CSE for 48 hours.

| 3% CSE vs vehicle | | 6% CSE vs vehicle | | 10% CSE vs vehicle | |
| --- | --- | --- | --- | --- | --- |
| Lipids | FC | Lipids | FC | Lipids | FC |
| LPE (20:5) | 2.367042 | TAG56:3-FA20:1 | 143.9156 | TAG56:7-FA20:4 | 96.53228 |
| TAG42:0-FA16:0 | 2.099477 | TAG58:6-FA18:0 | 140.4126 | TAG58:6-FA18:0 | 90.43855 |
| PE (P-18:0/20:5) | 2.035745 | TAG56:3-FA18:0 | 133.5618 | TAG56:7-FA18:2 | 84.49773 |
| PC (20:0/22:4) | 1.980086 | TAG50:3-FA18:0 | 108.9039 | TAG52:2-FA18:2 | 78.11925 |
| PC (20:0/20:1) | 1.952125 | TAG56:7-FA20:4 | 103.6721 | TAG51:3-FA18:2 | 69.25619 |
| PC (20:0/20:3) | 1.83649 | TAG56:4-FA20:2 | 99.38027 | TAG58:7-FA18:0 | 66.53079 |
| TAG50:1-FA18:1 | 1.54815 | TAG55:3-FA18:1 | 96.62427 | TAG56:6-FA20:5 | 64.55039 |
| PC (18:0/18:0) | 1.538324 | TAG58:5-FA18:1 | 95.72627 | TAG56:6-FA22:6 | 51.64848 |
| PC (20:0/18:1) | 1.508605 | TAG56:7-FA18:2 | 95.29797 | TAG53:3-FA18:2 | 50.5934 |
| PC (18:0/20:1) | 1.440609 | TAG56:3-FA20:2 | 93.80091 | TAG50:2-FA18:2 | 48.11857 |
| PC (18:0/18:1) | 1.423252 | TAG51:3-FA18:2 | 93.06004 | TAG52:4-FA18:3 | 43.7279 |
| PE (P-18:0/20:3) | 1.418977 | TAG58:7-FA18:0 | 80.75054 | TAG54:6-FA22:6 | 40.63219 |
| PE (P-18:0/18:2) | 1.359163 | TAG52:3-FA18:0 | 79.37568 | TAG42:1-FA18:1 | 36.05414 |
| PC (16:0/18:1) | 1.260572 | TAG58:6-FA18:1 | 78.70319 | TAG54:3-FA18:0 | 35.39809 |
| PC (18:1/20:1) | 1.2593 | TAG48:3-FA16:0 | 75.52018 | TAG58:8-FA18:2 | 35.24456 |
| PE (18:0/18:2) | 1.245141 | TAG48:2-FA18:0 | 74.46578 | TAG56:3-FA18:0 | 34.6887 |
| PE (18:0/18:3) | 1.159464 | TAG56:7-FA20:3 | 69.83172 | TAG52:3-FA18:0 | 33.78508 |
| PC (16:0/18:0) | 1.159421 | TAG54:3-FA18:0 | 68.90942 | TAG56:9-FA20:4 | 33.7283 |
| PE (P-18:0/20:4) | 1.120401 | TAG58:8-FA18:2 | 65.87602 | TAG48:3-FA16:0 | 32.52615 |
| PE (P-16:0/18:2) | 1.099822 | TAG56:6-FA20:5 | 61.03561 | TAG56:6-FA18:2 | 31.64908 |
| SM (18:0) | 1.083199 | TAG56:6-FA18:2 | 59.65027 | TAG58:9-FA18:1 | 31.52629 |
| SM (22:1) | 0.45578 | TAG52:2-FA18:2 | 59.16375 | TAG56:9-FA20:5 | 30.73912 |
| PE (P-18:1/16:1) | 0.452233 | TAG56:6-FA22:6 | 58.11558 | TAG58:6-FA18:1 | 29.26782 |
| PC (18:2/20:2) | 0.443722 | TAG56:4-FA20:3 | 56.24836 | TAG40:0-FA16:0 | 27.11146 |
| PE (P-18:1/16:0) | 0.436677 | TAG52:4-FA18:3 | 55.5059 | TAG53:5-FA20:4 | 27.05703 |
| PE (16:0/16:0) | 0.435358 | TAG56:2-FA20:1 | 55.2739 | TAG54:3-FA18:2 | 27.03688 |
| CER (14:0) | 0.429675 | TAG53:1-FA18:1 | 53.95601 | TAG56:7-FA20:3 | 25.99787 |
| PE (14:0/20:2) | 0.429395 | TAG56:2-FA20:0 | 52.72412 | TAG58:9-FA22:5 | 25.23531 |
| SM (26:1) | 0.42082 | TAG56:5-FA18:0 | 52.1892 | TAG56:6-FA18:0 | 25.0663 |
| PE (16:0/14:0) | 0.420156 | TAG54:2-FA18:0 | 51.4802 | TAG56:5-FA18:0 | 24.34325 |
| TAG46:0-FA14:0 | 0.41712 | PC (18:1/22:6) | 0.843164 | PC (14:0/18:1) | 0.041794 |
| PE (P-16:0/16:0) | 0.413305 | PC (16:0/16:1) | 0.840852 | LPC (14:0) | 0.039778 |
| PE (O-16:0/22:4) | 0.408087 | PC (14:0/14:0) | 0.824404 | PC (18:1/20:1) | 0.039452 |
| DAG (18:1/22:5) | 0.40789 | PE (P-18:1/22:6) | 0.818378 | PE (P-18:1/22:5) | 0.039352 |
| TAG58:8-FA22:5 | 0.40569 | PE (P-18:1/20:1) | 0.805604 | PC (18:1/20:2) | 0.03921 |
| PE (O-16:0/16:1) | 0.404703 | LPC (16:0) | 0.758207 | PE (P-18:1/22:4) | 0.038208 |
| PC (18:2/20:4) | 0.394786 | PC (18:0/20:0) | 0.757048 | PE (P-16:0/16:1) | 0.036652 |
| PE (P-18:1/22:4) | 0.393718 | PC (16:0/20:1) | 0.756438 | PE (18:1/20:2) | 0.036552 |
| PE (14:0/14:0)-H | 0.37832 | LPC (20:4) | 0.755647 | PE (P-16:0/20:1) | 0.036128 |
| PE (18:2/20:2) | 0.367355 | PE (P-18:1/16:1) | 0.7469 | PE (P-18:1/22:6) | 0.036078 |
| PE (O-16:0/16:0) | 0.362647 | LPC (22:5) | 0.735038 | PE (P-16:0/20:4) | 0.035887 |
| PE (14:0/16:1) | 0.334501 | PE (P-16:0/16:1) | 0.703661 | PE (18:0/20:1) | 0.035291 |
| PC (20:0/20:2) | 0.316456 | PC (14:0/20:1) | 0.66637 | PE (18:1/22:5) | 0.034831 |
| PE (14:0/22:5) | 0.315308 | PE (P-18:1/16:0) | 0.662814 | PE (16:0/16:1) | 0.031246 |
| TAG52:1-FA16:1 | 0.301885 | PE (P-16:0/22:4) | 0.618204 | PE (P-18:1/20:1) | 0.030968 |
| TAG54:7-FA20:5 | 0.189486 | PE (18:1/20:4) | 0.6103 | PE (P-16:0/22:4) | 0.026845 |
| PE (O-16:0/18:3) | 0.18351 | PE (P-16:0/20:1) | 0.596259 | PE (18:1/20:1) | 0.025173 |
| TAG47:2-FA18:2 | 0.176446 | PE (O-16:0/22:6) | 0.578115 | PE (P-16:0/22:5) | 0.024712 |
| TAG58:3-FA18:1 | 0.155619 | PE (O-16:0/16:1) | 0.569401 | PE (18:1/18:1) | 0.023117 |
| TAG52:5-FA14:0 | 0.108167 | LPC (14:0) | 0.53664 | LPE (16:1) | 0.022649 |
| DAG (18:2/20:4) | 0.088032 | PE (P-16:0/22:6) | 0.508711 | PE (18:1/20:4) | 0.022582 |
|  |  | LPC (18:1) | 0.502752 | PC (14:0/20:1) | 0.019634 |
|  |  | LPC (20:1) | 0.465287 | PC (16:0/14:0) | 0.019217 |
|  |  | CER (16:0) | 0.43703 | PE (P-18:1/16:1) | 0.018647 |
|  |  | PE (O-16:0/20:1) | 0.431597 | PE (14:0/14:0)-H | 0.016751 |
|  |  | DAG (16:1/18:1) | 0.373763 | PE (P-16:0/22:6) | 0.015941 |
|  |  | LPC (16:1) | 0.341644 | PC (16:1/18:1) | 0.01586 |
|  |  | CER (14:0) | 0.272859 | PE (18:1/20:5) | 0.014878 |
|  |  |  |  | PE (14:0/16:1) | 0.010829 |
|  |  |  |  | PC (14:0/14:0) | 0.00564 |

**Table S15.** The top 30 up-regulated and top 30 down-regulated extracellular lipids in 16-HBE cells stimulated with different concentrations of CSE for 12 hours.

| 3% CSE vs vehicle | | 6% CSE vs vehicle | | 10% CSE vs vehicle | |
| --- | --- | --- | --- | --- | --- |
| Lipids | FC | Lipids | FC | Lipids | FC |
| PE (P-18:2/22:6) | 15.65761 | PE (14:0/14:0)-H | 35.45089 | PE (14:0/14:0)-H | 18.51101 |
| CE (20:2) | 8.172136 | SM (24:0) | 19.55993 | PE (P-18:1/20:2) | 6.442334 |
| PC (20:0/20:3) | 7.754793 | PE (P-18:2/22:6) | 14.88154 | PE (P-18:0/18:3) | 6.43793 |
| LPC (18:3) | 7.202381 | LPC (18:3) | 14.7943 | PE (P-18:2/22:6) | 6.367599 |
| TAG50:2-FA14:0 | 7.000332 | PE (O-16:0/22:4) | 9.266924 | PC (20:0/20:3) | 6.189759 |
| TAG49:2-FA17:0 | 6.358469 | PC (18:1/18:3) | 8.673847 | PC (14:0/20:3) | 5.418925 |
| PC (14:0/20:3) | 6.321935 | PC (20:0/20:3) | 8.293568 | PC (14:0/20:2) | 4.202132 |
| PC (14:0/20:2) | 4.899548 | PE (O-16:0/22:6) | 6.943059 | PE (P-18:0/18:0) | 4.15554 |
| TAG44:2-FA16:0 | 4.578592 | PC (14:0/20:3) | 6.737064 | PE (P-18:1/18:0) | 4.042298 |
| TAG52:1-FA18:0 | 4.51456 | TAG50:2-FA14:0 | 5.657823 | PE (O-16:0/16:0) | 3.939645 |
| DAG (14:0/18:3) | 4.427921 | PC (16:0/18:3) | 5.148154 | PE (P-16:0/18:0) | 3.921854 |
| PE (P-18:0/20:3) | 2.709692 | PE (O-16:0/22:5) | 5.032979 | PE (P-16:0/16:0) | 3.727292 |
| CE (22:6) | 2.655393 | PE (O-16:0/16:1) | 4.869925 | SM (18:1) | 3.480602 |
| PE (P-18:0/22:6) | 2.204615 | TAG42:0-FA16:0 | 4.767404 | PE (O-16:0/22:4) | 3.058832 |
| PE (18:0/22:4) | 2.135829 | TAG58:7-FA22:5 | 4.642882 | PE (P-18:0/16:0) | 2.296291 |
| PE (O-18:0/20:4) | 2.08508 | PE (O-18:0/22:4) | 4.389447 | PC (14:0/18:3) | 2.216417 |
| PC (14:0/18:3) | 1.827032 | PE (O-18:0/20:4) | 4.244812 | PC (16:0/18:3) | 1.981949 |
| PE (18:0/22:6) | 1.821402 | PE (O-18:0/22:5) | 4.149707 | SM (18:0) | 1.868717 |
| PC (16:0/18:3) | 1.813571 | PE (O-16:0/20:4) | 4.062964 | PC (18:0/20:0) | 1.722968 |
| LPC (18:2) | 1.651568 | PE (P-16:0/18:2) | 4.037174 | PE (P-18:0/18:1) | 1.649129 |
| LPC (22:5) | 1.605461 | PC (14:0/20:2) | 3.71534 | PE (18:0/22:4) | 1.579744 |
| LPC (20:3) | 1.54931 | TAG48:2-FA14:0 | 3.638538 | PE (P-18:1/18:1) | 1.495094 |
| LPC (20:4) | 1.336078 | PE (O-18:0/18:0) | 3.598961 | PE (P-16:0/18:1) | 1.414352 |
| LPC (16:1) | 1.303581 | PC (18:2/18:3) | 3.398421 | LPC (20:4) | 1.195674 |
| PC (18:1/18:1) | 0.93936 | PE (18:0/22:4) | 3.310841 | LPC (16:1) | 0.856289 |
| PC (18:0/20:4) | 0.911094 | PC (14:0/18:3) | 3.286566 | PC (16:0/20:4) | 0.85057 |
| PC (18:0/22:4) | 0.9054 | PC (18:0/18:3) | 3.103418 | PC (18:0/22:5) | 0.845768 |
| PC (16:0/18:1) | 0.89333 | PE (P-16:0/18:0) | 3.03579 | PC (16:0/18:0) | 0.829155 |
| PC (18:0/22:6) | 0.88477 | PE (16:0/20:3) | 2.924599 | PC (16:0/18:1) | 0.82496 |
| PC (18:0/18:2) | 0.866012 | PE (P-18:1/20:4) | 2.80846 | PC (18:0/22:6) | 0.821909 |
| PC (16:0/18:2) | 0.85159 | LPC (18:0) | 0.91901 | PC (18:0/18:0) | 0.819723 |
| PC (16:0/20:3) | 0.847735 | LPC (20:0) | 0.752672 | PC (18:0/18:2) | 0.815758 |
| PC (18:0/20:3) | 0.844622 | SM (22:0) | 0.483241 | PC (18:0/18:1) | 0.805762 |
| PC (16:0/16:0) | 0.844081 | DAG (16:0/18:3) | 0.313892 | PC (16:0/16:0) | 0.789162 |
| PC (18:0/18:1) | 0.836226 |  |  | PC (18:0/20:3) | 0.78435 |
| PC (16:0/22:6) | 0.830208 |  |  | PC (16:0/18:2) | 0.779069 |
| LPC (20:1) | 0.823582 |  |  | PE (18:1/20:4) | 0.773192 |
| PC (16:0/18:0) | 0.821016 |  |  | PC (16:0/22:5) | 0.760976 |
| PC (18:0/22:5) | 0.814695 |  |  | PC (16:0/20:3) | 0.76002 |
| LPC (18:0) | 0.802419 |  |  | PC (16:0/22:6) | 0.755819 |
| PC (16:0/22:4) | 0.794573 |  |  | PC (18:1/20:3) | 0.748244 |
| PC (16:0/22:5) | 0.791677 |  |  | PC (16:0/14:0) | 0.741195 |
| LPC (16:0) | 0.788029 |  |  | PE (18:0/20:3) | 0.669475 |
| LPC (20:0) | 0.653415 |  |  | PC (18:1/20:1) | 0.655953 |
| LPC (22:4) | 0.635905 |  |  | PC (14:0/14:0) | 0.589884 |
| SM (22:0) | 0.397839 |  |  | PE (18:1/18:1) | 0.589423 |
| PE (18:2/16:1) | 0.397021 |  |  | PC (18:0/14:0) | 0.569518 |
| SM (24:1) | 0.335389 |  |  | LPC (14:0) | 0.55827 |
| PC (18:2/20:4) | 0.281379 |  |  | LPC (20:1) | 0.518254 |
|  |  |  |  | LPC (18:1) | 0.511271 |
|  |  |  |  | LPC (22:4) | 0.505241 |
|  |  |  |  | LPC (18:0) | 0.48702 |
|  |  |  |  | LPC (16:0) | 0.469958 |
|  |  |  |  | LPC (20:0) | 0.439215 |

**Table S16.** The top 30 up-regulated and top 30 down-regulated extracellular lipids in 16-HBE cells stimulated with different concentrations of CSE for 24 hours.

| 3% CSE vs vehicle | | 6% CSE vs vehicle | | 10% CSE vs vehicle | |
| --- | --- | --- | --- | --- | --- |
| Lipids | FC | Lipids | FC | Lipids | FC |
| TAG52:1-FA20:1 | 5.825675 | PE (O-16:0/20:3) | 27.37471 | PE (O-18:0/20:5) | 631.4673 |
| TAG50:3-FA18:2 | 5.309776 | PE (O-18:0/20:3) | 24.1835 | LPC (20:4) | 575.3651 |
| PE (P-18:1/20:1) | 4.949173 | PE (O-18:0/22:6) | 19.95263 | PE (O-18:0/20:1) | 351.6454 |
| CE (20:2) | 4.324707 | PE (O-18:0/22:5) | 14.5027 | PE (O-18:0/20:3) | 229.9074 |
| PE (O-18:0/22:6) | 3.69795 | PE (O-16:0/20:4) | 12.72546 | LPC (18:2) | 197.2843 |
| PE (O-16:0/20:4) | 2.462481 | PE (O-16:0/22:5) | 12.14219 | PE (O-18:0/18:1) | 179.7871 |
| PE (16:0/16:0) | 2.333951 | PC (18:1/18:3) | 11.96473 | PE (O-18:0/20:2) | 177.9839 |
| PE (O-18:0/22:5) | 2.287723 | TAG54:3-FA20:2 | 11.66727 | PE (O-18:0/18:0) | 173.302 |
| PE (O-16:0/22:5) | 1.962327 | PE (O-18:0/22:4) | 11.40215 | PE (O-18:0/18:2) | 167.7168 |
| TAG51:2-FA18:1 | 1.896796 | PC (16:0/18:3) | 10.97743 | PC (18:2/20:5) | 155.7926 |
| SM (18:1) | 1.784025 | PE (P-18:0/18:3) | 10.05212 | PE (16:0/18:2) | 130.7568 |
| PE (O-18:0/20:4) | 1.774789 | PE (O-18:0/20:4) | 9.800626 | PE (18:2/18:2) | 107.8642 |
| PC (16:0/18:3) | 1.772002 | PE (O-16:0/22:6) | 9.066682 | LPC (20:2) | 105.3016 |
| SM (18:0) | 1.765813 | PC (18:2/18:3) | 8.955536 | PE (O-16:0/18:2) | 98.70357 |
| LPC (22:6) | 1.642724 | PE (P-18:1/18:0) | 8.811104 | PE (O-18:0/16:0) | 85.38522 |
| PC (18:0/22:4) | 1.381088 | PE (O-16:0/22:4) | 8.55361 | LPC (18:3) | 60.76163 |
| PC (16:0/20:2) | 1.371184 | PE (O-18:0/18:3) | 7.979163 | PE (18:2/16:1) | 58.14365 |
| PC (18:0/16:1) | 1.363194 | PE (O-16:0/18:0) | 7.439384 | PE (O-18:0/20:4) | 55.26062 |
| PC (18:1/20:1) | 1.358659 | PC (14:0/18:3) | 7.235797 | PE (16:0/20:5) | 53.27941 |
| PC (16:0/22:4) | 1.315454 | PC (18:0/18:3) | 7.230775 | LPC (20:3) | 50.45502 |
| PE (18:0/20:4) | 1.306113 | PE (18:1/18:3) | 6.251555 | PE (18:0/20:5) | 48.67618 |
| PC (18:0/18:2) | 1.224762 | PE (O-18:0/16:1) | 5.983246 | LPC (18:1) | 44.47031 |
| PC (16:0/18:2) | 1.215729 | PE (O-16:0/18:1) | 5.89152 | PE (18:1/20:5) | 39.09634 |
| PC (18:0/18:0) | 1.209459 | TAG48:2-FA16:0 | 5.038895 | PE (P-18:0/20:1) | 37.74319 |
| PC (18:0/20:2) | 1.174935 | SM (20:1) | 4.920776 | PE (P-18:0/18:2) | 36.02996 |
| PC (16:0/18:1) | 1.173178 | PE (P-16:0/16:0) | 4.864567 | PE (O-18:0/18:3) | 33.21044 |
| PC (18:0/22:5) | 1.170282 | PC (16:1/18:2) | 4.851765 | PE (O-16:0/20:3) | 25.87125 |
| PC (18:0/18:1) | 1.160189 | SM (20:0) | 4.832543 | PE (16:0/20:1) | 25.77494 |
| PC (18:0/22:6) | 1.152668 | PE (16:0/16:0) | 4.524839 | PE (16:0/16:0) | 24.4427 |
| PC (16:0/18:0) | 1.149693 | PE (P-14:0/18:1) | 4.479979 | PC (18:2/20:4) | 24.40616 |
| LPC (16:0) | 0.909671 | LPC (20:0) | 0.793519 | PC (14:0/18:1) | 0.892396 |
| PE (18:1/18:1) | 0.898865 | LPC (18:0) | 0.760629 | PC (16:0/14:0) | 0.839258 |
| LPC (18:0) | 0.764837 | LPC (20:1) | 0.721625 | PE (P-18:1/20:3) | 0.680136 |
| LPC (20:1) | 0.687658 | TAG51:2-FA16:0 | 0.205442 | TAG46:0-FA14:0 | 0.643434 |
| PC (20:0/20:2) | 0.582247 | TAG46:2-FA18:2 | 0.132062 | PC (20:0/20:2) | 0.430041 |
| PE (P-16:0/18:0) | 0.546984 | DAG (14:0/18:2) | 0.072385 | DAG (14:0/18:2) | 0.403171 |
| PE (18:1/22:5) | 0.521315 |  |  | TAG51:2-FA16:0 | 0.34121 |
| TAG49:3-FA16:1 | 0.300292 |  |  | PC (14:0/14:0) | 0.330345 |
| DAG (14:0/18:2) | 0.232021 |  |  | TAG56:7-FA22:5 | 0.228352 |
| CE (18:2) | 0.220251 |  |  | TAG52:6-FA18:3 | 0.198099 |
| TAG50:0-FA14:0 | 0.192682 |  |  | TAG54:2-FA18:0 | 0.076955 |

**Table S17.** The top 30 up-regulated and top 30 down-regulated extracellular lipids in 16-HBE cells stimulated with different concentrations of CSE for 48 hours.

| 3% CSE vs vehicle | | 6% CSE vs vehicle | | 10% CSE vs vehicle | |
| --- | --- | --- | --- | --- | --- |
| Lipids | FC | Lipids | FC | Lipids | FC |
| TAG47:2-FA18:2 | 18.51696 | DAG (16:0/18:3) | 16.14405 | PE (O-18:0/18:0) | 14.11212 |
| TAG50:3-FA14:0 | 8.930105 | PE (O-18:0/18:0) | 12.79317 | PE (O-16:0/18:0) | 11.70261 |
| PE (18:2/22:6) | 7.631288 | PE (O-16:0/18:0) | 9.866352 | PE (O-16:0/16:0) | 10.35379 |
| TAG50:4-FA18:2 | 6.282077 | PE (O-16:0/20:3) | 8.800463 | PE (P-18:0/18:0) | 9.840718 |
| TAG54:4-FA20:4 | 5.03843 | PC (18:0/18:3) | 7.02002 | PE (P-16:0/18:0) | 9.801393 |
| PE (O-16:0/18:2) | 4.795635 | PE (O-18:0/22:5) | 6.285374 | PE (P-16:0/16:0) | 9.124961 |
| TAG47:0-FA16:0 | 3.84171 | PC (16:0/18:3) | 6.260985 | PE (P-18:1/18:0) | 8.135695 |
| TAG54:6-FA18:2 | 3.166389 | PE (O-16:0/18:2) | 6.205888 | TAG54:6-FA20:4 | 6.279273 |
| PE (O-18:0/22:5) | 3.136986 | PE (O-16:0/16:0) | 6.162517 | PE (O-16:0/18:2) | 6.171846 |
| TAG51:2-FA18:2 | 3.10656 | PE (P-18:0/18:0) | 5.395256 | PE (16:0/16:0) | 6.063863 |
| PE (P-16:0/18:3) | 2.932857 | PC (18:1/18:3) | 5.191238 | PE (P-18:0/16:0) | 5.84127 |
| TAG44:0-FA16:0 | 2.847361 | TAG54:7-FA22:6 | 5.052652 | PE (O-18:0/16:0) | 5.300703 |
| PC (16:0/18:3) | 2.655029 | PE (O-18:0/20:4) | 4.747901 | PC (16:0/18:3) | 5.063812 |
| PC (18:0/18:3) | 2.640064 | PE (P-16:0/18:0) | 4.565749 | PC (18:2/20:4) | 4.442648 |
| PE (P-18:1/18:2) | 2.353643 | TAG44:0-FA16:0 | 4.47769 | TAG50:1-FA16:0 | 4.318901 |
| TAG52:4-FA18:1 | 2.208365 | PE (O-18:0/20:3) | 4.255999 | PE (P-16:0/18:3) | 3.793423 |
| PE (18:0/22:4) | 2.167354 | TAG52:3-FA18:2 | 4.215037 | TAG50:2-FA16:0 | 3.389882 |
| PE (P-18:0/18:2) | 2.07206 | PE (O-18:0/16:0) | 4.211392 | PC (18:0/18:3) | 3.309347 |
| TAG50:1-FA16:0 | 2.002605 | PE (P-16:0/18:3) | 4.143742 | TAG47:2-FA16:1 | 3.300295 |
| PE (18:0/20:3) | 1.99225 | PE (O-16:0/20:4) | 4.131088 | TAG52:2-FA18:0 | 3.151601 |
| PC (18:0/16:1) | 1.983254 | PE (O-16:0/22:4) | 3.861008 | TAG49:2-FA18:1 | 3.104423 |
| PE (P-16:0/18:2) | 1.952609 | TAG50:1-FA16:0 | 3.815116 | DAG (16:0/18:1) | 2.743538 |
| PE (P-18:0/20:1) | 1.946296 | PE (P-16:0/16:0) | 3.258217 | PE (P-18:1/16:0) | 2.694463 |
| PE (P-18:0/22:5) | 1.944143 | PE (P-18:1/18:0) | 3.189444 | PC (18:2/16:1) | 2.687945 |
| PE (P-18:0/16:1) | 1.923956 | PC (18:2/16:1) | 3.086909 | TAG52:4-FA18:1 | 2.521832 |
| PC (20:0/22:6) | 1.857052 | PE (O-16:0/22:6) | 3.084766 | PC (16:1/18:2) | 2.486621 |
| PE (P-18:0/20:4) | 1.825045 | PE (P-18:0/16:0) | 2.939084 | PC (18:0/18:0) | 2.401781 |
| PE (18:0/20:4) | 1.811353 | TAG50:3-FA16:1 | 2.888813 | PE (O-16:0/18:1) | 2.310845 |
| PE (P-16:0/20:1) | 1.782606 | PE (18:0/18:3) | 2.888206 | TAG44:1-FA16:0 | 2.253108 |
| PE (18:0/22:5) | 1.761372 | TAG48:2-FA16:0 | 2.850075 | PC (16:0/20:5) | 2.185696 |
| LPC (18:0) | 0.866072 | PC (18:0/22:6) | 0.768257 | PE (P-16:0/22:6) | 0.626777 |
| LPC (16:1) | 0.780206 | PC (14:0/18:1) | 0.746883 | PE (P-18:1/22:4) | 0.587822 |
| PC (14:0/20:3) | 0.772634 | PC (16:0/20:2) | 0.71973 | PE (P-18:1/20:4) | 0.586132 |
| LPC (20:4) | 0.619912 | PE (P-16:0/20:4) | 0.693139 | PC (16:0/14:0) | 0.57117 |
| LPC (20:1) | 0.543643 | PE (P-16:0/22:6) | 0.659954 | PE (18:0/18:1) | 0.553323 |
| TAG52:4-FA18:2 | 0.531916 | PE (16:0/18:1) | 0.626908 | PE (18:1/20:4) | 0.541519 |
| TAG52:3-FA18:1 | 0.494743 | LPC (20:0) | 0.584373 | PE (16:0/20:3) | 0.540756 |
|  |  | PE (P-16:0/22:5) | 0.559766 | PE (P-18:0/20:4) | 0.524302 |
|  |  | PC (14:0/20:3) | 0.559533 | PE (16:0/18:1) | 0.52044 |
|  |  | PE (18:1/22:5) | 0.543532 | PE (P-16:0/20:4) | 0.481909 |
|  |  | PE (18:1/18:2) | 0.52971 | PE (P-16:0/22:5) | 0.466132 |
|  |  | PE (18:1/20:4) | 0.522003 | PE (P-18:1/22:5) | 0.464755 |
|  |  | PC (18:1/22:4) | 0.490131 | TAG49:2-FA16:1 | 0.450069 |
|  |  | LPC (16:1) | 0.486614 | TAG54:5-FA18:2 | 0.447166 |
|  |  | LPC (16:0) | 0.441377 | LPC (16:1) | 0.432858 |
|  |  | LPC (18:1) | 0.436795 | PE (18:1/18:2) | 0.403395 |
|  |  | PE (P-16:0/20:5) | 0.401937 | PE (18:1/18:1) | 0.354189 |
|  |  | PE (P-16:0/20:1) | 0.398108 | PE (P-18:0/22:4) | 0.351935 |
|  |  | PE (18:1/18:1) | 0.389218 | PE (P-16:0/20:5) | 0.33818 |
|  |  | SM (22:1) | 0.363893 | LPC (18:1) | 0.326427 |
|  |  | PC (16:0/14:0) | 0.363007 | LPC (16:0) | 0.306927 |
|  |  | SM (22:0) | 0.357978 | LPC (20:0) | 0.300778 |
|  |  | LPC (18:0) | 0.351535 | TAG42:1-FA16:1 | 0.257498 |
|  |  | SM (24:0) | 0.347039 | LPC (18:0) | 0.23041 |
|  |  | LPC (14:0) | 0.291367 | LPC (14:0) | 0.216987 |
|  |  | LPC (20:1) | 0.27202 | PE (P-18:0/20:5) | 0.192296 |
|  |  | PC (14:0/14:0) | 0.245743 | LPC (20:2) | 0.19158 |
|  |  | PE (18:1/20:1) | 0.218111 | PC (14:0/14:0) | 0.129917 |
|  |  | PE (18:1/20:2) | 0.201551 | PE (P-18:1/20:1) | 0.072995 |
|  |  | TAG51:3-FA18:2 | 0.128665 | LPC (20:1) | 0.071856 |

**Table S18.** The top 30 up-regulated and top 30 down-regulated changed lipids after CSE stimulation in 16-HBE cells following GPT2 knockdown.

| cell*^NC^*+CSE vs cell*^NC^* | | cell*^siGPT2^* vs cell*^NC^* | | cell*^siGPT2^*+CSE vs cell*^siGPT2^* | |
| --- | --- | --- | --- | --- | --- |
| Lipids | FC | Lipids | FC | Lipids | FC |
| TAG54:8-FA20:4 | 2.822256 | TAG56:2-FA18:0 | 3.604155 | DAG (18:0/18:2) | 2.210413 |
| CE (20:3) | 2.796734 | TAG56:9-FA18:3 | 2.878741 | SM (20:0) | 1.123872 |
| TAG56:2-FA18:0 | 2.189724 | TAG53:5-FA20:4 | 1.837583 | SM (18:0) | 1.093938 |
| PE (O-18:0/18:3) | 1.743467 | PE (O-18:0/20:3) | 1.310842 | TAG52:2-FA20:1 | 0.399492 |
| PC (18:0/18:3) | 1.53484 | PE (P-14:0/18:0) | 0.533009 | TAG52:5-FA20:3 | 0.399301 |
| PE (18:0/18:3) | 1.483349 | CE (20:2) | 0.5232 | TAG54:3-FA18:3 | 0.398253 |
| PC (20:0/20:3) | 1.442642 | TAG54:5-FA20:4 | 0.521072 | TAG56:9-FA18:3 | 0.395687 |
| LPC (20:0) | 1.435282 | TAG51:2-FA18:1 | 0.507834 | TAG51:5-FA18:2 | 0.394389 |
| LPC (18:3) | 1.405027 | TAG48:2-FA18:2 | 0.505308 | TAG50:2-FA14:0 | 0.394236 |
| PC (14:0/18:3) | 1.345066 | TAG54:3-FA16:0 | 0.495298 | TAG56:8-FA18:1 | 0.386188 |
| TAG52:1-FA18:1 | 1.336224 | CE (22:5) | 0.481807 | TAG50:2-FA18:0 | 0.381209 |
| PC (18:0/22:5) | 1.289144 | CE (16:0) | 0.478697 | TAG51:2-FA16:0 | 0.380126 |
| PC (20:0/22:5) | 1.284507 | TAG54:4-FA18:2 | 0.476801 | TAG48:2-FA18:1 | 0.380015 |
| PC (18:0/22:6) | 1.233551 | PC (20:0/20:1) | 0.470335 | TAG56:3-FA20:0 | 0.370415 |
| PC (18:2/16:1) | 0.389885 | TAG52:3-FA18:1 | 0.46828 | TAG56:7-FA18:3 | 0.36772 |
| PE (14:0/16:1) | 0.380423 | TAG54:1-FA20:1 | 0.461588 | TAG52:5-FA18:1 | 0.3611 |
| TAG52:3-FA18:1 | 0.375635 | TAG56:5-FA20:4 | 0.443555 | TAG50:3-FA18:1 | 0.357861 |
| PE (16:0/20:5) | 0.374535 | TAG56:3-FA20:1 | 0.436353 | TAG52:2-FA20:0 | 0.352089 |
| TAG54:7-FA18:1 | 0.366768 | TAG54:6-FA18:1 | 0.434245 | TAG54:7-FA22:6 | 0.349966 |
| TAG46:2-FA16:1 | 0.365254 | TAG50:3-FA18:2 | 0.433032 | TAG53:5-FA20:4 | 0.349847 |
| TAG52:4-FA14:0 | 0.364645 | TAG50:3-FA16:0 | 0.409608 | TAG52:3-FA16:1 | 0.347081 |
| PC (16:1/18:1) | 0.356521 | TAG56:4-FA16:0 | 0.402199 | TAG58:8-FA22:5 | 0.333899 |
| TAG54:6-FA20:4 | 0.356452 | TAG40:0-FA16:0 | 0.383675 | TAG56:5-FA20:3 | 0.333481 |
| TAG50:5-FA18:3 | 0.356374 | TAG56:9-FA20:5 | 0.38149 | TAG58:5-FA18:1 | 0.319346 |
| TAG56:7-FA20:5 | 0.353892 | CE (14:0) | 0.376372 | TAG56:2-FA20:1 | 0.318924 |
| TAG52:4-FA16:1 | 0.349149 | TAG54:6-FA20:4 | 0.336621 | TAG48:3-FA14:0 | 0.311962 |
| PE (P-18:1/20:5) | 0.348605 | TAG49:0-FA18:0 | 0.330932 | TAG52:3-FA20:1 | 0.31131 |
| PE (P-18:1/16:0) | 0.335912 | PE (14:0/20:5) | 0.291374 | TAG56:2-FA20:0 | 0.296241 |
| TAG54:5-FA22:4 | 0.335157 | TAG60:10-FA22:5 | 0.28705 | TAG52:3-FA18:0 | 0.293718 |
| TAG54:7-FA20:5 | 0.327194 | TAG54:6-FA20:5 | 0.273236 | TAG54:4-FA20:2 | 0.285889 |
| TAG42:0-FA14:0 | 0.314854 | TAG55:5-FA20:4 | 0.26847 | TAG42:0-FA14:0 | 0.285658 |
| CE (16:0) | 0.303093 | TAG52:3-FA14:0 | 0.261238 | TAG52:4-FA20:2 | 0.252139 |
| PE (18:2/22:4) | 0.298692 | PE (18:2/22:4) | 0.201934 | TAG54:3-FA20:3 | 0.239438 |
| TAG51:2-FA17:0 | 0.296794 | TAG56:10-FA18:2 | 0.193153 |  |  |
| PE (18:2/20:5) | 0.291184 |  |  |  |  |
| TAG50:3-FA18:1 | 0.282342 |  |  |  |  |
| TAG54:3-FA16:0 | 0.268876 |  |  |  |  |
| PE (18:1/20:5) | 0.257723 |  |  |  |  |
| TAG56:2-FA16:0 | 0.252069 |  |  |  |  |
| TAG56:6-FA20:4 | 0.243474 |  |  |  |  |
| TAG56:6-FA20:3 | 0.213107 |  |  |  |  |
| TAG54:3-FA20:2 | 0.209603 |  |  |  |  |
| TAG58:7-FA20:4 | 0.209577 |  |  |  |  |
| DAG (16:0/20:3) | 0.196901 |  |  |  |  |

**Table S19.** The top 30 up-regulated and top 30 down-regulated changed lipids after CSE stimulation in 16-HBE cells following GPT2 overexpression.

| cell*^NC^*+CSE vs cell*^NC^* | | cell*^oeGPT2^* vs cell*^NC^* | | cell*^oeGPT2^*+CSE vs cell*^oeGPT2^* | |
| --- | --- | --- | --- | --- | --- |
| Lipids | FC | Lipids | FC | Lipids | FC |
| TAG56:4-FA20:3 | 20.93953 | TAG56:4-FA20:3 | 7.046474 | PC (18:0/20:1) | 4.878345 |
| CE (22:4) | 11.60394 | TAG52:4-FA18:3 | 5.938506 | PC (20:0/20:4) | 4.816342 |
| TAG50:5-FA18:2 | 5.380334 | TAG52:0-FA20:0 | 5.647068 | PE (14:0/22:4) | 3.484227 |
| TAG50:5-FA18:1 | 4.695195 | TAG50:5-FA20:4 | 5.498377 | PC (14:0/20:3) | 3.329114 |
| TAG56:5-FA18:2 | 3.46257 | TAG49:2-FA18:2 | 4.575216 | PC (18:2/20:2) | 3.124778 |
| PE (O-18:0/20:1) | 2.946361 | CE (22:4) | 3.729483 | PE (O-18:0/22:4) | 2.982777 |
| PE (O-18:0/22:6) | 2.936693 | TAG52:4-FA20:3 | 2.608455 | PE (O-16:0/22:4) | 2.969111 |
| PE (O-18:0/22:4) | 2.721146 | TAG54:4-FA18:2 | 2.397321 | PC (16:0/20:1) | 2.967256 |
| PE (O-16:0/22:6) | 2.595427 | TAG46:3-FA18:1 | 2.215695 | PC (18:0/22:4) | 2.839786 |
| TAG56:3-FA18:1 | 2.563875 | TAG56:5-FA18:1 | 2.212607 | PE (O-16:0/20:2) | 2.81304 |
| PE (O-18:0/20:2) | 2.517013 | TAG56:5-FA20:3 | 1.983122 | PE (18:0/20:2) | 2.745637 |
| PE (18:0/22:6) | 2.483651 | TAG54:3-FA18:1 | 1.946656 | PE (O-18:0/22:5) | 2.74316 |
| PE (16:0/22:6) | 2.422337 | TAG52:2-FA16:0 | 1.857166 | PE (14:0/22:6) | 2.676737 |
| PE (P-18:0/22:6) | 2.256273 | PC (18:0/16:1) | 0.635538 | PE (P-18:0/22:6) | 2.631348 |
| PE (O-16:0/22:4) | 2.17086 | PE (P-14:0/18:1) | 0.633926 | PE (O-16:0/22:5) | 2.591431 |
| PE (P-16:0/22:6) | 1.916 | PE (O-16:0/20:4) | 0.618263 | PE (O-16:0/22:6) | 2.58125 |
| PE (P-18:1/22:6) | 1.903676 | PC (16:0/16:0) | 0.614714 | PE (16:0/22:4) | 2.572423 |
| PE (P-18:1/18:3) | 1.875964 | PE (P-16:0/20:4) | 0.611301 | PE (P-18:0/22:4) | 2.532388 |
| PC (16:0/18:0) | 1.85139 | PC (16:0/16:1) | 0.607201 | PE (P-18:0/20:2) | 2.477675 |
| PE (18:1/22:6) | 1.783454 | PE (14:0/20:4) | 0.578896 | PE (O-18:0/22:6) | 2.449003 |
| PC (18:0/14:0) | 1.706596 | PC (16:0/22:6) | 0.573455 | TAG56:5-FA22:5 | 2.420863 |
| LPC (18:3) | 1.676543 | PC (16:0/14:0) | 0.57064 | PE (P-16:0/20:1) | 2.366717 |
| LPC (20:3) | 1.591111 | TAG50:2-FA14:0 | 0.552579 | PE (16:0/20:3) | 2.332985 |
| PE (18:2/20:2) | 1.537787 | PC (14:0/14:0) | 0.51469 | PC (18:0/20:3) | 2.325407 |
| PC (16:0/18:1) | 1.47373 | TAG48:1-FA16:0 | 0.496619 | PE (P-16:0/22:4) | 2.322693 |
| SM (18:0) | 0.799662 | LPC (16:0) | 0.493377 | PE (P-16:0/20:3) | 2.284414 |
| SM (14:0) | 0.754926 | PC (18:0/20:3) | 0.49296 | PC (18:1/20:3) | 2.225475 |
| PE (14:0/18:1) | 0.743382 | PE (18:2/20:2) | 0.481065 | PE (O-18:0/20:3) | 2.223801 |
| PE (P-14:0/18:1) | 0.716393 | TAG42:0-FA16:0 | 0.463176 | PE (18:0/22:4) | 2.197375 |
| SM (18:1) | 0.706877 | PE (18:0/20:2) | 0.444365 | PE (18:1/22:4) | 2.161235 |
| SM (20:1) | 0.683347 | PC (18:2/18:3) | 0.428983 | TAG58:7-FA18:1 | 0.532735 |
| PE (14:0/16:1) | 0.640766 | TAG56:7-FA16:0 | 0.396097 | CE (24:1) | 0.497784 |
| CER (14:0) | 0.467536 | PC (18:1/22:4) | 0.381427 | TAG42:2-FA18:2 | 0.465641 |
| TAG56:3-FA16:0 | 0.451605 | PE (14:0/20:1) | 0.369261 | TAG46:0-FA14:0 | 0.465019 |
| PE (14:0/20:4) | 0.411284 | TAG46:2-FA16:0 | 0.346651 | TAG50:3-FA14:0 | 0.440999 |
| TAG46:0-FA16:0 | 0.388335 | TAG46:0-FA16:0 | 0.299969 | TAG54:6-FA16:1 | 0.42409 |
| TAG48:3-FA16:0 | 0.35369 | TAG50:4-FA16:0 | 0.296853 | TAG44:1-FA18:1 | 0.402695 |
| TAG50:2-FA14:0 | 0.350372 | LPC (16:1) | 0.289745 | TAG48:3-FA14:0 | 0.399957 |
| TAG42:0-FA16:0 | 0.304148 | DAG (18:2/18:3) | 0.288186 | DAG (16:0/22:6) | 0.389189 |
| TAG52:6-FA16:1 | 0.290679 | PC (18:0/22:4) | 0.28294 | TAG50:5-FA16:1 | 0.361783 |
| TAG56:2-FA16:0 | 0.282611 | TAG56:3-FA16:0 | 0.218055 | TAG48:4-FA16:1 | 0.297957 |
| TAG58:7-FA16:0 | 0.252078 | TAG46:3-FA14:0 | 0.130434 | TAG42:0-FA16:0 | 0.29389 |
| DAG (18:2/20:5) | 0.235066 | PC (20:0/22:5) | 0.099617 | TAG42:1-FA16:1 | 0.292649 |
| TAG51:2-FA16:0 | 0.232607 |  |  | TAG46:3-FA18:1 | 0.281704 |
| TAG46:2-FA16:0 | 0.230617 |  |  | TAG52:5-FA22:5 | 0.247254 |
| TAG56:7-FA16:0 | 0.212784 |  |  | TAG51:3-FA16:1 | 0.237187 |
| CE (24:0) | 0.184928 |  |  | TAG46:3-FA16:1 | 0.190753 |
| DAG (18:2/18:3) | 0.183167 |  |  |  |  |
| TAG50:4-FA16:0 | 0.177233 |  |  |  |  |

**Table S20.** The top 30 up-regulated and top 30 down-regulated changed genes after CSE stimulation in 16-HBE cells following GPT2 knockdown.

| cell*^NC^*+CSE vs cell*^NC^* | | cell*^siGPT2^*+CSE vs cell*^siGPT2^* | | cell*^siGPT2^* vs cell*^NC^* | | cell*^siGPT2^*+CSE vs cell*^NC^*+CSE | |
| --- | --- | --- | --- | --- | --- | --- | --- |
| Genes | FC | Genes | FC | Genes | FC | Genes | FC |
| ETNPPL | 16.361324 | AL136295.1 | 30.935849 | AL356414.1 | 12.621985 | TNFRSF6B | 1382.675340 |
| GCKR | 14.657148 | MIR7-3HG | 20.721268 | CD209 | 12.033478 | PAEP | 16.334549 |
| XRCC6P1 | 12.549852 | ANKRD55 | 19.465315 | AC136475.3 | 11.715423 | LINC01771 | 13.996547 |
| AC106820.2 | 12.345210 | AMBP | 14.586731 | ANKRD55 | 10.653274 | LINC01914 | 13.874295 |
| AC092535.3 | 11.744236 | AKR1C1 | 12.982183 | GGTLC5P | 9.361569 | AL512604.2 | 13.102944 |
| AC092747.2 | 11.573556 | AC096536.2 | 12.165421 | Z85994.1 | 9.279054 | ATP8B4 | 11.529409 |
| RSPH10B2 | 11.039267 | AKR1C2 | 11.943684 | AC092139.2 | 9.278660 | AC020922.3 | 11.486724 |
| AC010719.1 | 10.994736 | ADGRG5 | 11.757111 | ESRRAP1 | 9.098648 | AC026362.2 | 11.131940 |
| GK3P | 10.752956 | CAVIN2-AS1 | 10.383102 | AL021154.1 | 8.977792 | CCDC114 | 11.109882 |
| AP005137.2 | 9.632197 | NECTIN4-AS1 | 10.188655 | AC005329.1 | 8.359276 | AC006126.4 | 10.595475 |
| DUSP13 | 9.500005 | AC016590.3 | 9.982742 | IL33 | 8.273109 | LINC01152 | 9.633164 |
| CD300LB | 9.100686 | GOLGA8UP | 9.781343 | TUBB7P | 8.224996 | C10orf131 | 8.609997 |
| AKR1C1 | 8.884755 | MYLPF | 9.128842 | CEACAM20 | 7.913667 | AL121768.1 | 8.426764 |
| SOX21-AS1 | 8.664577 | MMP3 | 8.400629 | GPLD1 | 7.206164 | RP11-291I6.2 | 8.373691 |
| KLHL30-AS1 | 8.620047 | ATF4P2 | 8.255725 | AL022157.1 | 6.998299 | NLRP11 | 8.234372 |
| AC022784.6 | 8.620047 | FTH1P5 | 8.141636 | FOXP1-IT1 | 6.998299 | AC134772.1 | 8.234372 |
| AC020661.1 | 8.491136 | CADM2 | 8.097977 | SLC28A3 | 6.678292 | EEF1DP3 | 8.145757 |
| TIPARP-AS1 | 8.395122 | HMOX1 | 7.989609 | ZNF253 | 6.602684 | CYP4A22-AS1 | 7.597076 |
| AP003733.3 | 8.052654 | NAGPA-AS1 | 7.983888 | LINC02827 | 6.534746 | OPCML | 7.323023 |
| TREML3P | 7.898133 | EID3 | 7.752534 | HRC | 6.381237 | MST1P2 | 7.212995 |
| AC233280.1 | 7.893679 | SP9 | 7.647389 | AC007292.2 | 6.380931 | C20orf141 | 7.112890 |
| MMP3 | 7.802428 | FSTL4 | 7.440753 | AC007906.1 | 5.935729 | SELENOKP1 | 6.973572 |
| FBXW10 | 7.621761 | GLTPP1 | 7.440753 | GOLGA8UP | 5.923996 | C15orf56 | 6.848165 |
| AKR1C2 | 7.527630 | LINC01703 | 7.339068 | SERPINB10 | 5.860028 | LRG1 | 6.658282 |
| IL13RA2 | 7.466207 | HCG25 | 7.152906 | AC125494.2 | 5.780115 | C2orf50 | 6.583035 |
| AP006623.1 | 7.359978 | AL132655.1 | 7.101570 | FRRS1L | 5.780115 | PDE8B | 6.307642 |
| AC097460.2 | 7.215820 | DLGAP1 | 6.998495 | ALOX12P1 | 5.780115 | AC060766.6 | 6.220418 |
| AL121772.1 | 7.196748 | AC084782.1 | 6.840747 | AC108704.1 | 5.779300 | AL049833.1 | 6.220418 |
| LRRC43 | 6.149667 | CPZ | 6.812788 | BX649632.1 | 5.779300 | TMEM178A | 6.004655 |
| NMRAL2P | 6.133198 | HCP5 | 6.752393 | NDUFAF4P1 | 5.779300 | NIBAN3 | 5.916794 |
| SLAMF8 | 0.048048 | CCDC85A | 0.051961 | C10orf143 | 0.056937 | AC017083.3 | 0.071028 |
| KIF6 | 0.050885 | CAVIN2 | 0.059826 | KRT78 | 0.072703 | C2CD4D | 0.076642 |
| TMEM119 | 0.052131 | AC099518.4 | 0.065521 | DUX4L50 | 0.099391 | MIR193BHG | 0.088740 |
| BPI | 0.055220 | GBP6 | 0.073994 | AP006623.1 | 0.099925 | AC084035.1 | 0.091024 |
| AC068860.1 | 0.055999 | LINC01914 | 0.075248 | AL157770.1 | 0.105333 | KIF6 | 0.093277 |
| AC007743.1 | 0.057383 | SAA2-SAA4 | 0.081448 | RP11-465N4.5 | 0.110646 | LINC01715 | 0.105097 |
| AGAP11 | 0.069081 | ZNF709 | 0.085386 | BTK | 0.111817 | GGCTP1 | 0.105803 |
| USHBP1 | 0.071547 | AC006126.4 | 0.085925 | COL22A1 | 0.113692 | CD7 | 0.117037 |
| AP000892.4 | 0.079579 | CHRNA1 | 0.086872 | CFAP74 | 0.116303 | SPAAR | 0.119648 |
| KLHDC7A | 0.081483 | PDE8B | 0.087259 | ZDHHC20-IT1 | 0.117373 | AC110048.2 | 0.125574 |
| AC017083.3 | 0.086070 | CCDC114 | 0.091003 | AC244453.2 | 0.134003 | ZNF736P9Y | 0.125604 |
| ZBP1 | 0.087326 | FUT2 | 0.092229 | AL132800.1 | 0.145912 | PSMD8P1 | 0.128771 |
| MIR193BHG | 0.087451 | GPR88 | 0.092464 | AL512625.2 | 0.150554 | NMBR | 0.130237 |
| AC005083.1 | 0.090286 | NIBAN3 | 0.094769 | AC027088.1 | 0.155251 | LINC01792 | 0.142455 |
| CAVIN2 | 0.091193 | NAT2 | 0.097317 | CXorf58 | 0.155566 | AC244453.2 | 0.144886 |
| BCRP3 | 0.091570 | OPRD1 | 0.103692 | PVRIG2P | 0.155929 | AC004923.4 | 0.145470 |
| SH3GL1P2 | 0.091572 | ICA1 | 0.109920 | PTGIS | 0.156202 | EFCAB8 | 0.145502 |
| C5orf46 | 0.096487 | DKK2 | 0.118093 | ZNF32-AS2 | 0.156205 | AC015909.1 | 0.148330 |
| LINGO1 | 0.099525 | ISLR | 0.118975 | HSPE1P7 | 0.159556 | EIF5P1 | 0.148344 |
| RPL41P2 | 0.100257 | LRG1 | 0.119246 | AC069368.1 | 0.164065 | GNMT | 0.158551 |
| FCGR2A | 0.102291 | AL049634.2 | 0.120198 | AC004383.1 | 0.171847 | SERPINA3 | 0.160745 |
| CELF4 | 0.108151 | CXCL10 | 0.123062 | AL354798.1 | 0.180545 | GZMA | 0.162180 |
| VNN2 | 0.108586 | PRR34 | 0.129670 | LYG2 | 0.182252 | ENO1-AS1 | 0.169231 |
| EPB41L4A | 0.109921 | GPD1 | 0.131425 | AL033523.1 | 0.182252 | AC104521.1 | 0.169231 |
| AL513534.2 | 0.110611 | OPCML | 0.134520 | NOTO | 0.183125 | DLGAP1 | 0.169231 |
| TNK2-AS1 | 0.111182 | ACTG2 | 0.135744 | THORLNC | 0.183165 | AC133550.3 | 0.171358 |
| NR1I2 | 0.114528 | SYPL1P2 | 0.136829 | AC062015.1 | 0.184104 | AC005821.1 | 0.175471 |
| WASIR2 | 0.116268 | HSH2D | 0.139865 | GK3P | 0.199851 | DNAAF1 | 0.176807 |
| AL359715.4 | 0.116274 | KRT87P | 0.141658 | FGF14-AS2 | 0.199851 | SHBG | 0.184555 |
| AC015909.1 | 0.116890 | DNAJB13 | 0.142643 | LNX1-AS2 | 0.209442 | AC099811.3 | 0.191314 |

**Table S21.** The top 30 up-regulated and top 30 down-regulated changed lipids in lung tissue of mice.

| COPD vs Control | | COPD+AOA vs Control | | COPD vs COPD+AOA | |
| --- | --- | --- | --- | --- | --- |
| Lipids | FC | Lipids | FC | Lipids | FC |
| TAG58:7-FA20:4 | 6.42 | DAG (18:2/18:3) | 2.7639 | PE (P-16:0/18:3) | 2.980301 |
| PC (18:2/22:4) | 5.102916 |  |  | PE (P-18:1/18:3) | 2.280707 |
| TAG56:6-FA20:4 | 2.851079 |  |  | PE (16:0/20:5) | 2.267338 |
| TAG58:10-FA20:4 | 2.735308 |  |  | CER (16:0) | 2.263395 |
| PE (14:0/16:1) | 2.666221 |  |  | PC (18:1/20:1) | 2.251704 |
| PE (P-18:0/18:2) | 2.54092 |  |  | TAG58:9-FA22:5 | 2.225571 |
| PE (14:0/18:1) | 2.468708 |  |  | PE (P-18:0/18:2) | 2.186042 |
| PE (16:0/18:3) | 2.378809 |  |  | PE (14:0/16:1) | 2.157775 |
| PE (P-18:1/18:3) | 2.344408 |  |  | PC (18:0/20:1) | 2.119844 |
| TAG56:5-FA20:4 | 2.328911 |  |  | PC (18:2/20:2) | 2.105205 |
| PC (14:0/14:0) | 2.321145 |  |  | TAG58:9-FA20:4 | 1.94195 |
| PC (18:0/22:6) | 2.304436 |  |  | PC (18:1/18:3) | 1.903204 |
| PE (16:0/20:5) | 2.280139 |  |  | PE (P-14:0/18:1) | 1.846522 |
| PE (P-16:0/20:5) | 2.223476 |  |  | PC (18:0/18:0) | 1.844096 |
| PC (18:0/22:4) | 2.196392 |  |  | PE (P-16:0/18:2) | 1.815517 |
| PC (18:0/18:0) | 2.178665 |  |  | PC (18:1/18:1) | 1.79388 |
| PE (P-18:0/18:1) | 2.154055 |  |  | PE (18:1/22:6) | 1.788579 |
| PC (14:0/18:2) | 2.075658 |  |  | PC (18:0/18:1) | 1.781963 |
| PE (P-18:1/18:1) | 2.036904 |  |  | PC (18:0/16:1) | 1.774266 |
| PE (16:0/22:6) | 1.999961 |  |  | PC (16:0/22:4) | 1.756084 |
| PC (18:0/18:1) | 1.983737 |  |  | PE (P-16:0/18:0) | 1.715186 |
| PC (16:0/18:3) | 1.963121 |  |  | PE (18:0/22:6) | 1.713837 |
| PE (P-16:0/18:0) | 1.962323 |  |  | LPC (20:0) | 1.710841 |
| PC (14:0/20:4) | 1.958534 |  |  | PC (14:0/18:1) | 1.657348 |
| PE (P-16:0/18:1) | 1.930062 |  |  | PC (18:0/22:4) | 1.593283 |
| PE (P-16:0/18:2) | 1.910883 |  |  | PC (16:0/18:1) | 1.580626 |
| TAG58:9-FA20:4 | 1.861498 |  |  | PC (16:0/18:0) | 1.572987 |
| PC (18:0/22:5) | 1.825801 |  |  | PC (16:1/18:1) | 1.53641 |
| PC (18:0/14:0) | 1.817759 |  |  | PE (18:2/18:3) | 1.335197 |
| PE (P-18:0/20:3) | 1.806325 |  |  |  |  |
| PE (18:2/20:1) | 0.601132 |  |  |  |  |
| TAG47:2-FA18:1 | 0.158095 |  |  |  |  |

**Table S22.** Differential metabolites in lung tissue of mice.

| COPD vs Control | | COPD+AOA vs Control | | COPD vs COPD+AOA | |
| --- | --- | --- | --- | --- | --- |
| Metabolites | FC | Metabolites | FC | Metabolites | FC |
| L-glutamic acid | 21.60735 | D-glucose | 5.393911 | palmitic acid | 6.988355 |
| 1,3-propanediol | 20.88968 | allo-inositol | 4.26023 | L- (-)-fucose | 6.080684 |
| phosphoric acid | 17.21606 | lactamide | 4.188928 | L-glutamic acid | 5.597811 |
| oxalic acid | 16.33662 | glycolic acid | 4.103438 | citric acid | 4.290206 |
| palmitic acid | 15.76852 | 1,3-propanediol | 3.882174 | L-mimosine | 3.52551 |
| ethanolamine | 14.54711 | L-glutamic acid | 3.859964 | threose | 2.843648 |
| conduritol epoxide | 14.23953 | phosphoric acid | 3.610944 |  |  |
| L-mimosine | 12.14826 | L- (+) lactic acid | 3.5421 |  |  |
| citric acid | 11.98178 | threose | 3.521788 |  |  |
| threose | 10.01472 | oxalic acid | 3.489139 |  |  |
| L- (+) lactic acid | 9.824442 | L-mimosine | 3.445816 |  |  |
| glycolic acid | 9.451265 | acetohydroxamic acid | 3.246646 |  |  |
| L- (-)-fucose | 6.046401 | ethanolamine | 3.231574 |  |  |
|  |  | glycine | 2.417178 |  |  |
|  |  | palmitic acid | 2.256399 |  |  |

**Table S23. The detail information of genes.**

| Abbreviation | Full name | Genetic location |
| --- | --- | --- |
| GLUD | glutamate dehydrogenase | chr10:87,050,202-87,094,843 |
| GOT | glutamic-oxaloacetic transaminase | chr10:99,396,870-99,430,624 |
| GPT | glutamic--pyruvic transaminase | chr8:144,502,973-144,507,174 |
| PSAT1 | phosphoserine aminotransferase 1 | chr9:78,297,125-78,330,093 |
| ACLY | ATP citrate lyase | chr17:41,866,917-41,930,545 |
| AST | aspartate aminotransferase | chr6:73,593,379-73,653,992 |
| CHPT1 | choline phosphotransferase 1 | chr12:101,696,947-101,744,140 |
| SLC7A11 | solute carrier family 7 member 11 | chr4:138,164,097-138,312,671 |
| SELENOI | selenoprotein I | chr2:26,308,547-26,395,885 |
| DGAT1 | diacylglycerol O-acyltransferase 1 | chr8:144,314,584-144,326,910 |
| PCTP | phosphatidylcholine transfer protein | chr17:55,751,051-55,852,715 |
| SLC44A1 | solute carrier family 44 member 1 | chr9:105,244,622-105,439,171 |
| SLC44A3 | solute carrier family 44 member 3 | chr1:94,820,342-94,895,247 |
| ACHE | acetylcholinesterase | chr7:100,889,994-100,896,994 |
| PLA2G4D | phospholipase A2 group IVD | chr15:42,067,009-42,094,562 |
| STARD10 | STAR related lipid transfer domain containing 10 | chr11:72,754,729-72,794,047 |
| CHKB | choline kinase beta | chr22:50,578,959-50,601,455 |
| CSNK2A1 | casein kinase 2 alpha 1 | chr20:472,498-543,835 |
| PCYT2 | phosphate cytidylyltransferase 2, ethanolamine | chr17:81,900,958-81,911,432 |
| LPIN2 | lipin 2 | chr18:2,885,296-3,013,144 |
| CHKA | choline kinase alpha | chr11:68,052,859-68,121,444 |
| GK | glycerol kinase | chrX:30,653,359-30,731,462 |
| PNPLA4 | patatin like phospholipase domain containing 4 | chrX:7,898,247-7,927,739 |
| GPAM | glycerol-3-phosphate acyltransferase, mitochondrial | chr10:112,149,865-112,227,677 |
| PRKACA | protein kinase CAMP-activated catalytic subunit alpha | chr19:14,091,688-14,118,084 |
| PPP1CC | protein phosphatase 1 catalytic subunit gamma | chr12:110,708,376-110,742,939 |
| PSAT | phosphoserine aminotransferase | chr9:78,297,125-78,330,093 |
