# Supplementary material for: Roles of glutamic pyruvate transaminase 2 in reprogramming of airway epithelial lipidomic and metabolomic profiles after smoking
Source: Clin Transl Med. 2024 May 5;14(5):e1679. doi: 10.1002/ctm2.1679 (PMC11070440; doi:10.1002/ctm2.1679)
Supplement: Supplementary file 7 — Supporting Information [file CTM2-14-e1679-s002.docx]

**Legends to Supplementary Figures**

**Figure S1. Expression of regulatory enzyme transcription genes in glutamate metabolism pathway after knockdown of GPT2.** After knocking down the expression of GPT2, the mRNA levels of other transcriptional genes regulating enzymes in the pathway were detected (A-D). The results showed that changes in the level of GPT2 did not affect the expression of other enzymes. Each group contains 6 samples. **p* < 0.05; ***p* < 0.01; ****p* < 0.001, compared to 0% CSE group.

**Figure S2. Pathway enrichment analysis of RNA-*seq* results for significantly altered genes.** In our RNA-*seq* results, the differentially expressed genes in the 6% CSE-stimulated group compared with the vehicle group were enriched by pathway enrichment, and the results were analyzed by GSEA. We identified enrichment datasets using four different datasets (KEGG, GO, Hallmark, and Reactome, A-D).

**Figure S3. Expression of endoplasmic reticulum stress-related genes in cells after changing GPT2 expression.** After knockdown or overexpression of GPT2 expression, the mRNA levels of endoplasmic reticulum stress-related genes (ATF6, IRE1, and PERK) were detected (A-F). Each group contains 6 samples. **p* < 0.05; ***p* < 0.01; ****p* < 0.001, compared to 0% CSE group. Contrasts between multivariate variables are indicated by horizontal bars.

**Figure S4. Major metabolic and biosynthetic process of Glutamate.**

Glutamate into and out of the mammalian cell through glutamate transporters. Glutamate itself can contribute to the De novo synthesis of nucleic acids, protein folding, and lipid synthesis. Glutamate also could take part in the synthesis of glutamine and glutathione. Glutamate changes into α-KG by one of two sets of enzymes, GLUD1/2 or aminotransferases (GOT1/2, GPT1/2, and PSAT1). α-KG joins in the TCA cycle and could provide energy to cells. Oxaloacetic acid (OAA) can be converted to aspartate for supporting nucleotide synthesis.
